# Systematic meta-analyses and field synopsis of genetic and epigenetic studies in paediatric inflammatory bowel disease

**DOI:** 10.1038/srep34076

**Published:** 2016-09-27

**Authors:** Xue Li, Peige Song, Maria Timofeeva, Xiangrui Meng, Igor Rudan, Julian Little, Jack Satsangi, Harry Campbell, Evropi Theodoratou

**Affiliations:** 1 Centre for Population Health Sciences, University of Edinburgh, Edinburgh, United Kingdom; 2 Colon Cancer Genetics Group and Academic Coloproctology, Institute of Genetics and Molecular Medicine, University of Edinburgh and MRC Human Genetics Unit Western General Hospital Edinburgh, Edinburgh, United Kingdom; 3 School of Epidemiology, Public Health and Preventive Medicine, University of Ottawa, Ottawa, Canada; 4 Centre for Genomic and Experimental Medicine, Institute of Genetics and Molecular Medicine, University of Edinburgh and Western General Hospital Edinburgh, Edinburgh, United Kingdom

## Abstract

We provide a comprehensive field synopsis of genetic and epigenetic associations for paediatric Inflammatory Bowel Disease (IBD). A systematic review was performed and included 84 genetic association studies reporting data for 183 polymorphisms in 71 genes. Meta-analyses were conducted for 20 SNPs in 10 genes of paediatric Crohn’s disease (CD) and for 8 SNPs in 5 genes of paediatric ulcerative colitis (UC). Five epigenetic studies were also included, but formal meta-analysis was not possible. Venice criteria and Bayesian false discovery probability test were applied to assess the credibility of associations. Nine SNPs in 4 genes were considered to have highly credible associations with paediatric CD, of which four variants (rs2066847, rs12521868, rs26313667, rs1800629) were not previously identified in paediatric GWAS. Differential DNA methylation in *NOD2* and *TNF-α*, dysregulated expression in let-7 and miR-124 were associated with paediatric IBD, but not as yet replicated. Highly credible SNPs associated with paediatric IBD have also been implicated in adult IBD, with similar magnitudes of associations. Early onset and distinct phenotypic features of paediatric IBD might be due to distinct epigenetic changes, but these findings need to be replicated. Further progress identifying genetic and epigenetic susceptibility of paediatric IBD will require international collaboration, population diversity and harmonization of protocols.

Crohn’s disease (CD) and ulcerative colitis (UC), known as inflammatory bowel diseases (IBD), are chronic inflammatory gastrointestinal disorders that most commonly arise in adolescents and young adults^1^. In Europe and North America, the estimated prevalence of CD is 50–200/100,000 and of UC 120–200/100,000^2^. An increasing incidence of IBD has been observed in both developed and developing countries^2^. Up to 25% of IBD incident cases occur in childhood and result in retarded growth, impaired skeletal development, and lack of weight gain or even abnormal weight loss^3^.

The increasing incidence and prevalence with decreased quality of life in child IBD patients has spurred ongoing search for risk factors of paediatric IBD. Familial, twin and phenotypic concordance studies suggest that IBD is highly heritable^4^,^5^,^6^. A large number of candidate gene studies have been performed to investigate the complex genetic architecture underlying paediatric IBD risk. However, due to the difficulty of recruiting affected children because of the relatively low prevalence, most of the paediatric genetic association studies reported to date had relatively small sample sizes or have included predominantly young-adult subjects, which may severely limit their power to distinguish genetic susceptibility patterns in paediatric IBD that are distinct from that of adult-onset disease. To date, genome wide association studies (GWAS) of IBD have identified a total of 163 loci, including 140 loci associated with CD and 133 associated with UC^7^. To detect susceptibility genes related to early disease onset, two large GWAS have been reported with cases diagnosed before the age of 19 years, and identified 7 novel loci which were thought to be exclusive to early-onset IBD^8^,^9^. However, further meta-analysis of adult GWAS showed that these 7 loci were also implicated in adult-onset disease^7^.

Although genetic studies have identified a large number of IBD risk loci, the majority of the identified variants are located in noncoding regions and explain a small proportion of the variance in disease risk (13.6% for CD and 7.5% for UC)^7^. Epigenetic changes may mediate interactions between genes and environmental factors such as the gut microbiome, smoking and infection, and thus might explain some of the missing heritability^10^. For paediatric IBD specifically, epigenetic changes through exposures in utero, perinatal and early-life stage to environmental agents may at least partly explain the early disease onset^11^.

We report a comprehensive review of genetic and epigenetic studies in paediatric IBD, using the guidelines established by Human Genome Epidemiology Network (HuGENet)^12^. We reviewed all candidate-gene association studies and performed meta-analyses for variants with genotype data available in at least three studies. A well-defined framework was employed to assess the credibility of the associations and thus to identify the genetic variants with robust evidence of association with paediatric IBD. In addition, we systematically reviewed and summarised reported epigenetic associations with paediatric IBD; a formal meta-analysis could not be performed because of differences between studies in types of tissue investigated and assays used.

## Methods

### Literature search and selection criteria

We searched three databases (Medline, EMBASE and the HuGENet web resource) (Supplementary Table S1). Studies were screened for eligibility independently by two authors (XL and PS) based on a pre-defined set of inclusion and exclusion criteria. Case–control and cohort studies of associations between paediatric IBD and SNPs, DNA methylation or miRNA expression were potentially eligible for inclusion. Paediatric IBD cases were defined as patients diagnosed in childhood or adolescence (i.e. before the age of 18 years). Articles that did not specify the age structure of their study populations, family-based studies, and studies that only investigated disease progression, pathological or histological phenotype were excluded. Studies that did not provide sufficient information about genotype or allele distribution of variants, and studies published in abstract form only, were excluded.

### Data extraction and management

For each study, the following data were extracted by two authors (XL and PS) independently: (1) identification details: the authors, publication year, study location, study design, sample size, age cut-off of paediatric IBD, diagnosis criteria, and sample source; (2) participant characteristics: age of disease onset, age structure of study population, and ethnicity; (3) genetic information: genes, polymorphisms, reference allele, allele frequency, allelic and genotypic counts; (4) epigenetic changes: loci with differential DNA methylation and miRNAs with dysregulated expression. Double extracted data were checked by a third author (XM) and any discrepancies were resolved by discussion.

### Statistical analysis for genetic association studies

Statistical analysis was conducted using the R software (R x64 3.0.1). Meta-analyses were performed only for the variants with data available from at least three independent studies. Summary crude odds ratios (ORs) and 95% confidence intervals (95% CI) for four genetic models were calculated by using either the fixed-effect or random-effect model^13^. When there was no significant heterogeneity, the fixed-effect model was applied; otherwise the random-effect model. The Q statistic (with a threshold of *p*-value <0.05) and I^2^ metric were calculated to quantify heterogeneity. Funnel plot analysis with an Egger test was conducted to test for small study effect. Statistical power was estimated by the Power and Sample Size Program. Hardy–Weinberg equilibrium (HWE) in controls was analysed by chi-square or Fisher’s exact test for each individual study. Sensitivity analysis was performed by excluding studies violating HWE or studies about non-white populations. Linkage disequilibrium (LD) between SNPs in the same genes was checked using the databases of 1000 Genomes (http://www.1000genomes.org/).

### Credibility of genetic association

We applied the Venice criteria^12^ and Bayesian false-discovery probability (BFDP)^14^ to assess the credibility of the observed associations.

With Venice criteria, the credibility of associations was assessed for three criteria: the amount of evidence, the extent of replication and protection from bias^12^. We used statistical power to assess the volume of evidence; a grade of A, B, and C was assigned respectively when statistical power was greater than 80%, 50–79%, or less than 50%. The extent of replication was assessed by the measurement of heterogeneity (I^2^ value): A, 25% or less; B, 25–49%; C, 50% or more. For protection from bias, complete assessment is difficult. We assigned “B” to associations for which no small study effect was detected; otherwise, “C” was assigned.

BFDP was estimated to assess the noteworthiness of observed associations by using the Excel Calculation Spreadsheet (http://faculty.washington.edu/jonno/cv.html) on a prior level of 0.05^14^. A noteworthy threshold was defined as 0.2 based on the assumption that the cost of false discovery would be four times higher than a false non-discovery.

Genetic associations were classified into three categories based on the above assessment. Associations were classified as “highly credible” when (1) the association was statistically significant (*p*-value <0.05) in at least two genetic models; (2) the BFDP value was less than 0.20 ; (3) the statistical power was greater than 80%; and (4) I^2^ was less than 50%. “Less credible” associations were defined as associations that were statistically significant (*p*-value <0.05) in at least one genetic model, with BFDP value greater than 0.20, and statistical power between 50% and 79%. All other associations were considered to be “not credible”.

## Results

### Genetic association studies

#### Study characteristics

**Figure 1:**
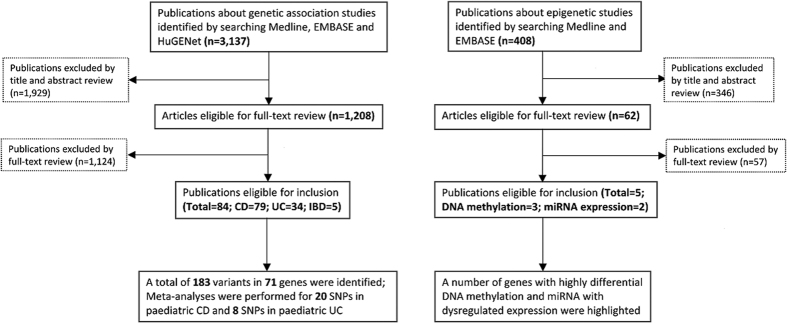
Flow charts for the selection of eligible genetic and epigenetic studies.

A total of 3,137 citations were identified. After title and abstract review, 1,208 publications were reviewed in full text; 84 publications were finally included, 79 of which were on paediatric CD, 34 on paediatric UC and 5 on paediatric IBD (Fig. 1). Characteristics of the included studies are presented in Supplementary Table S2. Of these, the majority of the studies (n = 57, 67.9%) were conducted in pediatric or early-onset IBD patients younger than 17 years, while 10 studies (11.9%) focused on IBD patients below 16 years and 17 studies (20.2%) focused on IBD patients below 18 years. The geographic distribution of the study populations (Supplementary Fig. S1) suggests a lack of population diversity in exploring the genetic factors of paediatric IBD, since more than 90% of the studies (n = 78) were conducted among white populations and only a small number of studies were conducted in Asian (n = 6, 7.6%) and African American (n = 1, 1.3%).

One hundred and eighty three polymorphisms in 71 genes were identified (147 polymorphisms in 58 genes in paediatric CD, 80 polymorphisms in 40 genes in paediatric UC, and 29 polymorphisms in 8 genes in paediatric IBD). Thirty five variants were found to be positively associated with paediatric CD in genetic association studies and twenty of them (57.1%) were confirmed in GWAS. Fifteen variants were identified to have positive associations with paediatric UC and ten of them (66.7%) were confirmed in GWAS (Supplementary Table S3).

#### Meta-analyses results

Meta-analyses were conducted for 20 variants in paediatric CD and 8 variants in paediatric UC, for which data were available in three or more independent studies (Table 1). Meta-analyses of paediatric CD included 7 (mean; range: 3–16) independent studies with 854 (mean; range: 165–2059) cases and 2137 (mean; range: 369–6385) controls. Meta-analyses of paediatric UC included 4 (mean; range: 3–6) independent studies with 385 (mean; range: 318–606) cases and 1039 (mean; range: 800–1515) controls. For two variants (rs2066844, rs2241880) in paediatric CD and 1 variant (rs2066844) in paediatric UC, some of the studies violated HWE (*p* < 0.05). For two variants (rs2066844, rs2066847) in paediatric CD, one studies was conducted in African Americans. Four pairs of SNPs were found in LD with r^2^ threshold ≥0.60 (Supplementary Table S4).

#### Paediatric CD

Meta-analyses of 20 variants in four genetic models for paediatric CD are presented in Tables 2 and 3. Eleven variants had summary ORs with *p*-value <0.05 in at least two genetic models. Associations with 9 variants (rs2066844, rs2066845, rs2066847, rs11209026, rs7517847, rs12521868, rs26313667, rs2241880 and rs1800629) were considered as the most highly-credible, due to the high statistical power (>0.80), small heterogeneity (I^2^ < 50%) and low BFDP (<0.20); the other 2 variants (rs11739135, rs1050152) were considered as less-credible SNPs. ORs and 95% CI of individual studies for each variant and each model are shown in forest plots in Supplementary Fig. S2–S21. Funnel plots with Egger tests are shown in Supplementary Fig. S22–S41.

Among the highly credible variants, rs2066844, rs2066845, rs11209026, rs7517847 and rs2241880 achieve genome-wide significance in both paediatric and adult-onset IBD GWAS (Table 4). Rs2066847 and rs12521868 were found to be genome-wide significant only in adult-onset IBD GWAS. Rs26313667 and rs1800629 have not been previously identified in either paediatric or adult-onset IBD GWAS.

#### Paediatric UC

Tables 5 and 6 present the results of meta-analyses of 8 variants for paediatric UC. Four variants (rs2066844, rs2066847, rs11209026, rs1800629) had significant associations with *p*-values <0.05 in at least two genetic models, but none of them were classified as highly-credible associations either because of the high BFDP (>0.20) or low statistical power (<0.80). ORs and 95% confidence intervals of individual studies for each variant and each model are shown in forest plots in Supplementary Fig. S42–S49. Funnel plots with Egger tests are shown in Supplementary Fig. S50–S57.

#### Sensitivity Analysis

Sensitivity analysis was conducted in three variants (rs2066844, rs2066847, rs2241880) for paediatric CD and one variant (rs2066844) for paediatric UC by excluding a study of non-white population and a study with variants violating HWE (Supplementary Tables S5–S8). Sensitivity analyses did not reveal major differences in genetic effects. Rs2066844, rs2066847 and rs2241880 remained statistically significant (*p* < 0.05) with small changes in summary ORs and credibility assessment showed no inconsistencies with the previous analysis.

#### Epigenetic studies

After screening 408 titles and abstracts, 62 papers were reviewed in full text. Five studies met the inclusion and exclusion criteria, with three of them focusing on changes in DNA methylation and two investigating miRNA expression changes (Fig. 1, Supplementary Table S9, Table 7). Genome-wide DNA methylation analysis in peripheral blood leukocyte was performed in two studies using the same methylation platform; there was no overlap in differential methylated genes. A DNA methylation study in intestinal biopsies highlighted 6 genes which were associated with colonic mucosal immune and defence responses. In addition, dysregulated expression in 26 miRNAs was observed in paediatric IBD cases, two of which (miR-124 and let-7) were reported in both paediatric studies.

## Discussion

This study offers a comprehensive and up-to-date assessment of genetic and epigenetic literature in paediatric IBD. We systematically evaluated data from 84 studies for 183 variants in 71 candidate genes. It differs from other meta-analyses of genetic effect on IBD^15^,^16^,^17^, since it integrates information of all variants that were investigated in genetic association studies of paediatric IBD. We only included studies conducted in children younger than 18 years old and not studies considering patients 19–21 years old or adults. This study is the first field synopsis of paediatric IBD with the advantage of evaluating both genetics and epigenetic associations. A well-defined assessing framework, including the Venice criteria and BFDP, were used to classify the identified associations. After credibility assessment, this study indicated three variants (rs2066844, rs2066845, rs2066847) in *NOD2*, two variants (rs11209026, rs7517847) in *IL23R*, one variant (rs2241880) in *ATG16L1*, two variants (rs12521868, rs26313667) in the *IBD5* region and one variant (rs1800629) in *TNF-α* represented the most credible findings and were referred as highly-credibly associated with paediatric IBD. Four (rs2066847 in *NOD2*, rs12521868 and rs26313667 in *IBD5*, rs1800629 in *TNF-α*) of these variants have not been identified in the GWAS of paediatric IBD. In addition, all of the identified variants have been found also to be associated with adult IBD either by candidate gene studies or GWAS.

The contribution of *NOD2* gene to CD risk has been confirmed in multiple GWAS of European ancestry adults^7^. Our study further supported the evidence that three SNPs (rs2066844, rs2066845, rs2066847) in *NOD2* were each highly associated with increased paediatric CD risk. Compared with a meta-analysis of adult-onset CD, the effect sizes of these three SNPs in children were similar to those in adults^18^. The summary ORs of rs2066844, rs2066845 and rs2066847 variants estimated for paediatric CD in this study were 2.23 (95% CI: 1.88–2.64), 3.21 (95% CI: 2.25–4.6) and 5.78 (95% CI: 3.67–9.11) respectively. The equivalent summary ORs for adult onset CD associated with these variants were 2.20 (95% CI: 1.84–2.62), 2.99 (95% CI: 2.38–3.74) and 4.09 (95% CI: 3.23–5.18) respectively^18^, suggesting no stronger genetic associations in children. In contrast, the association between the *NOD2* gene and UC remains somewhat controversial. Most of the available data suggest no association between *NOD2* mutations and UC susceptibility. The findings of this study indicated that the frequency of rs2066844 and rs2066847 variants was significantly higher in paediatric UC patients than in healthy children suggesting a possible involvement of *NOD2* in paediatric UC. However, due to the limited statistical power and high probability of false discovery, this association between the *NOD2* gene variant and paediatric UC susceptibility is considered as “less credible” and needs further study and elucidation.

The association of *IL23R* with adult IBD risk was established in a GWAS^19^, and then was confirmed in genome wide meta-analysis^7^. A paediatric GWAS conducted by Imielinski *et al*. replicated this association in children^8^. Consistent with these findings, our study confirmed the *IL23R* association in both paediatric CD and UC. It was estimated that the rs11209026 variant conferred approximately a 3-fold reduction in the risk of paediatric CD. For paediatric UC, the protective effect was significant but less-credible (OR = 0.54; 95% CI: 0.35–0.84) due to the high probability of false discovery. In addition, homozygosity for the rs7517847 variant was also credibly associated with paediatric CD (OR = 0.47, 95% CI: 0.32–0.67). A recent meta-analysis of rs7517847 estimated an odds ratio of 0.49 (95% CI: 0.38–0.64) for adult-onset CD risk^15^, suggesting no substantial difference in genetic effect size between paediatric and adult-onset CD.

*IBD5* region encompasses several candidate genes which were initially suggested to be associated with early-onset IBD^20^. The study showed *IBD5* region had strong association with early-onset CD, but only exerted a moderate effect on adult-onset CD. Subsequently, Mirza *et al*. reported a correlation between genes in *IBD5* region and age of CD onset^21^. In the present study, we confirmed the significant associations of two variants (rs26313667 and rs12521868) in the *IBD5* region with paediatric CD, but no stronger effects than those observed in adult population. Variant homozygotes of rs26313667 had an estimated OR of 1.68 (95% CI: 1.26–2.23), which was not significantly greater than that in adult-onset CD (OR = 1.39; 95% CI: 1.15–1.67)^16^. Our finding was in concordance with the study of Wang J *et al*., which reported an effect size of 1.68 (95% CI: 1.21–2.33) for children^16^. In addition, a highly credible association was observed with homozygosity for rs12521868, with a summary OR of 1.88 (95% CI: 1.34–2.62), similar to the reported magnitude of association for adults (OR = 1.83; 95% CI: 1.54–2.71)^16^. Therefore, we conclude that variants within the *IBD5* region increase CD susceptibility with no distinct specification to paediatric CD. Of particular note, identification of the precise causal gene in *IBD5* region has proved to be challenging, due to the extensive degree of linkage disequilibrium between the examined polymorphisms.

In the *ATG16L1* gene, the rs2241880 polymorphism was identified to be associated with paediatric CD. Association of rs2241880 with adult-onset CD has been confirmed in multiple GWAS^7^. Association of this variant with paediatric CD were also established in two GWAS conducted among children^8^,^9^. Our study further reinforced this association with paediatric CD based on evidence from candidate gene studies. A recent meta-analysis reported an OR of 0.71 (95% CI: 0.53-0.95) in paediatric CD homozygotes^22^. This finding was similar to our study (OR = 0.61, 95%CI: 0.51–0.74). It is noteworthy that the common variant of rs2241880 confers an increase in disease risk (frequency of risk allele G = 0.63). *ATG16L1* is involved in autophagy and plays an important role in the degradation of intracellular pathogens and antigen processing^23^. Therefore, a possible explanation for the increased risk may be that while this common variant functions as a protective factor against infection, it may increase the risk of inflammatory disorders or autoimmunity.

*DLG5* is the gene coding for an important scaffolding protein in maintaining epithelial structure and colonic barrier function. Since the original report identified *DLG5* as a candidate gene for IBD risk^24^, this association has been replicated in some studies but not all. Well powered studies in various populations failed to find any disease association^25^,^26^. In the genome wide meta-analysis of IBD patients, *DLG5* did not achieve genome-wide significance^7^. Our study found that *DLG5* variant (rs1248696) was not correlated with paediatric IBD. Despite the inability to replicate this association, we cannot rule out the possibility that *DLG5* plays a role in IBD pathogenesis in specific populations. The varied association of *DLG5* gene may be explained by genetic heterogeneity and a moderate effect. It is important to emphasize that, as a weak factor, a disease-causative effect could be easily modified in either a positive or negative way by gene-environment interactions. Therefore, even in the same population with the application of identical diagnostic criteria, variation in environmental exposures could lead to differences in genetic association.

The polymorphic rs2836878 variant is located on chromosome 21q22, a region of linkage disequilibrium that harbours no genes but is close to the *PSMG1* gene. This variant was identified as linked to early onset IBD in both paediatric IBD GWAS^8^,^9^. These observations interpreted as indicating that *PSMG1* is a paediatric-specific IBD related gene. However, association with *PSMG1* was later detected in a large scale meta-analysis of adult-onset GWAS^7^. Although the association of *PSMG1* gene was confirmed in both paediatric and adult-onset GWAS, the candidate gene studies presented inconsistent results. Four candidate gene studies were conducted to verify the role of *PSMG1* gene in paediatric IBD^27^,^28^,^29^,^30^. First, a Canadian study, which was excluded from the present analysis because it did not meet the age cut-off of paediatric IBD, observed a suggestion of association with the rs2836878 variant but this was not statistically significant^27^. Among the other three studies, two reported significant associations^29^,^30^, but the third, a larger study found no association^28^. After synthesizing data from these 3 studies, the association between rs2836878 and paediatric IBD was not statistically significant. Considering that small sample size and large genetic heterogeneity among populations may be responsible for the observed discrepancy, a larger study including functional data on *PSMG1* is required to elucidate the role of this gene in the pathogenesis of IBD.

The *TNF-α* gene has been identified as an IBD-susceptibility locus in genome wide scans^7^. Rs1800629 and rs1799724, two polymorphic variants of the *TNF-α* gene, have been studied extensively. Conflicting findings were observed in the associations between these polymorphisms and IBD development. A meta-analysis of genetic association studies of rs1800629 polymorphism in adulthood CD suggested no association^31^. Further phenotypic analysis reported that the rs1800629 polymorphism was more frequent in patients with steroid-dependent, colonic involvement and fistulising behaviour^32^. Our study extended consideration of the possible role of rs1800629 in paediatric CD by synthesizing data from five independent studies, and found a highly-credible association. This finding was consistent with the result of phenotype analysis reporting that colonic involvement and fistulising behaviour are more common in paediatric CD. For rs1799724, an association with adult CD has been reported and replicated in several studies, but no association was observed in paediatric patients^33^.

An association between the *PTPN2* gene and IBD has been confirmed in GWAS among adults^34^. Our study synthesized data on the rs2542151 polymorphism in *PTPN2* gene and paediatric IBD, and found no association, which is consistent with the findings of paediatric GWAS^8^,^9^. The explanation for the difference effect of rs2542151 polymorphism on adults and children is not yet clear, but phenotypic analysis indicated that the *PTPN2* gene had a role in patients with older age-at-diagnosis or who were smokers^35^.

The identified epigenetic studies found differential DNA methylation in IBD susceptibility loci involved in the immune response^36^. Methylation changes in *NOD2*, which in the present study is classified as highly-credibly associated with paediatric IBD, have been reported in whole blood samples of IBD patients^36^. *NOD2* has been reported to regulate the expression of miRNA-29 to reduce the release of IL-23, which indirectly influences the Th17 pathway in human dendritic cells^37^. Smoking is a known environmental modifier of epigenetic state and *NOD2* activation^38^. In relation to paediatric IBD, relevant routes of exposure may include maternal active and passive smoking during pregnancy and/or second-hand smoking exposure in childhood. The rs1800629 variant of *TNF-α*, identified as associated with paediatric CD with high credibility, is located in a transcription factor AP2 binding site, which is sensitive to methylation^39^. As the functional role of rs1800629 on TNF-α transcription is not clear, DNA methylation changes are perhaps important.

Two systematic reviews have been conducted to summarize observed associations between differential methylated DNA and dysregulated miRNA expression and adult IBD^10^,^40^. Comparing our results with these findings, differences in DNA methylation and miRNA expression patterns between child and adult IBD patients are apparent. To our knowledge, among the highlighted differential methylated genes identified in paediatric IBD, only *CFI*, *TNF-α* and *STAT3* have been replicated in adults, but not in the same tissues^10^,^40^. For the 26 dysregulated miRNA identified in paediatric IBD, only 8 were overlapped with those identified in adults^10^. In addition, particular note should be given to let-7 and miRNA-124, which have been identified in two paediatric studies^41^,^42^. It has been reported that both let-7 and miRNA-124 are involved in the inflammatory circuits leading to cell transformation through the activation of the STAT3 pathway, but miRNA-124 is specific to active paediatric UC^41^. Together with the findings of this field synopsis of genetic association studies, in which paediatric and adult-onset IBD share the majority of candidate genes, it is possible that epigenetic changes may be one of the reasons leading to the early disease onset and the distinct phenotypic features of paediatric IBD. The identified changes in DNA methylation and miRNA expression highlight the new aspects of disease pathogenesis and will be helpful to identify new clinical biomarkers and therapeutic targets.

In spite of the systematic internationally recognised approaches applied in this study, there is still room for future improvement in assessing the credibility of association. Venice criteria and BFDP improve the consistency and objectiveness of the interpretation and reporting of genetic associations, but this evidence does not prove causality. Additional evidence, such as gene knock-out experiments, gene expression microarray experiments or other mechanistic data are necessary to understand the specific functions of variants or genes. Associations with high credibility deserve in-depth evaluation including biological investigations; associations with moderate credibility indicate more genetic and biological studies are needed; weakly genetic associations may be not worthy of further investigation unless strong mechanistic evidence has been demonstrated.

One of the potential obstacles to distinguish the genetic susceptibility of paediatric IBD from adult-onset disease is the difficulty of recruiting affected children in sufficient numbers to have adequate statistical power to detect and characterize association. GWAS have shown that odds ratios of genome wide significant genetic variants in IBD are small and thus sample sizes need to be very large to identify variants specific to paediatric IBD with moderate or small effect sizes^7^,^8^,^9^. Given the limited sample sizes in the meta-analyses of paediatric IBD, this field synopsis is likely to be subject to false negatives. To overcome this problem, international collaboration is required in the future study to enlarge the sample size and multiple controls per case would increase power.

For epigenetic studies, there is widespread concern about the gene-set analysis approach generally applied in genome-wide methylation assays, which is severely biased as a result of differences in the numbers of CpG sites associated with different classes of genes and gene promoters^43^. New analytical methods developed for epigenomic analysis are required to avoid bias. Epigenetic research in paediatric IBD is just in its infancy; the heterogeneity among studies precludes a robust interpretation of results. Further well-designed studies are needed to clarify the role of epigenetic mechanisms in paediatric IBD pathogenesis.

In summary, our study supports the highly credible associations of 9 genetic variants with paediatric IBD, of which 4 variants had not been previously identified in paediatric GWAS. SNPs identified for paediatric IBD have been found also to be implicated in adult IBD and there is no evidence that genetic effects are of greater magnitude in children. Differential DNA methylation in *NOD2* and *TNF-α*, dysregulated expression in let-7 and miR-124 were putatively associated with paediatric IBD, and worthy of further investigation. There is the possibility that genetic variations outside the investigated genes and unexplored genetic-environment interactions may modulate the early age of disease onset. Further progress identifying genetic susceptibility loci and epigenetic changes specific to paediatric IBD will require international collaboration to increase statistical power, population diversity, and for epigenetic changes, harmonization of protocols for investigation and an open-ness to share data.

## Additional Information

**How to cite this article**: Li, X. *et al*. Systematic meta-analyses and field synopsis of genetic and epigenetic studies in paediatric inflammatory bowel disease. *Sci. Rep*. **6**, 34076; doi: 10.1038/srep34076 (2016).

## Supplementary Material

Supplementary Information

## Figures and Tables

**Table 1:** List of genes and variants selected for meta-analyses of paediatric CD and UC (sorted by gene).

Gene/Variant	Cases/controls	No. of studies	Ref allele	Ref allele frequency cases	Ref allelefrequency controls	MAF (1000 Genomes)	Result of most recent meta-analysis in adult-onset IBD; cases/controls (studies); (reference)	Result of meta-analysis in paediatric IBD; cases/controls (studies); (reference)
**Paediatric CD**								
***NOD2***								
rs2066844	1984/4179	16	C	0.912	0.958	0.014	Pos assoc; 17666/15491(72)^44^	n/a
rs2066845	2059/4280	16	G	0.941	0.976	0.005	Pos assoc; 17651/15908(72)^44^	n/a
rs2066847	1980/4234	16	G	0.872	0.976	0.006	Pos assoc; 18727/17102(79)^44^	n/a
rs5743289^†^	376/507	3	C	n/a	n/a	0.052	n/a	n/a
***IL23R***								
rs11209026	1158/4766	10	G	0.977	0.923	0.023	Pos assoc; 8110/11900(33)^22^	Pos assoc; 818/1958(5)^22^
rs7517847	496/1383	4	T	0.654	0.588	0.356	Pos assoc; 3279/4136(11)^15^	n/a
***IBD5***								
rs11739135	536/722	4	G	0.548	0.592	0.135	Pos assoc; 2295/2348(9)^16^	n/a
rs12521868	536/742	4	G	0.525	0.596	0.138	Pos assoc; 2788/3085(10)^16^	n/a
rs17622208	527/872	3	G	0.673	0.681	0.190	Pos assoc; 1686/1975(7)^16^	n/a
rs1050152	757/1474	7	C	0.528	0.577	0.134	Pos assoc; 4489/5351(15)^17^	Pos assoc; 485/922(5)^16^
rs26313667	653/1117	5	G	0.462	0.523	0.267	Pos assoc; 4474/5377(15)^17^	Pos assoc; 472/910(5)^16^
***ATG16L1***								
rs2241880	1360/6385	12	G	0.594	0.543	0.396	Pos assoc; 12762/16735(35)^22^	Pos assoc;1135/2457(6)^22^
***DLG5***								
rs1248696	427/846	4	C	0.919	0.898	0.034	No assoc; n/a (12)^45^	n/a
rs2289311	165/750	3	G	0.700	0.646	0.262	n/a	n/a
***PSMG1***								
rs2836878	425/705	3	G	0.746	0.748	0.209	n/a	n/a
***TNF-α***								
rs1800629	509/1443	6	G	0.830	0.882	0.090	No assoc; 3201/5033(20)^46^	n/a
rs1799724	326/854	3	C	0.842	0.870	0.099	No assoc; 2291/3796(11)^46^	n/a
***PTPN2***								
rs2542151	461/2152	4	T	0.858	0.862	0.174	Pos assoc; 9804/10642(13)^47^	n/a
***TLR4***								
rs4986790^†^	214/369	3	A	n/a	n/a	0.060	Pos assoc; n/a (33)^48^	n/a
***BSN-MST1***								
rs9858542^†^	414/679	3	G	n/a	n/a	0.195	n/a	n/a
**Paediatric UC**								
***NOD2***								
rs2066844	606/1512	6	C	0.94	0.96	0.014	No assoc; 6240/7350(32)^44^	n/a
rs2066845	591/1515	6	G	0.98	0.98	0.005	No assoc; 6016/6840(29)^44^	n/a
rs2066847	576/1240	6	G	0.97	0.98	0.006	No assoc; 6403/7875(35)^44^	n/a
***IL23R***								n/a
rs11209026	358/1344	3	G	0.96	0.94	0.023	Pos assoc; 5438/7380(16)^49^	n/a
***IBD5***								
rs1050152	392/1056	4	C	0.53	0.57	0.134	Pos assoc; 2923/4660(12)^16^	No assoc; 279/800(3)^16^
rs26313667	392/1044	4	G	0.48	0.52	0.267	Pos assoc; 2911/4635(12)^16^	No assoc; 279/788(3)^16^
***DLG5***								
rs1248696	318/800	3	C	0.92	0.90	0.034	n/a	n/a
***TNF-α***								
rs1800629	235/844	3	G	0.87	0.88	0.090	No assoc; 2423/4367(14)^50^	n/a

^†^For variants with genotype data reported as risk allele carrier (wt/var & var/var vs. wt/wt), reference allele frequency is unable to be calculated and meta-analyses were performed only in dominant model.

**Table 2:** Summary crude odds ratios (ORs) and 95% confidence intervals (95% CI) for dominant and recessive models for variants that were identified for meta-analysis with credibility factors in paediatric CD.

Gene/ Variant	Cases vs. controls (number of samples)	DOMINANT MODEL: wt/var & var/var VS. wt/wt							RECESSIVE MODEL: var/var vs. wt/wt & wt/var						
		N	Effect size		Heterogeneity		Credibility		N	Effect size		Heterogeneity		Credibility	
			OR (95% CI)	*P* value	I^2 (95% CI)	Power	BFDP^1^	Venice criteria grade		OR (95% CI)	*P* value	I^2 (95% CI)	Power	BFDP^1^	Venice criteria grade
*NOD2*/rs2066844	1984 vs. 4179 (16)	16	2.23 (1.88, 2.64)	4.77E-20	46 (2, 82)	1.00	0.000	ABB	12	4.14 (2.18, 7.89)	1.54E-05	0 (0, 49)	0.99	0.035	AAB
*NOD2/*rs2066845	2059 vs. 4274 (16)	16	3.21 (2.25, 4.60)	1.61E-10	53 (10, 84)	1.00	0.000	ACB	11	8.64 (2.80, 26.69)	1.77E-04	0 (0, 0)	1.00	0.465	AAB
*NOD2/*rs2066847	1980 vs. 4234 (16)	16	5.78 (3.67, 9.11)	4.12E-14	77 (58, 92)	1.00	0.000	ACB	10	91.00 (25.07, 330.27)	6.97E-12	0 (0, 57)	1.00	0.000	AAB
*NOD2/*rs5743289	376 vs. 687 (3)	3	1.48 (1.13, 1.94)	0.004	0 (0, 0)	0.83	0.640	AAB	n/a	n/a	n/a	n/a	n/a	n/a	n/a
*IL23R/* rs11209026	1158 vs. 4766 (10)	10	0.32 (0.23, 0.43)	2.10E-13	0 (0, 50)	1.00	0.000	AAB	7	0.97 (0.31, 3.04)	0.952	0 (0, 32)	0.05	0.963	CAB
*IL23R/*rs7517847	496 vs. 1383 (4)	4	0.66 (0.53, 0.83)	3.13E-04	0 (0, 79)	0.95	0.180	AAB	4	0.57 (0.41, 0.79)	8.97E-04	49 (0, 94)	0.96	0.262	ABB
*IBD5/*rs11739135	536 vs. 722 (4)	4	1.24 (0.97, 1.59)	0.082	48 (0, 98)	0.41	0.956	CBB	4	1.41 (1.05, 1.89)	0.024	28 (0, 98)	0.63	0.862	BBB
*IBD5/*rs12521868	536 vs. 731 (4)	4	1.35 (1.05, 1.73)	0.018	0 (0, 95)	0.68	0.856	BAB	4	1.76 (1.31, 2.35)	1.55E-04	0 (0, 23)	0.97	0.072	AAB
*IBD5/*rs17622208	527 vs. 872 (3)	3	1.38 (1.05, 1.81)	0.020	51 (0, 92)	0.83	0.861	ACB	2	1.28 (0.92, 1.78)	0.140	0 (0, 0)	0.33	0.961	CAB
*IBD5/*rs1050152	757 vs. 1534 (7)	7	1.20 (0.98, 1.47)	0.072	0 (0, 76)	0.45	0.959	CAB	7	1.48 (1.17, 1.86)	9.91E-04	0 (0, 79)	0.93	0.293	AAB
*IBD5/*rs26313667	653 vs. 1117 (5)	5	1.40 (1.11, 1.77)	0.005	0 (0, 83)	0.82	0.675	AAB	5	1.42 (1.13, 1.78)	0.003	0 (0, 7)	0.87	0.527	AAB
*ATG16L1/*rs2241880	1360 vs. 6385 (12)	12	0.70 (0.62, 0.81)	3.14E-07	0 (0, 53)	1.00	0.002	AAB	12	0.73 (0.62, 0.87)	2.70E-04	0 (0, 51)	0.90	0.225	ACB
*DLG5/*rs1248696	427 vs. 846 (4)	4	0.79 (0.57, 1.09)	0.148	0 (0, 94)	0.34	0.961	CAB	4	0.56 (0.14, 2.26)	0.416	0 (0, 81)	0.12	0.955	CAB
*DLG5/*rs2289311	165 vs. 750 (3)	3	0.76 (0.53, 1.10)	0.151	19 (0, 98)	0.36	0.958	CAB	3	0.55 (0.29, 1.06)	0.075	0 (0, 97)	0.50	0.920	BAB
*PSMG1/*rs2836878	425 vs. 705 (3)	3	1.06 (0.83, 1.36)	0.623	43 (0, 98)	0.07	0.987	CBB	3	0.98 (0.33, 2.91)	0.971	67 (0, 99)	0.05	0.964	CCB
*TNF-α/*rs1800629	509 vs. 1443 (5)	5	1.98 (1.29, 3.03)	0.002	57 (0, 96)	1.00	0.415	ACB	4	3.79 (1.96, 7.32)	7.52E-05	0 (0, 95)	0.98	0.101	AAB
*TNF-α/*rs1799724	326 vs. 854 (3)	3	0.90 (0.67, 1.22)	0.501	47 (0, 99)	0.10	0.983	CBB	3	0.63 (0.28, 1.41)	0.259	0 (0, 95)	0.13	0.955	CAB
*PTPN2/*rs2542151	461 vs. 2152 (3)	3	1.05 (0.83, 1.33)	0.667	0 (0, 82)	0.07	0.988	CAB	3	1.77 (0.88, 3.55)	0.108	0 (0, 95)	0.42	0.934	CAB
TLR4/rs4986790	214 vs. 369 (3)	3	1.54 (0.92, 2.58)	0.100	20 (0, 97)	0.38	0.937	CAB	n/a	n/a	n/a	n/a	n/a	n/a	n/a
BSN-MST1/rs9858542	414 vs. 679 (3)	3	2.08 (1.62, 2.68)	1.00E-05	13 (0, 83)	1.00	0.030	AAB	n/a	n/a	n/a	n/a	n/a	n/a	n/a

Venice criteria grade for the three criteria. The first grade is for the amount of evidence assessed according to statistical power (A, ≥80%; B, 50–79%; C, <50%); the second grade is for the extent of replication assessed according to heterogeneity (I^2^ value: A, <25%; B, 25–50%; C, >50%); the third grade is for protection from bias assessed according to small study effect (complete assessment of bias is difficult; no variants were graded as “A”; “B” was assigned for studies which no small study effect was detected; otherwise, “C” was assigned). Bayesian False Discovery Probability (BFDP) value were calculated at prior probability of 0.05. BFDP level of noteworthiness is 0.2.

**Table 3:** Summary crude odds ratios (ORs) and 95% confidence intervals (95% CI) for two additive models for variants that were identified for meta-analysis with credibility factors in paediatric CD.

Gene/Variant	Cases vs. controls (number of samples)	ADDITIVE MODEL 1: var/wt VS. wt/wt							ADDITIVE MODEL 2: var/var VS. wt/wt						
		N	Effect size		Heterogeneity		Credibility		N	Effect size		Heterogeneity		Credibility	
			OR (95% CI)	*P* value	I^2 (95% CI)	Power	BFDP^1^	Venice criteria grade		OR (95% CI)	*P* value	I^2 (95% CI)	Power	BFDP^1^	Venice criteria grade
*NOD2*/rs2066844	1984 vs. 4179 (16)	16	2.11 (1.77, 2.52)	9.81E-17	36 (0, 78)	1.00	0.000	ABB	12	4.44 (2.34, 8.43)	5.26E-06	0 (0, 54)	0.99	0.016	AAB
*NOD2/*rs2066845	2059 vs. 4274 (16)	16	3.02 (2.42, 3.76)	5.04E-23	50 (6, 82)	1.00	0.000	ABB	11	9.18 (2.99, 28.22)	1.09E-04	0 (0, 0)	1.00	0.405	AAB
*NOD2/*rs2066847	1980 vs. 4234 (16)	16	5.03 (3.34, 7.59)	1.34E-14	70 (46, 89)	1.00	0.000	ACB	10	105.94 (30.65, 366.14)	1.72E-13	0 (0, 67)	1.00	0.000	AAB
*IL23R/* rs11209026	1158 vs. 4766 (10)	10	0.33 (0.24, 0.44)	8.29E-13	0 (0, 51)	1.00	0.000	AAB	7	0.88 (0.29, 2.76)	0.823	0 (0, 30)	0.06	0.963	CAB
*IL23R/*rs7517847	496 vs. 1383 (4)	4	0.73 (0.58, 0.93)	0.010	0 (0, 41)	0.80	0.800	AAB	4	0.47 (0.32, 0.67)	4.11E-05	45 (0, 93)	1.00	0.023	ABB
*IBD5/*rs11739135	536 vs. 722 (4)	4	1.17 (0.90, 1.51)	0.245	15 (0, 97)	0.23	0.977	CAB	4	1.55 (1.11, 2.17)	0.011	56 (0, 99)	0.73	0.767	BCB
*IBD5/*rs12521868	536 vs. 731 (4)	4	1.20 (0.92, 1.55)	0.178	0 (0, 94)	0.28	0.971	CAB	4	1.88 (1.34, 2.62)	2.22E-04	0 (0, 96)	0.96	0.101	AAB
*IBD5/*rs17622208	527 vs. 872 (3)	3	1.33 (1.00, 1.78)	0.050	53 (0, 92)	0.54	0.930	BCB	2	1.61 (1.09, 2.37)	0.020	34 (0, 85)	0.64	0.811	BBB
*IBD5/*rs1050152	757 vs. 1534 (7)	7	1.09 (0.88, 1.35)	0.416	0 (0, 76)	0.13	0.987	CAB	7	1.55 (1.18, 2.02)	0.001	0 (0, 79)	0.92	0.360	AAB
*IBD5/*rs26313667	653 vs. 1117 (5)	5	1.28 (1.00, 1.64)	0.051	0 (0, 84)	0.51	0.934	BAB	5	1.68 (1.26, 2.23)	3.67E-04	0 (0, 67)	0.96	0.153	AAB
*ATG16L1/*rs2241880	1360 vs. 6385 (12)	12	0.74 (0.64, 0.86)	4.14E-05	0 (0, 33)	0.98	0.067	AAB	12	0.61 (0.51, 0.74)	2.85E-07	4 (0, 60)	1.00	0.001	AAB
*DLG5/*rs1248696	427 vs. 846 (4)	4	0.80 (0.57, 1.12)	0.192	0 (0, 93)	0.27	0.968	CAB	4	0.55 (0.14, 2.21)	0.397	0 (0, 82)	0.13	0.955	CAB
*DLG5/*rs2289311	165 vs. 750 (3)	3	0.84 (0.57, 1.23)	0.371	4 (0, 98)	0.16	0.976	CAB	3	0.51 (0.26, 1.01)	0.053	0 (0, 97)	0.58	0.954	BAB
*PSMG1/*rs2836878	425 vs. 705 (3)	3	1.13 (0.88, 1.46)	0.339	0 (0, 96)	0.16	0.982	CAB	3	1.10 (0.33, 3.72)	0.872	72 (0, 99)	0.07	0.962	CCB
*TNF-α/*rs1800629	509 vs. 1443 (5)	5	1.91 (1.27, 2.85)	0.002	51 (0, 95)	1.00	0.397	ACB	4	4.75 (2.43, 9.26)	4.86E-06	0 (0, 96)	1.00	0.017	AAB
*TNF-α/*rs1799724	326 vs. 854 (3)	3	0.94 (0.69, 1.29)	0.718	47 (0, 99)	0.07	0.985	CBB	3	0.60 (0.26, 1.36)	0.221	0 (0, 96)	0.15	0.952	CAB
*PTPN2/*rs2542151	461 vs. 2152 (3)	3	1.01 (0.79, 1.28)	0.955	0 (0, 0)	0.05	0.989	CAB	3	1.77 (0.88, 3.56)	0.108	0 (0, 95)	0.42	0.934	CAB

Venice criteria grade for the three criteria. The first grade is for the amount of evidence assessed according to statistical power (A, ≥80%; B, 50–79%; C, <50%); the second grade is for the extent of replication assessed according to heterogeneity (I^2^ value: A, <25%; B, 25–50%; C, >50%); the third grade is for protection from bias assessed according to small study effect (complete assessment of bias is difficult; no variants were graded as “A”; “B” was assigned for studies which no small study effect was detected; otherwise, “C” was assigned). Bayesian False Discovery Probability (BFDP) value were calculated at prior probability of 0.05. BFDP level of noteworthiness is 0.2.

**Table 4:** Effect sizes and *p*-values from paediatric and adult GWAS studies of the identified highly-credible variants.

Highly-credible variants	Field synopsis of paediatric IBD		GWAS in paediatric IBD			GWAS in adult IBD		
	p-value	OR (95%CI)	Genome-wide significance	p-value	OR (95%CI)†	Genome-wide significance	p-value	OR (95%CI)
***NOD2***								
rs2066844	9.81E-17	2.11 (1.77, 2.52)	Yes	7.44E-09^51^	2.06(1.61.2.64)	Yes	n/a^34^	n/a
rs2066845	5.04E-23	3.02 (2.42, 3.76)	Yes	3.00E-07^51^	2.58(1.80, 3.70)	Yes	4.62E-08^34^	n/a
rs2066847	1.34E-14	5.03 (3.34, 7.59)	n/a	n/a	n/a	Yes	2.98E-24^34^; 5.86E-209^7^	3.99 (n/a, n/a)
***IL23R***								
rs11209026	8.29E-13	0.33 (0.24, 0.44)	Yes	3.35E-10^8^	0.28(0.18, 0.43)	Yes	2.15E-68^34^	n/a
rs7517847	0.01	0.73 (0.58, 0.93)	Yes	8.22E-08^51^	0.70(0.61, 0.79)	Yes	3.06E-12^52^	n/a
***IBD5***								
rs12521868	2.22E-04	1.88 (1.34, 2.62)	No	0.002^51^	1.22(1.07, 1.39)	Yes	1.41E-20^34^	n/a
rs26313667	3.67E-04	1.68 (1.26, 2.23)	n/a	n/a	n/a	n/a	n/a	n/a
***ATG16L1***								
rs2241880	4.14E-05	0.74 (0.64, 0.86)	Yes	7.63E-10^8^; 1.09E-18^9^	0.69(0.61, 0.78)	Yes	4.61E-25^34^; 4.10E-08^52^	0.68 (n/a, n/a)
***TNF-α***								
rs1800629	4.86E-06	4.75 (2.43, 9.26)	n/a	n/a	n/a	n/a	n/a	n/a

Genome-wide significance in GWAS was checked in GWAS catalog: https://www.ebi.ac.uk/gwas/. P-value and OR (95%CI) reported in GWAS were estimated for minor allele code; P-value and OR (95%CI) reported in field synopsis were estimated for two additive models (*var/wt vs. wt/wt* or *var/var vs. wt/wt*).

**Table 5:** Summary crude odds ratios (ORs) and 95% confidence intervals (95% CI) for recessive and dominant models for variants that were identified for meta-analysis with credibility factors in paediatric UC.

Gene/ Variant	Cases vs. controls (number of samples)	DOMINANT MODEL: wt/var & var/var VS. wt/wt							RECESSIVE MODEL: var/var vs. wt/wt & wt/var						
		N	Effect size		Heterogeneity		Credibility		N	Effect size		Heterogeneity		Credibility	
			OR (95% CI)	*P* value	I^2 (95% CI)	Power	BFDP^1^	Venice criteria grade		OR (95% CI)	*P* value	I^2 (95% CI)	Power	BFDP^1^	Venice criteria grade
NOD2/rs2066844	590 vs. 1512 (6)	6	1.66 (1.20, 2.28)	0.002	34 (0, 91)	0.88	0.427	ABB	4	0.96 (0.20, 4.68)	0.960	0 (0, 77)	0.05	0.959	CAB
NOD2/rs2066845	591 vs. 1515 (6)	6	1.14 (0.70, 1.87)	0.600	0 (0, 87)	0.09	0.976	CAB	2	3.24 (0.17, 63.38)	0.439	0 (0, 0)	0.30	0.951	CAB
NOD2/rs2066847	593 vs. 1502 (6)	6	1.76 (1.11, 2.80)	0.010	0 (0, 95)	0.74	0.813	BAB	n/a	n/a	n/a	n/a	n/a	n/a	n/a
IL23R/ rs11209026	393 vs. 1321 (3)	3	0.55 (0.37, 0.84)	0.005	0 (0, 83)	0.85	0.653	AAB	n/a	n/a	n/a	n/a	n/a	n/a	n/a
IBD5/rs1050152	353 vs. 1056 (4)	4	1.22 (0.93, 1.61)	0.150	0 (0, 82)	0.31	0.968	CAB	4	1.40 (1.03, 1.91)	0.030	0 (0, 90)	0.61	0.897	BAB
IBD5/rs26313667	353 vs. 1044 (4)	4	1.30 (0.97, 1.75)	0.081	0 (0, 66)	0.46	0.947	CAB	4	1.29 (0.97, 1.71)	0.085	17 (0, 98)	0.44	0.946	CAB
DLG5/rs1248696	289 vs. 800 (3)	3	0.85 (0.58, 1.24)	0.405	0 (0, 75)	0.14	0.970	CAB	3	0.74 (0.16, 3.33)	0.690	0 (0, 97)	0.06	0.958	CAB
TNF-α/rs1800629	235 vs. 844 (3)	3	1.81 (0.82, 3.99)	0.143	69 (0, 99)	0.95	0.941	ACB	3	3.08 (0.99, 9.55)	0.052	0 (0, 97)	0.74	0.913	BAB

Venice criteria grade for the three criteria. The first grade is for the amount of evidence assessed according to statistical power (A, ≥80%; B, 50–79%; C, <50%); the second grade is for the extent of replication assessed according to heterogeneity (I^2^ value: A, <25%; B, 25–50%; C, >50%); the third grade is for protection from bias assessed according to small study effect (complete assessment of bias is difficult; no variants were graded as “A”; “B” was assigned for studies which no small study effect was detected; otherwise, “C” was assigned). Bayesian False Discovery Probability (BFDP) value were calculated at prior probability of 0.05. BFDP level of noteworthiness is 0.2.

**Table 6:** Summary crude odds ratios (ORs) and 95% confidence intervals (95% CI) for two additive models for variants that were identified for meta-analysis with credibility factors in paediatric UC.

Gene/Variant	Cases vs. controls (number of samples)	ADDITIVE MODEL 1: var/wt VS. wt/wt							ADDITIVE MODEL 2: var/var VS. wt/wt						
		N	Effect size		Heterogeneity		Credibility		N	Effect size		Heterogeneity		Credibility	
			OR (95% CI)	*P* value	I^2 (95% CI)	Power	BFDP^1^	Venice criteria grade		OR (95% CI)	*P* value	I^2 (95% CI)	Power	BFDP^1^	Venice criteria grade
*NOD2/*rs2066844	590 vs. 1512 (6)	6	1.73 (1.25, 2.36)	8.80E-04	34 (0, 91)	0.88	0.215	ABB	4	1.02 (0.21, 5.00)	0.980	0 (0, 79)	0.05	0.959	CAB
*NOD2/*rs2066845	591 vs. 1515 (6)	6	1.11 (0.68, 1.84)	0.600	0 (0, 87)	0.09	0.976	CAB	2	3.06 (0.16, 57.43)	0.455	0 (0, 0)	0.28	0.951	CAB
*NOD2/*rs2066847	593 vs. 1502 (6)	6	1.79 (1.13, 2.84)	0.010	0 (0, 0)	0.74	0.785	BAB	n/a	n/a	n/a	n/a	n/a	n/a	n/a
*IL23R/* rs11209026	393 vs. 1321(3)	3	0.56 (0.37, 0.85)	0.007	0 (0, 83)	0.84	0.677	AAB	n/a	n/a	n/a	n/a	n/a	n/a	n/a
*IBD5/*rs1050152	353 vs. 1056 (4)	4	1.12 (0.84, 1.50)	0.430	0 (0, 76)	0.12	0.983	CAB	4	1.52 (1.06, 2.17)	0.022	0 (0, 90)	0.66	0.848	BAB
*IBD5/*rs26313667	353 vs. 1044 (4)	4	1.22 (0.89, 1.67)	0.209	0 (0, 91)	0.25	0.971	CAB	4	1.49 (1.04, 2.13)	0.031	0 (0, 87)	0.61	0.876	BAB
*DLG5/*rs1248696	289 vs. 800 (3)	3	0.86 (0.58, 1.27)	0.450	0 (0, 62)	0.14	0.978	CAB	3	0.72 (0.16, 3.24)	0.664	0 (0, 97)	0.06	0.958	CAB
*TNF-α/*rs1800629	235 vs. 844 (3)	3	1.65 (1.10, 2.47)	0.015	61 (0, 99)	0.83	0.802	ACB	3	3.34 (1.09, 10.22)	0.035	0 (0, 98)	0.80	0.889	AAB

Venice criteria grade for the three criteria. The first grade is for the amount of evidence assessed according to statistical power (A, ≥80%; B, 50–79%; C, <50%); the second grade is for the extent of replication assessed according to heterogeneity (I^2^ value: A, <25%; B, 25–50%; C, >50%); the third grade is for protection from bias assessed according to small study effect (complete assessment of bias is difficult; no variants were graded as “A”; “B” was assigned for studies which no small study effect was detected; otherwise, “C” was assigned). Bayesian False Discovery Probability (BFDP) value were calculated at prior probability of 0.05. BFDP level of noteworthiness is 0.2.

**Table 7:** Highlighted differential methylated genes and dysregulated miRNAs in paediatric IBD.

Samples	Highlighted loci with differential DNA methylation	Dysregulated miRNA expression
Peripheral blood leukocyte	VMP1, RPS6KA2, ARHGEF3, CFI*, PLCH1, HK2, TNFSF10, SOCS3, ZBTB16, ZEB2, INPP4B, GSDMC, WASF2, MYO1E, CLU, SBNO2, NRXN2, SLC25A13, IL18RAP, LOXL2, CX3CR1, RTP5, RFTN1, PTGER4, CDC42BPB, RUNX3, POLK, NOD2, GPRIN3, HEATR2, MPRIP, SLC15A4, SLC10A6, MIR21, CNOT6L, NMRAL1, SYNJ2, CBFA2T2, PTDSS2, NLRC5, ZC3H4, PRKCE, FKBP5, TNF-α*, TRPS1, NPDC1, TBPL1, LAMA5, C6orf48, SNORD52, SLC6A9, FAM53B	**n/a**
Intestinal biopsies	IFITM1, ITGB2, S100A9, SLPI, SAA1, STAT3*	**miR-223**^**§**^, miR-1973, miR-3611, **miR-21**^**§**^, miR-3182, miR-877, **miR-146b**^**§**^, miR-3646, miR-3173, **let-7**^**§†**^, miR-892a, **miR-224**^**§**^, miR-24-1, **miR-424**^**§**^, miR-34a, **miR-124**^**†**^, miR-4323, **miR-378b**^**§**^, miR-3133, miR-4286, miR-138-1, miR-378a/c/d, miR-4284, miR-125, **miR-26**^**§**^, miR-101

Differential methylated genes highlighted with symbols (*) have been identified in adult-onset IBD but not in the same tissues. Dysregulated miRNAs highlighted with symbols (^§^) have been identified in adult-onset IBD in intestinal biopsies. Dysregulated miRNAs highlighted with symbols (^†^) have been replicated in two paediatric studies.

## References

[b1] Baumgart D. C. & Sandborn W. J. Inflammatory bowel disease: clinical aspects and established and evolving therapies. Lancet (London, England) 369, 1641–1657, doi: 10.1016/s0140-6736(07)60751-x (2007). 17499606

[b2] Cosnes J., Gower-Rousseau C., Seksik P. & Cortot A. Epidemiology and natural history of inflammatory bowel diseases. Gastroenterology 140, 1785–1794, doi: 10.1053/j.gastro.2011.01.055 (2011). 21530745

[b3] Griffiths A. M. Growth retardation in early-onset inflammatory bowel disease: should we monitor and treat these patients differently? Digestive diseases (Basel, Switzerland) 27, 404–411, doi: 10.1159/000228581 (2009). 19786772

[b4] Halme L. . Family and twin studies in inflammatory bowel disease. World journal of gastroenterology 12, 3668–3672 (2006). 16773682 10.3748/wjg.v12.i23.3668 PMC4087458

[b5] Orholm M. . Familial occurrence of inflammatory bowel disease. The New England journal of medicine 324, 84–88, doi: 10.1056/nejm199101103240203 (1991). 1984188

[b6] Orholm M., Binder V., Sorensen T. I., Rasmussen L. P. & Kyvik K. O. Concordance of inflammatory bowel disease among Danish twins. Results of a nationwide study. Scandinavian journal of gastroenterology 35, 1075–1081 (2000). 11099061 10.1080/003655200451207

[b7] Jostins L. . Host-microbe interactions have shaped the genetic architecture of inflammatory bowel disease. Nature 491, 119–124, doi: 10.1038/nature11582 (2012). 23128233 PMC3491803

[b8] Imielinski M. . Common variants at five new loci associated with early-onset inflammatory bowel disease. Nature genetics 41, 1335–1340, doi: 10.1038/ng.489 (2009). 19915574 PMC3267927

[b9] Kugathasan S. . Loci on 20q13 and 21q22 are associated with pediatric-onset inflammatory bowel disease. Nature genetics 40, 1211–1215, doi: 10.1038/ng.203 (2008). 18758464 PMC2770437

[b10] Ventham N. T., Kennedy N. A., Nimmo E. R. & Satsangi J. Beyond gene discovery in inflammatory bowel disease: the emerging role of epigenetics. Gastroenterology 145, 293–308, doi: 10.1053/j.gastro.2013.05.050 (2013). 23751777 PMC3919211

[b11] Roberts S. E., Wotton C. J., Williams J. G., Griffith M. & Goldacre M. J. Perinatal and early life risk factors for inflammatory bowel disease. World journal of gastroenterology 17, 743–749, doi: 10.3748/wjg.v17.i6.743 (2011). 21390144 PMC3042652

[b12] Ioannidis J. P. . Assessment of cumulative evidence on genetic associations: interim guidelines. International journal of epidemiology 37, 120–132, doi: 10.1093/ije/dym159 (2008). 17898028

[b13] Zintzaras E. & Lau J. Synthesis of genetic association studies for pertinent gene-disease associations requires appropriate methodological and statistical approaches. Journal of clinical epidemiology 61, 634–645, doi: 10.1016/j.jclinepi.2007.12.011 (2008). 18538260

[b14] Wakefield J. A Bayesian measure of the probability of false discovery in genetic epidemiology studies. American journal of human genetics 81, 208–227, doi: 10.1086/519024 (2007). 17668372 PMC1950810

[b15] Zhang L. . Interleukin-23R rs7517847 T/G Polymorphism Contributes to the Risk of Crohn’s Disease in Caucasians: A Meta-Analysis. Journal of immunology research (2015). 10.1155/2015/279849 PMC4451526 26090488

[b16] Wang J. . Contribution of the IBD5 locus to inflammatory bowel disease: A meta-analysis. Hum Genet 129, 597–609 (2011). 21279723 10.1007/s00439-011-0952-6

[b17] Xuan C. . Association between OCTN1/2 gene polymorphisms (1672C-T, 207G-C) and susceptibility of Crohn’s disease: a meta-analysis. Int J Colorectal Dis. 27, 11–19 (2012). 21706137 10.1007/s00384-011-1265-x

[b18] Economou M., Trikalinos T. A., Loizou K. T., Tsianos E. V. & Ioannidis J. P. A. Differential effects of NOD2 variants on Crohn’s disease risk and phenotype in diverse populations: A metaanalysis. The American journal of gastroenterology 99, 2393–2404 (2004). 15571588 10.1111/j.1572-0241.2004.40304.x

[b19] Duerr R. H. . A genome-wide association study identifies IL23R as an inflammatory bowel disease gene. Science (New York, N.Y.) 314, 1461–1463, doi: 10.1126/science.1135245 (2006). PMC4410764 17068223

[b20] Rioux J. D. . Genomewide search in Canadian families with inflammatory bowel disease reveals two novel susceptibility loci. American journal of human genetics 66, 1863–1870, doi: 10.1086/302913 (2000). 10777714 PMC1378042

[b21] Mirza M. M. . Genetic evidence for interaction of the 5q31 cytokine locus and the CARD15 gene in Crohn disease. American journal of human genetics 72, 1018–1022 (2003). 12618963 10.1086/373880 PMC1180331

[b22] Grigoras C. A., Ziakas P. D., Jayamani E. & Mylonakis E. ATG16L1 and IL23R variants and genetic susceptibility to crohn’s disease: mode of inheritance based on meta-analysis of genetic association studies. Inflamm Bowel Dis 21, 768–776 (2015). 25738374 10.1097/MIB.0000000000000305

[b23] Levine B. & Deretic V. Unveiling the roles of autophagy in innate and adaptive immunity. Nature Reviews Immunology 7, 767–777 (2007). 10.1038/nri2161 PMC7097190 17767194

[b24] Stoll M. . Genetic variation in DLG5 is associated with inflammatory bowel disease. Nature genetics 36, 476–480 (2004). 15107852 10.1038/ng1345

[b25] Noble C. L. . DLG5 variants do not influence susceptibility to inflammatory bowel disease in the Scottish population. Gut 54, 1416–1420, doi: 10.1136/gut.2005.066621 (2005). 15843420 PMC1774698

[b26] Yamazaki K. . Association analysis of SLC22A4, SLC22A5 and DLG5 in Japanese patients with Crohn disease. Journal of human genetics 49, 664–668, doi: 10.1007/s10038-004-0204-x (2004). 15503241

[b27] Amre D. K. . Investigation of reported associations between the 20q13 and 21q22 loci and pediatric-onset Crohn’s disease in Canadian children. The American journal of gastroenterology 104, 2824–2828 (2009). 19623168 10.1038/ajg.2009.430

[b28] Latiano A. . Investigation of multiple susceptibility loci for inflammatory bowel disease in an Italian cohort of patients. Plos One 6, e22688 (2011). 21818367 10.1371/journal.pone.0022688 PMC3144927

[b29] Wagner J. . Interaction of Crohn’s disease susceptibility genes in an Australian paediatric cohort. Plos one 5, e15376 (2010). 21079743 10.1371/journal.pone.0015376 PMC2975706

[b30] Wagner J. . TLR4, IL10RA, and NOD2 mutation in paediatric Crohn’s disease patients: an association with Mycobacterium avium subspecies paratuberculosis and TLR4 and IL10RA expression. Medical microbiology and immunology 202, 267–276 (2013). 23455702 10.1007/s00430-013-0290-5

[b31] Ferguson L. R. . Single nucleotide polymorphism in the tumor necrosis factor-alpha gene affects inflammatory bowel diseases risk. World journal of gastroenterology 14, 4652–4661 (2008). 18698679 10.3748/wjg.14.4652 PMC2738789

[b32] Louis E. . Tumour necrosis factor (TNF) gene polymorphism in Crohn’s disease (CD): influence on disease behaviour? Clinical and experimental immunology 119, 64–68 (2000). 10606965 10.1046/j.1365-2249.2000.01106.x PMC1905519

[b33] O’Callaghan N. J., Adams K. E., van Heel D. A. & Cavanaugh J. A. Association of TNF-alpha-857C with inflammatory bowel disease in the Australian population. Scandinavian journal of gastroenterology 38, 533–534 (2003). 12795465

[b34] Barrett J. C. . Genome-wide association defines more than 30 distinct susceptibility loci for Crohn’s disease. Nature genetics 40, 955–962, doi: 10.1038/ng.175 (2008). 18587394 PMC2574810

[b35] Morgan A. R. . PTPN2 but not PTPN22 is associated with Crohn’s disease in a New Zealand population. Tissue antigens 76, 119–125, doi: 10.1111/j.1399-0039.2010.01493.x (2010). 20403149

[b36] Nimmo E. R. . Genome-wide methylation profiling in Crohn’s disease identifies altered epigenetic regulation of key host defense mechanisms including the Th17 pathway. Inflammatory bowel diseases 18, 889–899, doi: 10.1002/ibd.21912 (2012). 22021194

[b37] Kalla R. . MicroRNAs: new players in IBD. Gut 64, 504–517, doi: 10.1136/gutjnl-2014-307891 (2015). 25475103 PMC4345829

[b38] Aldhous M. C. . Cigarette smoke extract (CSE) delays NOD2 expression and affects NOD2/RIPK2 interactions in intestinal epithelial cells. Plos one 6, e24715, doi: 10.1371/journal.pone.0024715 (2011). 21931826 PMC3171477

[b39] Petronis A. & Petroniene R. Epigenetics of inflammatory bowel disease. Gut 47, 302–306 (2000). 10896927 10.1136/gut.47.2.302 PMC1728011

[b40] Karatzas P. S., Gazouli M., Safioleas M. & Mantzaris G. J. DNA methylation changes in inflammatory bowel disease. Annals of gastroenterology : quarterly publication of the Hellenic Society of Gastroenterology 27, 125–132 (2014). 24733658 PMC3982627

[b41] Koukos G. . MicroRNA-124 regulates STAT3 expression and is down-regulated in colon tissues of pediatric patients with ulcerative colitis. Gastroenterology 145, 842-852 e842 (2013). 10.1053/j.gastro.2013.07.001 PMC4427058 23856509

[b42] Koukos G. . MicroRNA-4284 regulates CXCL5 expression and is down-regulated in colon tissues of pediatric patients with ulcerative colitis. Gastroenterology 1, S-781 (2015). 10.1053/j.gastro.2013.07.001 PMC4427058 23856509

[b43] Geeleher P. . Gene-set analysis is severely biased when applied to genome-wide methylation data. Bioinformatics (Oxford, England) 29, 1851–1857, doi: 10.1093/bioinformatics/btt311 (2013). 23732277

[b44] Yazdanyar S., Weischer M. & Nordestgaard B. G. Genotyping for NOD2 genetic variants and crohn disease: a metaanalysis. Clin Chem. 55, 1950–1957 (2009). 19713276 10.1373/clinchem.2009.127126

[b45] Browning B. L. . Association of DLG5 variants with inflammatory bowel disease in the New Zealand Caucasian population and meta-analysis of the DLG5 R30Q variant. Inflammatory bowel diseases 13, 1069–1076 (2007). 17455201 10.1002/ibd.20157

[b46] Xie C., Liu X. F. & Yang M. S. A meta-analysis on the association between three promoter variants of TNF-alpha and Crohn’s disease. Mol Biol Rep. 39, 1575–1583 (2012). 21633892 10.1007/s11033-011-0896-x

[b47] Zhang J. X. . Associations between PTPN2 polymorphisms and susceptibility to ulcerative colitis and Crohn’s disease: a meta-analysis. Inflamm Res. 63, 71–79 (2014). 24127071 10.1007/s00011-013-0673-5

[b48] Cheng Y. . Association between TLR2 and TLR4 gene polymorphisms and the susceptibility to inflammatory bowel disease: A meta-analysis. Plos one 10, e0126803 (2015). 26023918 10.1371/journal.pone.0126803 PMC4449210

[b49] Liu M. . Interleukin-23 receptor genetic polymorphisms and ulcerative colitis susceptibility: A meta-analysis. Clin Res Hepatol Gastroenterol 39, 516–525 (2015). 25497273 10.1016/j.clinre.2014.10.009

[b50] Fan W. . Relationship between the polymorphism of tumor necrosis factor-alpha-308 G > A and susceptibility to inflammatory bowel diseases and colorectal cancer: a meta-analysis. Eur J Hum Genet 19, 432–437 (2011). 21248737 10.1038/ejhg.2010.159 PMC3060311

[b51] Cutler D. J. . Dissecting Allele Architecture of Early Onset IBD Using High-Density Genotyping. PloS one 10, e0128074, doi: 10.1371/journal.pone.0128074 (2015). 26098103 PMC4476779

[b52] Rioux J. D. . Genome-wide association study identifies new susceptibility loci for Crohn disease and implicates autophagy in disease pathogenesis. Nature genetics 39, 596–604, doi: 10.1038/ng2032 (2007). 17435756 PMC2757939

