# Supplementary material for: Systematic meta-analyses and field synopsis of genetic and epigenetic studies in paediatric inflammatory bowel disease
Source: Sci Rep. 2016 Sep 27;6:34076. doi: 10.1038/srep34076 (PMC5037432; doi:10.1038/srep34076)
Supplement: Supplementary Information [file srep34076-s1.pdf]

SUPPLEMENTARY INFORMATION

TITLE Systematic meta-analyses and field synopsis of genetic and epigenetic studies in paediatric inflammatory bowel disease

AUTHORS Xue Li[1], Peige Song[1], Maria Timofeeva[2], Xiangrui Meng[1], Igor Rudan[1], Julian Little[3], Jack Satsangi[4], Harry Campbell[1]*, Evropi Theodoratou[1]*

1 Centre for Population Health Sciences, University of Edinburgh, Edinburgh, United Kingdom

2 Colon Cancer Genetics Group and Academic Coloproctology, Institute of Genetics and Molecular Medicine, University of Edinburgh and MRC Human Genetics Unit Western General Hospital Edinburgh, Edinburgh, United Kingdom

3 School of Epidemiology, Public Health and Preventive Medicine, University of Ottawa, Ottawa, Canada

4 Centre for Genomic and Experimental Medicine, Institute of Genetics and Molecular Medicine, University of Edinburgh and Western General Hospital Edinburgh, Edinburgh, United Kingdom

***Corresponding authors**

Evropi Theodoratou, Centre for Population Health Sciences, University of Edinburgh, Edinburgh, United Kingdom, EH8 9AG; E-mail: E.Theodoratou@ed.ac.uk, Tel: (+44) 131-650-6194

Harry Campbell, Centre for Population Health Sciences, University of Edinburgh, Edinburgh, United Kingdom, EH8 9AG; E-mail: Harry.Campbell@ed.ac.uk, Tel: (+44) 131-650-3218

Supplementary Tables S1-S9

Supplementary Table S1 Keywords and search strategy in literature review

Genetic association studies in paediatric IBD

MEDLINE (OvidSP)

1. Inflammatory bowel disease.mp. or inflammatory bowel disease/
2. Crohn disease.mp. or Crohn disease/
3. Ulcerative colitis.mp. or ulcerative colitis/
4. Colitis, Ulcerative/ or Inflammatory Bowel Diseases/ or IBD.mp. or Crohn Disease/
5. ileitis.mp. or ileitis/
6. pouchitis.mp. or ileitis/
7. proctitis.mp. or proctitis/
8. proctocolitis.mp. or proctocolitis/
9. enteritis.mp. or enteritis/
10. duodenitis.mp. or duodenitis/
11. 1 or 2 or 3 or 4 or 5 or 6 or 7 or 8 or 9 or 10
12. Genetic predisposition to disease.mp. or genetic predisposition/
13. (Gene$ and associat$).mp. [mp=title, abstract, subject headings, heading word, drug trade name, original title, device manufacturer, drug manufacturer, device trade name, keyword]
14. 13 or 14
15. 12 and 15
16. limit 16 to child

EMBASE (OvidSP)

1. Inflammatory bowel disease.mp. or inflammatory bowel disease/
2. Crohn disease.mp. or Crohn disease/
3. Ulcerative colitis.mp. or ulcerative colitis/
4. Colitis, Ulcerative/ or Inflammatory Bowel Diseases/ or IBD.mp. or Crohn Disease/
5. ileitis.mp. or ileitis/
6. pouchitis.mp. or ileitis/
7. proctitis.mp. or proctitis/
8. proctocolitis.mp. or proctocolitis/
9. enteritis.mp. or enteritis/
10. duodenitis.mp. or duodenitis/
11. 1 or 2 or 3 or 4 or 5 or 6 or 7 or 8 or 9 or 10
12. Genetic predisposition to disease.mp. or genetic predisposition/
13. (gene$ and associat$).mp. [mp=title, abstract, subject headings, heading word, drug trade name, original title, device manufacturer, drug manufacturer, device trade name, keyword]
14. 13 or 14
15. 12 and 15
16. limit 16 to child

Human Genome Epidemiology Network Navigator (version 2.0)

1. inflammatory bowel disease
2. Crohn disease
3. Colitis, Ulcerative
4. IBD
5. Ileitis
6. pouchitis
7. proctitis

8. proctocolitis
9. enteritis
10. duodenitis

Epigenetic studies in paediatric IBD

MEDLINE (OvidSP)

1. Inflammatory bowel disease.mp. or Inflammatory Bowel Diseases/
2. Crohn disease.mp. or Crohn Disease/
3. Ulcerative colitis.mp. or Colitis, Ulcerative/
4. Colitis, Ulcerative/ or Inflammatory Bowel Diseases/ or IBD.mp. or Crohn Disease/
5. 1 or 2 or 3 or 4
6. Methylation/ or DNA Methylation/ or methylation.mp.
7. Interaction$.mp. or Gene-Environment Interaction/
8. Epigenetic$.mp. or Epigenomics/
9. Histones/ or DNA Methylation/ or epigenome.mp. or Epigenesis, Genetic/ or Epigenomics/
10. 6 or 7 or 8 or 9
11. 5 and 10
12. limit 11 to "all child (0 to 18 years)"

EMBASE (OvidSP)

1. Inflammatory bowel disease.mp. or Inflammatory Bowel Diseases/
2. Crohn disease.mp. or Crohn Disease/
3. Ulcerative colitis.mp. or Colitis, Ulcerative/
4. Colitis, Ulcerative/ or Inflammatory Bowel Diseases/ or IBD.mp. or Crohn Disease/
5. 1 or 2 or 3 or 4
6. Methylation/ or DNA Methylation/ or methylation.mp.
7. Interaction$.mp. or Gene-Environment Interaction/
8. Epigenetic$.mp. or Epigenomics/
9. Histones/ or DNA Methylation/ or epigenome.mp. or Epigenesis, Genetic/ or Epigenomics/
10. 6 or 7 or 8 or 9
11. 5 and 10
12. limit 11 to "all child (0 to 18 years)"

Supplementary Table S2 Characteristics of included genetic association studies in paediatric IBD

Author	Year	Study location	Disease	Age cutoff (below)	Mean age of PIBD cases (yrs)	Sample size		Gene
						Cases	Controls	
Sun L [1]	2003	Germany	CD	18	11.2	55	101	*NOD2*
Tomer G [2]	2003	USA	CD	18	11.8	87	136	*NOD2*
Kugathasan S* [3]	2005	USA	CD	18	12.1	58	124	*NOD2*
Kugathasan S* [3]	2005	USA	CD	18	12.1	164	601	*NOD2*
Levine A [4]	2005	Israel	CD	18	12.8	83	100	*NOD2, TNF-α*
Russell RK [5]	2005	UK	CD/UC	16	11.0	167/60	245	*NOD2*
Ferraris A [6]	2006	Italy	CD/UC	18	12.0	134/93	164	*NOD2, SCL22A4/5, DLG5*
Russell RK [7]	2006	UK	CD/UC	16	11.2	200/74	256	*IBD5, SCL22A4/5*
Sýkora J [8]	2006	Czech	CD/UC	18	15.3	46/34	82	*TNF-α*
Baldassano RN [9]	2007	USA	CD	17	10.9	142	281	*NOD2, ATG16L1*
Browning BL [10]	2007	New Zealand	CD/UC	17	n/a	38/26	415	*DLG5*
Cucchiara S [11]	2007	Italy	CD/UC	18	12.0	200/186	347	*MDR1, TNF-α*
Cucchiara S [12]	2007	Italy	CD/UC	18	12.0	200/186	347	*NOD2,IBD5,SCL22A4/5,DLG5*
Cummings JR [13]	2007	UK	CD	16	n/a	71/32	1190	*ATG16L1*
De Iudicibus S [14]	2007	Italy	CD/UC	17	n/a	64/55	100	*hGR, MDR1*
de Ridder L [15]	2007	Netherland	CD/UC	18	12.0	72/31	272	*NOD2,TLR4,SCL22A4/5,DLG5*
Gearry RB [16]	2007	New Zealand	CD	17	n/a	77	201	*NOD2*
Glas J [17]	2007	Germany	CD	17	n/a	53	1381	*IL23R*
Leshinsky SE [18]	2007	Israel	CD	17	n/a	143	157	*IL23R*
Lacher M [19]	2007	Germany	CD/UC	18	11.5	78/30	120	*CXCL9*
Nam SY [20]	2007	Korea	CD	17	n/a	15	101	*HSP70-2*
Roberts RL [21]	2007	New Zealand	CD	17	n/a	55	591	*IL23R, ATG16L1*
Van Limbergen J [22]	2007	UK	CD/UC	17	n/a	223/82	355	*NOD1/CARD4*
Van Limbergen J [23]	2007	UK	CD/UC	17	11.1	233/86	342	*IL23R*
Van Limbergen J [24]	2007	UK	CD/UC	17	n/a	228/90	1233	*NOD1/CARD4*
Ferguson LR [25]	2008	New Zealand	CD/UC	17	n/a	39/26	201	*DEFA5*
Ferguson LR [26]	2008	New Zealand	CD/UC	17	n/a	39/26	201	*TNF-α*
Glas J [27]	2008	Germany	CD	17	n/a	49	1615	*ATG16L1*
Hradsky O [28]	2008	Czech	CD	17	13.5	136	501	*NOD2, TNF-α, PTPN22*
Latiano A [29]	2008	Italy	CD/UC	17	n/a	173/155	749	*ATG16L1, IL23R*
Perricone C [30]	2008	Italy	CD	17	n/a	20	160	*ATG16L1*
Seiderer J [31]	2008	Germany	CD/UC	17	n/a	50/12	967	*IL-17F*
Van Limbergen J [32]	2008	UK	CD	17	11.2	269	345	*ATG16L1*
Chen B[33]	2009	China	CD/UC	17	n/a	10	373	*IL-17F*
De Mesquita [34]	2009	Belgium	CD/UC	17	n/a	80/15	76	*NOD2, TLR4*
Ferguson LR [35]	2009	New Zealand	CD/UC	17	n/a	39/26	293	*TNFRSF1B*
Huebner C [36]	2009	New Zealand	CD/UC	17	n/a	39/26	201	*NOD1/CARD4*
Koslowski MJ [37]	2009	Austria, Belgium, UK	CD	16	n/a	81	242	*TCF-4*
Lacher M [38]	2009	Germany	IBD	17	n/a	187	185	*PXR*
Lacher M [39]	2009	Germany	CD	17	10.9	152	253	*ATG16L1*
Latiano A [40]	2009	Italy	CD/UC	17	n/a	245/130	578	*IRGM*
Tomer G [41]	2009	USA	CD	18	11.7	83	75	*SCL22A4/5, IGR*
Török HP [42]	2009	Germany	CD	16	n/a	48	792	*TLR9*
Van Limbergen J [43]	2009	UK	CD	17	n/a	272	362	*IRGM*
Van Limbergen J [44]	2009	UK	CD/UC	17	11.3	245/92	996	*FLG*
Amre DK [45]	2010	Canada	CD	18	12.3	406	415	*ZNF365,PTGER4,IL12B,STAT3, PTPN2*
Aoyagi Y [46]	2010	Canadian	UC	17	n/a	8	8	*PPARg*
Ferguson LR [47]	2010	New Zealand	CD	17	n/a	29	382	*JAK2, STAT3*
Ferguson LR [48]	2010	New Zealand	CD	17	n/a	30	369	*IL-12B, IL23R*
Gazouli M [49]	2010	Greece	CD	17	11.5	110	539	*NOD2, ATG16L1*
Glas J [50]	2010	Germany	CD	17	n/a	344	1446	*NOD2*
Glas J [51]	2010	Germany	CD	17	n/a	70	1383	*STAT4*
Lacher M [52]	2010	Germany	CD	17	11.8	152	152	*NOD2*
Lacher M [53]	2010	Germany	CD/UC	17	11.6	221/132	253	*NOD2, IL23R*
Lacher M[54]	2010	Germany	CD	17	11.8	171	253	*NOD2*
Latiano A [55]	2010	Italy	CD/UC	17	n/a	283/256	651	*MST1, 3p21*
Morgan AR [56]	2010	New Zealand	CD	17	n/a	29	481	*PTPN22, PTPN2*
Roberts RL [57]	2010	New Zealand	CD	17	n/a	57/28	517	*CARD8, NALP3*
Schroepf S [58]	2010	Germany	CD/UC	17	11.0	167	231	*NOD2, ART3/CXCL11*
Wagner J [59]	2010	Australia	CD	18	11.6	72	98	*NOD2,IL23R,3p21,PSMG1,TNFRSF6B*
Bak-Romaniszyn L [60]	2011	Poland	CD/UC	18	13.7	30/26	78	*MBL2*
Diaz-Gallo LM [61]	2011	Spain	CD/UC	16	n/a	24/7	629	*CD24*
Latiano A [62]	2011	Italy	CD/UC	17	n/a	296/261	789	*PTGER4,TNFSF15,NKX2-*

								3,IFNG,PTPN2,PSMG1, ZNF365
Repnik K [63]	2011	Slovenia	CD/IBD	17	n/a	43/78	299	*SCL22A4/5*
Wang AH [64]	2011	New Zealand	CD	17	n/a	30	610	*IL-10*
Wolters VM [65]	2011	Canada	IBD	18	n/a	648	924	*MAGI2, PARD3, MYO9B*
de Vries HS [66]	2012	Netherland	CD	16	n/a	53	930	*UGT1A1*
Glas J [67]	2012	Germany	CD/UC	17	n/a	168/59	908	*PTPN2*
Glas J [68]	2012	Germany	CD/UC	17	n/a	211	1488	*PTGER4*
Mazzoccoli G [69]	2012	Italy	CD/UC	18	n/a	402/352	412	*PER3*
Morgan AR [70]	2012	New Zealand	CD	17	n/a	46	638	*TLR10*
Morgan AR [71]	2012	New Zealand	CD	17	n/a	46	638	*ULK1*
Muise AM [72]	2012	Canada	IBD	10	n/a	268	480	*NCF2*
Chen J [73]	2013	China	CD	17	n/a	8	190	*HSP70-2*
Falvey JD [74]	2013	New Zealand	CD/UC	17	n/a	57/28	340	*MIF*
Hirano A [75]	2013	Japan	CD	17	n/a	159	6585	*TNFSF15*
Luo YY [76]	2013	China	CD	17	12.7	19	122	*ApaI, TaqI, BsmI*
Wagner J [77]	2013	Australia	CD	17	n/a	62	46	*PSMG1,TNFRSF6B,NOD2 ,NOD1,IBD5,IL23R,IL10R A,DLG5,MYO9B,SLC22A4 /5,TLR4,ATG16L1,ABCB1, 10q21.1,NELL1,3p21, IRGM, NKX2.3*
Dhillon SS [78]	2014	Canada	IBD	6	n/a	159	913	*NOS2*
Hu J [79]	2014	China	CD	16	n/a	10	407	*MYO9B*
Jakobsen C [80]	2014	Danmark	CD/UC	18	n/a	244/318	543	*ATG16L1, NOD2, ZMIZ1, SLC7A10, XBP1, IBD5, IL27, IL2, ZNF365,TNFSF15*
Serbati N [81]	2014	Morocco	CD	17	n/a	10	115	*ATG16L1, IL23R*
Serbati N [82]	2014	Morocco	CD	17	n/a	11	114	*NOD2*
Senhaji N [83]	2015	Morocco	CD	17	n/a	4	100	*MDR1*
Schnitzler F [84]	2015	German	CD	17	n/a	160	719	*NOD2*

*Data were extracted separately for American Africans and white population.

Supplementary Table S3 Summary of the variants identified for paediatric IBD in genetic association studies*

No. of studies	Gene	Polymorphism/ rs number	Candidate gene study (p-value)	Genome-wide significance in GWAS
**147 polymorphisms in 58 genes identified for paediatric CD**				
16	*NOD2/CARD15*	rs2066844	Meta-analysis	YES
16	*NOD2/CARD15*	rs2066845	Meta-analysis	YES
16	*NOD2/CARD15*	rs2066847	Meta-analysis	YES
12	*ATG16L1*	rs2241880	Meta-analysis	YES
10	*IL23R*	rs11209026	Meta-analysis	YES
7	*IBD5*	rs1050152	Meta-analysis	NA
5	*IBD5*	rs26313667	Meta-analysis	NA
5	*TNF-α*	rs1800629	Meta-analysis	NA
4	*DLG5*	rs1248696	Meta-analysis	NA
4	*IBD5*	rs11739135	Meta-analysis	NA
4	*IBD5*	rs12521868	Meta-analysis	YES
4	*IL23R*	rs7517847	Meta-analysis	YES
4	*PTPN2*	rs2542151	Meta-analysis	YES
3	*BSN*	rs9858542	Meta-analysis	YES
3	*DLG5*	rs2289311	Meta-analysis	NA
3	*IBD5*	rs17622208	Meta-analysis	NA
3	*NOD2/CARD15*	rs5743289	Meta-analysis	YES
3	*PSMG1*	rs2836878	Meta-analysis	YES
3	*TLR4*	rs4986790	Meta-analysis	NA
3	*TNF-α*	rs1799724	Meta-analysis	NA
2	*DLG5*	rs1270912	NS/NS	NA
2	*DLG5*	rs2165047	NS/NS	NA
2	*IL-17F*	rs763780	NS/NS	NA
2	*IL23R*	rs1004819	NS/NS	YES

2	*IRGM*	rs13361189	NS/NS	YES
2	*IRGM*	rs4958847	0.008/NS	NA
2	*MDR1*	rs1045642	NS/NS	NA
2	*MYO9B (exon 20)*	rs1545620	NS/NS	NA
2	*NOD2/CARD15*	rs2066843	NS/NS	YES
2	*NOD2/CARD15*	rs2076756	NS/NS	YES
2	*PTGER4*	rs4613763	NS/NS	YES
2	*PTPN22*	rs2476601	NS/NS	NA
2	*SLC22A4/5*	rs3792876	0.009/NS	NA
2	*STAT3*	rs744166	0.014/NS	YES
2	*TNFRSF6B*	rs2315008	NS/NS	YES
2	*TNFRSF6B*	rs4809330	NS/NS	YES
2	*TNF-α*	rs361525	NS/NS	NA
1	*10q21.1*	rs2241136	NS	NA
1	*ABCB1*	rs17327442	NS	NA
1	*ART3/CXCL11*	rs6817952	NS	NA
1	*CARD8*	rs2043211	0.002	NA
1	*CD24*	rs3838646	0.040	NA
1	*CD24*	rs8734	NS	NA
1	*CYBA*	rs72550704	0.005	NA
1	*DEFA5*	rs10095331	NS	NA
1	*DEFA5*	rs12682030	0.034	NA
1	*DEFA5*	rs4610776	NS	NA
1	*DEFA5*	rs7017866	NS	NA
1	*DLG5*	rs1344966	NS	NA
1	*DLG5*	rs2289310	NS	NA
1	*FLG*	rs41370446	NS	NA
1	*FLG*	rs61816761	NS	NA
1	*GSTM1*	GSTM1 (null or non-null genotype)	NS	NA
1	*GSTT1*	GSTT1(null or non-null genotype)	0.013	NA
1	*hGR*	rs41423247	NS	NA
1	*HLA, BTNL2*	rs2395185	NS	NA
1	*HSP70-2*	rs1061581	NS	NA
1	*HSP70-2*	rs539689	NS	NA
1	*IFNG, IL22, IL26*	rs1558744	NS	NA
1	*IL10*	rs1800871	NS	NA
1	*IL10*	rs1800872	NS	NA
1	*IL10*	rs1800896	NS	NA
1	*IL10*	rs3024505	NS	YES
1	*IL10RA*	rs2229113	NS	NA
1	*IL10RA*	rs3135932	NS	NA
1	*IL-12B*	rs10045431	NS	YES
1	*IL-12B*	rs1363670	NS	NA
1	*IL-12B*	rs6887695	NS	YES
1	*IL23R*	rs10889677	NS	YES
1	*IL23R*	rs11805303	NS	YES
1	*IL23R*	rs1343151	0.007	YES
1	*IL23R*	rs7530511	NS	NA
1	*IRGM*	rs1000113	0.000	YES
1	*IRGM*	rs10065172	NS	NA
1	*MBL2*	rs1800450	$p < 0.05$	NA
1	*MDR1*	rs1128503	NS	NA

1	*MIF*	rs5844572	NS	NA
1	*MIF*	rs755622	NS	NA
1	*MST1*	rs3197999	0.000	YES
1	*MYO9B*	rs962917	NS	NA
1	*MYO9B (intron 14)*	rs2305764	NS	NA
1	*NALP3*	rs3582941	NS	NA
1	*NELL1*	rs1793004	NS	YES
1	*NKX2-3*	rs10883365	NS	YES
1	*NKX2-3*	rs11190140	NS	YES
1	*NOD1/CARD4*	rs1558066	NS	NA
1	*NOD1/CARD4*	rs2075818	NS	NA
1	*NOD1/CARD4*	rs2075820	NS	NA
1	*NOD1/CARD4*	rs2075822	NS	NA
1	*NOD1/CARD4*	rs2529445	NS	NA
1	*NOD1/CARD4*	rs2709799	NS	NA
1	*NOD1/CARD4*	rs2907748	NS	NA
1	*NOD1/CARD4*	rs2970500	NS	NA
1	*NOD1/CARD4*	rs38403	NS	NA
1	*NOD1/CARD4*	rs4720004	NS	NA
1	*NOD1/CARD4*	rs6958571	NS	NA
1	*NOD1/CARD4*	rs7789045	NS	NA
1	*NOD1/CARD4*	rs932272	NS	NA
1	*NOD2/CARD15*	rs2066842	NS	NA
1	*NOD2/CARD15*	rs5743271	NS	NA
1	*NOD2/CARD15*	rs5743291	NS	NA
1	*NOD2/CARD15*	rs5743293	NS	NA
1	*NOD2/CARD15*	rs72796353	NS	NA
1	*PERIOD3*	rs2797685	0.002	YES
1	*PTGER4*	rs4495224	0.036(AA VS. AC)	NA
1	*PTGER4*	rs7720838	NS	NA
1	*PTPN2*	rs1893217	0.005	YES
1	*PTPN2*	rs7234029	0.043	NA
1	*SLC22A4/5*	rs2631372	NS	NA
1	*SLC22A4/5*	rs272893	NS	NA
1	*SLC22A4/5*	rs273900	NS	NA
1	*SLC22A4/5*	rs274551	NS	NA
1	*SLC7A10*	rs10500264	NS	YES
1	*STAT3*	rs10758669	NS	YES
1	*STAT4*	rs3816769	NS	NA
1	*STAT4*	rs7574865	NS	NA
1	*TCF-4*	rs3814570	NS	NA
1	*TLR10*	rs10024216	NS	NA
1	*TLR10*	rs10856838	NS	NA
1	*TLR10*	rs11466657	NS	NA
1	*TLR10*	rs4274855	NS	NA
1	*TLR10*	rs6841698	NS	NA
1	*TLR10*	rs7653908	NS	NA
1	*TLR10*	rs7658893	NS	NA
1	*TLR4*	rs4986791	NS	NA
1	*TLR9*	rs5743836	NS	NA
1	*TNFRSF1B*	rs1061622	NS	NA
1	*TNFRSF1B*	rs1061624	NS	NA

1	*TNFRSF1B*	rs3397	NS	NA
1	*TNFSF15*	rs3810936	0.018	YES
1	*TNFSF15*	rs4263839	NS	YES
1	*TNF-α*	rs1800630	NS	NA
1	*UGT1A1*	rs8175347	NS	NA
1	*ULK1*	rs10902469	NS	NA
1	*ULK1*	rs11616018	NS	NA
1	*ULK1*	rs12303764	NS	NA
1	*ULK1*	rs3088051	NS	NA
1	*ULK1*	rs3923716	NS	NA
1	*ULK1*	rs7488085	NS	NA
1	*ULK1*	rs7953348	NS	NA
1	*VDR*	rs1544410	NS	NA
1	*VDR*	rs731236	NS	NA
1	*VDR*	rs7975232	NS	NA
1	*XBP1*	rs5762839	NS	NA
1	*ZMIZ1*	rs1250550	0.010	YES
1	*ZNF365*	rs10761659	0.007	YES
1	*ZNF365*	rs10995271	0.001	YES
**80 polymorphisms in 40 genes identified for paediatric UC**				
6	*NOD2/CARD15*	rs2066844	Meta-analysis	NA
6	*NOD2/CARD15*	rs2066845	Meta-analysis	YES
6	*NOD2/CARD15*	rs2066847	Meta-analysis	NA
4	*IBD5*	rs1050152	Meta-analysis	NA
4	*IBD5*	rs26313667	Meta-analysis	NA
3	*DLG5*	rs1248696	Meta-analysis	YES
3	*IL23R*	rs11209026	Meta-analysis	YES
3	*TNF-α*	rs1800629	Meta-analysis	NA
2	*DLG5*	rs2289311	NS/NS	NA
2	*IBD5*	rs11739135	NS/NS	NA
2	*IBD5*	rs12521868	NS/NS	NA
2	*IL17F*	rs763780	NS/NS	NA
2	*IL23R*	rs7517847	NS/NS	NA
2	*MDR1*	rs1045642	NS/NS	NA
2	*PTPN2*	rs2542151	0.043/NS	NA
2	*TLR4*	rs4986790	NS/NS	NA
2	*TNF-α*	rs1799724	NS/NS	NA
1	*ART3/CXCL11*	rs6817952	NS	NA
1	*ATG16L1*	rs2241880	NS	NA
1	*BSN*	rs9858542	0.002	NA
1	*CATT5-8*	rs5844572	NS	NA
1	*CD24*	rs3838646	NS	NA
1	*CD24*	rs8734	NS	NA
1	*CXCL9*	rs2276886	NS	NA
1	*DEFA5*	rs10095331	NS	NA
1	*DEFA5*	rs12682030	NS	NA
1	*DEFA5*	rs4610776	NS	NA
1	*DEFA5*	rs7017866	NS	NA
1	*DLG5*	rs1270912	NS	NA
1	*DLG5*	rs2165047	NS	NA
1	*FLG*	rs41370446	NS	NA

1	*FLG*	rs61816761	NS	NA
1	*hGR*	rs41423247	NS	NA
1	*HLA, BTNL2*	rs2395185	0.002	YES
1	*IBD5*	rs17622208	NS	NA
1	*IBD5*	rs2201841	NS	NA
1	*IFNG, IL22, IL26*	rs1558744	0.008	NA
1	*IL2/IL21*	rs6840978	NS	NA
1	*IL27*	rs1968752	0.050	NA
1	*IRGM*	rs1000113	NS	NA
1	*IRGM*	rs4958847	NS	NA
1	*MBL2*	Rs1800450	NS	NA
1	*MIF*	rs755622	NS	NA
1	*MST1*	rs3197999	0.003	YES
1	*MYO9B (exon 20)*	rs1545620	NS	YES
1	*NCF2*	rs35012521	6.89E-03	NA
1	*NKX2-3*	rs11190140	4.00E-05	NA
1	*NOD1/CARD4*	rs1558066	NS	YES
1	*NOD1/CARD4*	rs2075818	NS	NA
1	*NOD1/CARD4*	rs2075820	NS	NA
1	*NOD1/CARD4*	rs2075822	NS	NA
1	*NOD1/CARD4*	rs2529445	NS	NA
1	*NOD1/CARD4*	rs2709799	NS	NA
1	*NOD1/CARD4*	rs2907748	NS	NA
1	*NOD1/CARD4*	rs2970500	NS	NA
1	*NOD1/CARD4*	rs38403	NS	NA
1	*NOD1/CARD4*	rs4720004	NS	YES
1	*NOD1/CARD4*	rs7789045	NS	NA
1	*NOD1/CARD4*	rs932272	NS	NA
1	*PERIOD3*	rs2797685	NS	NA
1	*PPARg*	rs1801282	NS	NA
1	*PSMG1*	rs2836878	0.047	YES
1	*PTGER4*	rs4495224	NS	NA
1	*PTGER4*	rs4613763	0.013	NA
1	*PTGER4*	rs7720838	NS	NA
1	*PTPN2*	rs7234029	NS	NA
1	*RAC2*	rs1476002	2.16E-04	NA
1	*SLC22A4/5*	rs272893	NS	NA
1	*SLC22A4/5*	rs273900	NS	NA
1	*SLC22A4/5*	rs274551	NS	NA
1	*SLC22A4/5*	rs3792876	NS	NA
1	*TLR4*	rs4986791	NS	NA
1	*TNFRSF1B*	rs1061622	NS	NA
1	*TNFRSF1B*	rs1061624	NS	NA
1	*TNFRSF1B*	rs3397	NS	NA
1	*TNFSF15*	rs4263839	NS	NA
1	*TNFSF15*	rs6478108	NS	NA
1	*TNF-α*	rs361525	NS	NA
1	*ZNF365*	rs10761659	4.00E-05	YES
1	*ZNF365*	rs10995271	NS	NA
**29 polymorphisms in 8 genes identified for paediatric IBD**				
1	*NOS2*	rs10459953	NS	NA

1	*SLC22A4/5*	rs1050152	NS	NA
1	*NOS2*	rs11080344	NS	NA
1	*NOS2*	rs1137933	7.40E-04	NA
1	*NOS2*	rs11653716	NS	NA
1	*DLG5*	rs1248696	NS	NA
1	*MYO9B (intron 20)*	rs1457092	NS	NA
1	*MYO9B (exon 20)*	rs1545620	NS	NA
1	*MYO9B (intron 32)*	rs2279002	NS	NA
1	*NOS2*	rs2297516	6.20E-06	NA
1	*NOS2*	rs2297518	NS	NA
1	*MYO9B (intron 28)*	rs2305764	NS	NA
1	*MYO9B (intron 14)*	rs2305767	0.039	NA
1	*NOS2*	rs2314809	NS	NA
1	*NOS2*	rs2314810	NS	NA
1	*SLC22A4/5*	rs2631372	NS	NA
1	*NOS2*	rs3729508	NS	NA
1	*NOS2*	rs3730013	NS	NA
1	*NOS2*	rs3730017	NS	NA
1	*NOS2*	rs3794756	NS	NA
1	*NOS2*	rs3794764	NS	NA
1	*PXR*	rs3814055	NS	NA
1	*PARD3*	rs4379776	NS	NA
1	*NOS2*	rs4795067	NS	NA
1	*MAGI2*	rs6962966	NS	NA
1	*NOS2*	rs8072199	NS	NA
1	*NOS2*	rs944725	0.030	NA
1	*MYO9B*	rs962917	NS	NA
1	*NOS2*	rs9906835	NS	NA

Notes:

1. Variants (highlighted in green) were reported to be significantly associated with paediatric CD in at least one candidate gene study or GWAS accordingly.
2. For variants investigated in more than three candidate gene studies, we conducted meta-analyses; please see the p-values for individual studies and meta-analyses in the main text and supplementary figures.
3. Meta-analyses highlighted in green mean at least one of the included studies reported significant associations; meta-analyses without being highlighted mean none of the included studies reported significant associations
4. Genome-wide significance in GWAS was checked in GWAS catalog: https://www.ebi.ac.uk/gwas/
5. NS: No Significance; NA: Not Available

Supplementary Table S4 Linkage disequilibrium (LD) between identified variants *

**SNPs**	rs11739135	rs12521868	rs1050152	rs2631367
rs11739135	1	***0.830***	***0.902***	0.564
rs12521868	***0.830***	1	***0.934***	0.584
rs1050152	***0.902***	***0.934***	1	***0.625***
rs26313667	0.564	0.584	***0.625***	1

* Values presented in table were r^2.

Supplementary Table S5 Sensitivity analysis in dominant model by excluding study conducted in non-white population and study violating HWE

Gene/ Variant	DOMINANT MODEL: *wt/var & var/var* vs. *wt/wt*					Credibility Assessment			
	No. of studies	Cases (+/-)	Controls (+/-)	OR (95% CI)	*P* value	I^2 (95% CI)	Power	BFDP ‡	Venice criteria grade¶
**Ethnicity (paediatric CD)**									
**NOD2/rs2066844***	**15**	**318/1608**	**334/3721**	**2.30 (1.78, 2.96)**	**1.19E-10**	**47(1, 81)**	**1.00**	**0.000**	**BAB**
NOD2/rs2066847	15	430/1492	203/3907	5.90(3.72, 9.37)	5.07E-14	78(61, 93)	**1.00**	0.000	CAB
**HWE (paediatric CD)**									
**NOD2/rs2066844***	**14**	**300/1517**	**314/3620**	**2.38(1.85, 3.07)**	**1.58E-11**	**41(0, 82)**	**1.00**	**0.000**	**BAB**
ATG16L1*/rs2241880	**11**	**867/483**	**4432/1838**	**0.70(0.61, 0.80)**	**2.02E-07**	**0(0, 55)**	**1.00**	**0.000**	**BAB**
**HWE & Ethnicity (paediatric CD)**									
**NOD2/rs2066844***	**14**	**299/1460**	**310/3500**	**2.38(1.99, 2.84)**	**2.36E-21**	**41(0, 80)**	**1.00**	**0.000**	**BAA**
**HWE (paediatric UC)**									
NOD2/rs2066844	5	65/465	91/1176	1.84(1.31, 2.59)	4.83E-04	15(0, 91)	0.93	0.196	AAB

*Associations are classified as highly credible.

‡ Bayesian False Discovery Probability (BFDP) value were calculated at prior probability of 0.05. BFDP level of noteworthiness is 0.2.

¶ Venice criteria grade for the three criteria. The first grade is for the amount of evidence assessed according to statistical power (A, ≥80%; B, 50%-79%; C, <50%); the second grade is for the extent of replication assessed according to heterogeneity (I^2 value: A, <25%; B, 25%-50%; C, >50%); the third grade is for protection from bias assessed according to small study effect (complete assessment of bias is difficult, no variants were graded as "A"; "B" was assigned for studies which no small study effect was detected; otherwise, "C" was assigned).

Supplementary Table S6 Sensitivity analysis in recessive model by excluding study conducted in non-white population and study violating HWE

Gene/ Variant	RECESSIVE MODEL: var/var vs. wt/wt & wt/var					Credibility Assessment			
	No. of studies	Cases (+/-)	Controls (+/-)	OR (95% CI)	*P* value	I^2 (95% CI)	Power	BFDP ‡	Venice criteria grade¶
**Ethnicity (paediatric CD)**									
**NOD2/rs2066844***	**12**	**30/1737**	**15/3744**	**4.14(2.18, 7.89)**	**1.54E-05**	**0(0, 49)**	**0.99**	**0.035**	**AAB**
**NOD2/rs2066847***	**10**	**76/1370**	**3/3247**	**18.83(8.21, 43.22)**	**4.23E-12**	**0(0, 57)**	**1.00**	**0.000**	**AAB**
**HWE (paediatric CD)**									
**NOD2/rs2066844***	**11**	**28/1572**	**12/3502**	**5.25(2.57, 10.74)**	**5.64E-06**	**0(0, 39)**	**1.00**	**0.024**	**AAB**
**ATG16L1/rs2241880***	**11**	**230/1120**	**1314/4956**	**0.74(0.63, 0.87)**	**3.30E-04**	**0(0, 60)**	**0.87**	**0.163**	**AAB**
**HWE & Ethnicity (paediatric CD)**									
**NOD2/rs2066844***	**11**	**28/1572**	**12/3502**	**5.25(2.57, 10.73)**	**5.64E-06**	**0(0, 39)**	**1.00**	**0.024**	**AAB**
**HWE (paediatric UC)**									
NOD2/rs2066844	3	0/346	4/868	0.86(0.14, 5.31)	0.871	0(0, 94)	0.05	0.957	ACB

*Associations are classified as highly credible.

‡ Bayesian False Discovery Probability (BFDP) value were calculated at prior probability of 0.05. BFDP level of noteworthiness is 0.2.

¶ Venice criteria grade for the three criteria. The first grade is for the amount of evidence assessed according to statistical power (A, ≥80%; B, 50%-79%; C, <50%); the second grade is for the extent of replication assessed according to heterogeneity (I^2 value: A, <25%; B, 25%-50%; C, >50%); the third grade is for protection from bias assessed according to small study effect (complete assessment of bias is difficult, no variants were graded as "A"; "B" was assigned for studies which no small study effect was detected; otherwise, "C" was assigned).

Supplementary Table S7 Sensitivity analysis in additive model 1 by excluding study conducted in non-white population and study violating HWE

Gene/ Variant	ADDITIVE MODEL 1: *var/wt* vs. *wt/wt*					Credibility Assessment			
	No. of studies	Cases (+/-)	Controls (+/-)	OR (95% CI)	*P* value	I² (95% CI)	Power	BFDP ‡	Venice criteria grade¶
**Ethnicity (paediatric CD)**									
**NOD2/rs2066844***	**15**	**288/1608**	**319/3721**	**2.14(1.80, 2.56)**	**4.63E-17**	**36(0, 77)**	**1.00**	**0.000**	**BAB**
NOD2/rs2066847	15	353/1492	199/3907	5.11(3.36, 7.76)	2.06E-14	72(50, 91)	1.00	0.000	CAB
**HWE (paediatric CD)**									
**NOD2/rs2066844***	**15**	**272/1517**	**302/3620**	**2.21(1.84, 2.66)**	**1.96E-17**	**31(0, 79)**	**1.00**	**0.000**	**BAB**
**ATG16L1/rs2241880***	**11**	**637/483**	**3118/1838**	**0.74(0.64, 0.85)**	**2.90E-05**	**0(0, 29)**	**0.98**	**0.020**	**AAB**
**HWE & Ethnicity (paediatric CD)**									
**NOD2/rs2066844***	**14**	**271/1460**	**298/3500**	**2.24(1.86, 2.70)**	**1.02E-17**	**31(0, 77)**	**1.00**	**0.000**	**BAB**
**HWE (paediatric UC)**									
NOD2/rs2066844	5	0/530	4/1263	1.92(1.36, 2.71)	2.07E-04	13(0, 91)	0.18	0.108	ACB

*Associations are classified as highly credible.

‡ Bayesian False Discovery Probability (BFDP) value were calculated at prior probability of 0.05. BFDP level of noteworthiness is 0.2.

¶ Venice criteria grade for the three criteria. The first grade is for the amount of evidence assessed according to statistical power (A, ≥80%; B, 50%-79%; C, <50%); the second grade is for the extent of replication assessed according to heterogeneity (I^2 value: A, <25%; B, 25%-50%; C, >50%); the third grade is for protection from bias assessed according to small study effect (complete assessment of bias is difficult, no variants were graded as "A"; "B" was assigned for studies which no small study effect was detected; otherwise, "C" was assigned).

Supplementary Table S8 Sensitivity analysis in additive model 2 by excluding study conducted in non-white population and study violating HWE

Gene/ Variant	ADDITIVE MODEL 2: *var/var vs. wt/wt*					Credibility Assessment			
	No. of studies	Cases (+/-)	Controls (+/-)	OR (95% CI)	*P* value	I² (95% CI)	Power	BFDP ‡	Venice criteria grade¶
**Ethnicity (paediatric CD)**									
**NOD2/rs2066844***	**12**	**274/1463**	**307/3437**	**4.44(2.34, 8.43)**	**5.26E-06**	**0(0, 54)**	**1.00**	**0.016**	**AAB**
**NOD2/rs2066847***	**10**	**312/1057**	**169/3078**	**23.42(10.20, 53.78)**	**1.04E-13**	**0(0, 67)**	**1.00**	**0.000**	**AAB**
**HWE (paediatric CD)**									
**NOD2/rs2066844***	**11**	**28/1315**	**12/3216**	**5.68(2.79, 11.58)**	**1.76E-06**	**0(0, 44)**	**1.00**	**0.011**	**AAB**
**ATG16L1/rs2241880***	**11**	**230/483**	**1314/1838**	**0.61(0.51, 0.74)**	**3.41E-07**	**12(0, 68)**	**1.00**	**0.001**	**AAB**
**HWE & Ethnicity (paediatric CD)**									
**NOD2/rs2066844***	**11**	**28/1315**	**12/3216**	**5.68(2.79, 11.58)**	**1.76E-06**	**0(0, 44)**	**1.00**	**0.011**	**AAB**
**HWE (paediatric UC)**									
NOD2/rs2066844	3	0/303	4/805	0.94(0.15, 5.83)	0.950	0(0, 95)	0.05	0.957	ACB

* Associations are classified as highly credible.

‡ Bayesian False Discovery Probability (BFDP) value were calculated at prior probability of 0.05. BFDP level of noteworthiness is 0.2.

¶ Venice criteria grade for the three criteria. The first grade is for the amount of evidence assessed according to statistical power (A, ≥80%; B, 50%-79%; C, <50%); the second grade is for the extent of replication assessed according to heterogeneity (I^2 value: A, <25%; B, 25%-50%; C, >50%); the third grade is for protection from bias assessed according to small study effect (complete assessment of bias is difficult, no variants were graded as "A"; "B" was assigned for studies which no small study effect was detected; otherwise, "C" was assigned).

Supplementary Table S9 Epigenetic studies about DNA methylation and MicroRNA expression in paediatric IBD

Authors, Year	Subjects	Study design	Samples	Techniques	No. if loci with differential DNA methylation or dysregulated miRNA expression
**DNA methylation studies in peripheral blood leukocyte and biopsy specimens**					
Adams *et al*, 2014 [85]	24 paediatric CD; 19 controls	Case-control; Genome-wide DNA methylation analysis	Peripheral blood leukocyte	Illumina Human Methylation 450 BeadChip arrays	65 CpG sites in 46 genes achieving epigenome-wide significance
Harris *et al*, 2012 [86]	14 paediatric CD, 8 paediatric UC, 14 controls	Case-control; Genome-wide DNA methylation analysis	Peripheral blood leukocyte	Illumina Human Methylation 450 BeadChip arrays	No differential methylation regions (DMRs) associated with paediatric CD; 6 CpG sites in 5 genes displaying methylation difference associated with paediatric UC
Harris *et al,* 2014 [87]	Discovery cohort: 10 controls, 10 Paediatric CD, 4 paediatric UC Validation cohort: 12 controls, 5 paediatric CD, 5 paediatric UC	Case-control; Genome-wide DNA methylation analysis	Colonic mucosal biopsies	Illumina Human Methylation 450 BeadChip arrays	108 differentially methylated genes associated with paediatric CD; 2,243 differentially methylated genes associated with paediatric UC;
**miRNA expression studies in biopsy specimens**					
Koukos *et al,* 2013 [88]	33 paediatric IBD (18 active UC, 9 inactive UC and 6 CD); 12 controls	Case-control; Assessing expression levels of 316 miRNAs; Testing DNA-methylation level of *MIR124*	Sigmoid biopsies	RT-PCR; quantitative methylation-specific PCR analysis	5 dysregulated miRNA associated paediatric UC
Koukos *et al,* 2015 [89]	5 paediatric UC; 5 non-IBD controls	Case-control;	Sigmoid biopsies	RT-PCR; miRCURY microRNA Array Profiling	23 dysregulated miRNA associated paediatric UC

Supplementary Figures S1-S57

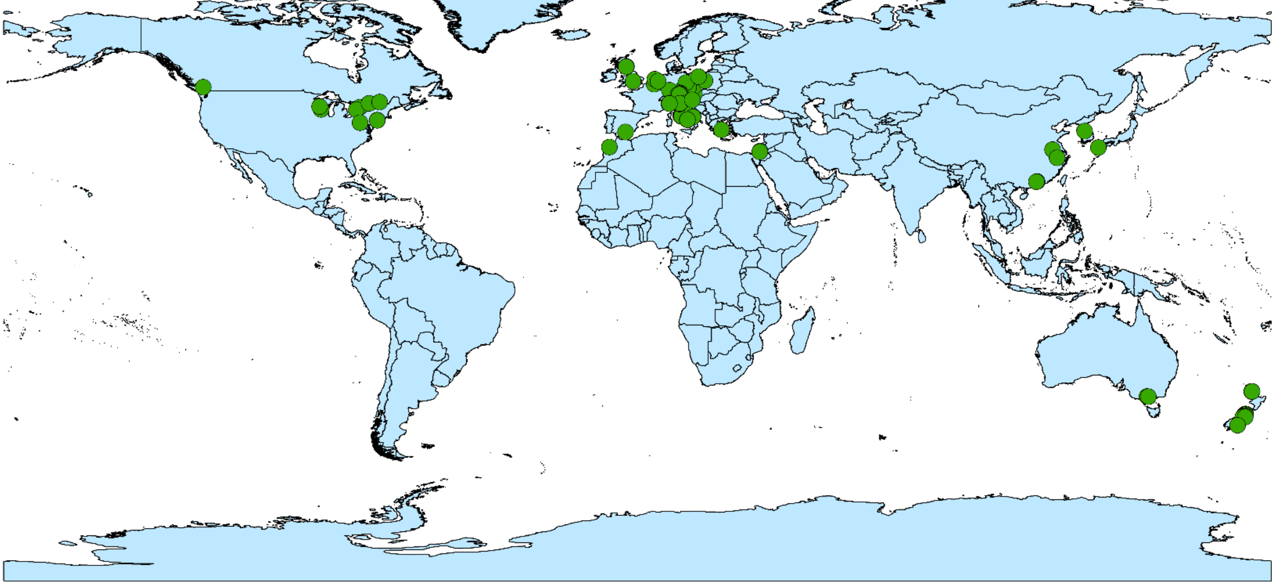

Supplementary Figure S1 The geographic distribution of study populations. This map was generated by software ArcGIS 10.1 (www.esri.com) based on the open-accessed "World Cities" layer package provided by Eris, Delorme Publishing Company, Inc. (http://www.arcgis.com/home/item.html?id=dfab3b294ab24961899b2a98e9e8cd3d).

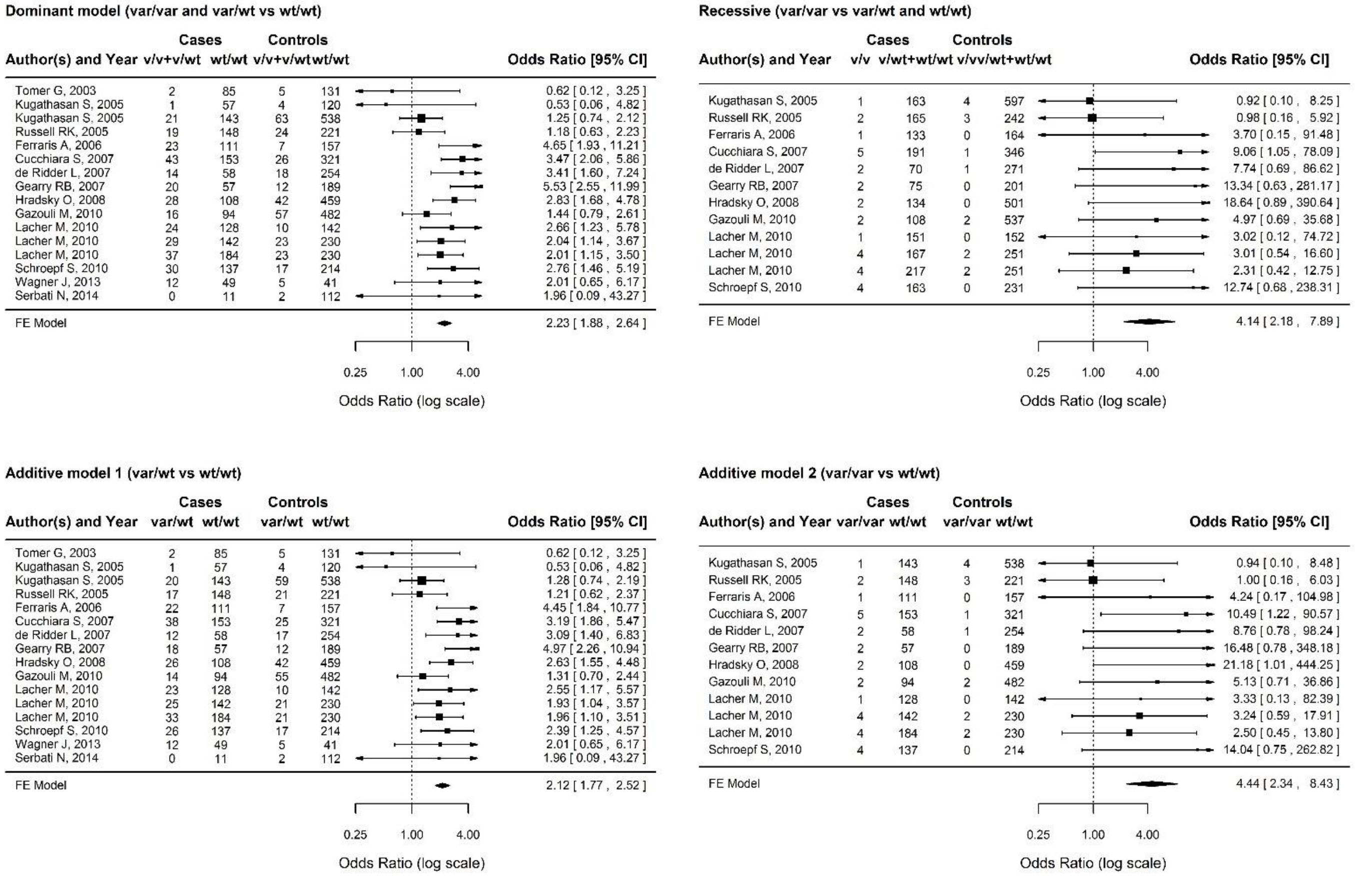

Supplementary Figure S2 Forest plot of rs2066844 in paediatric CD

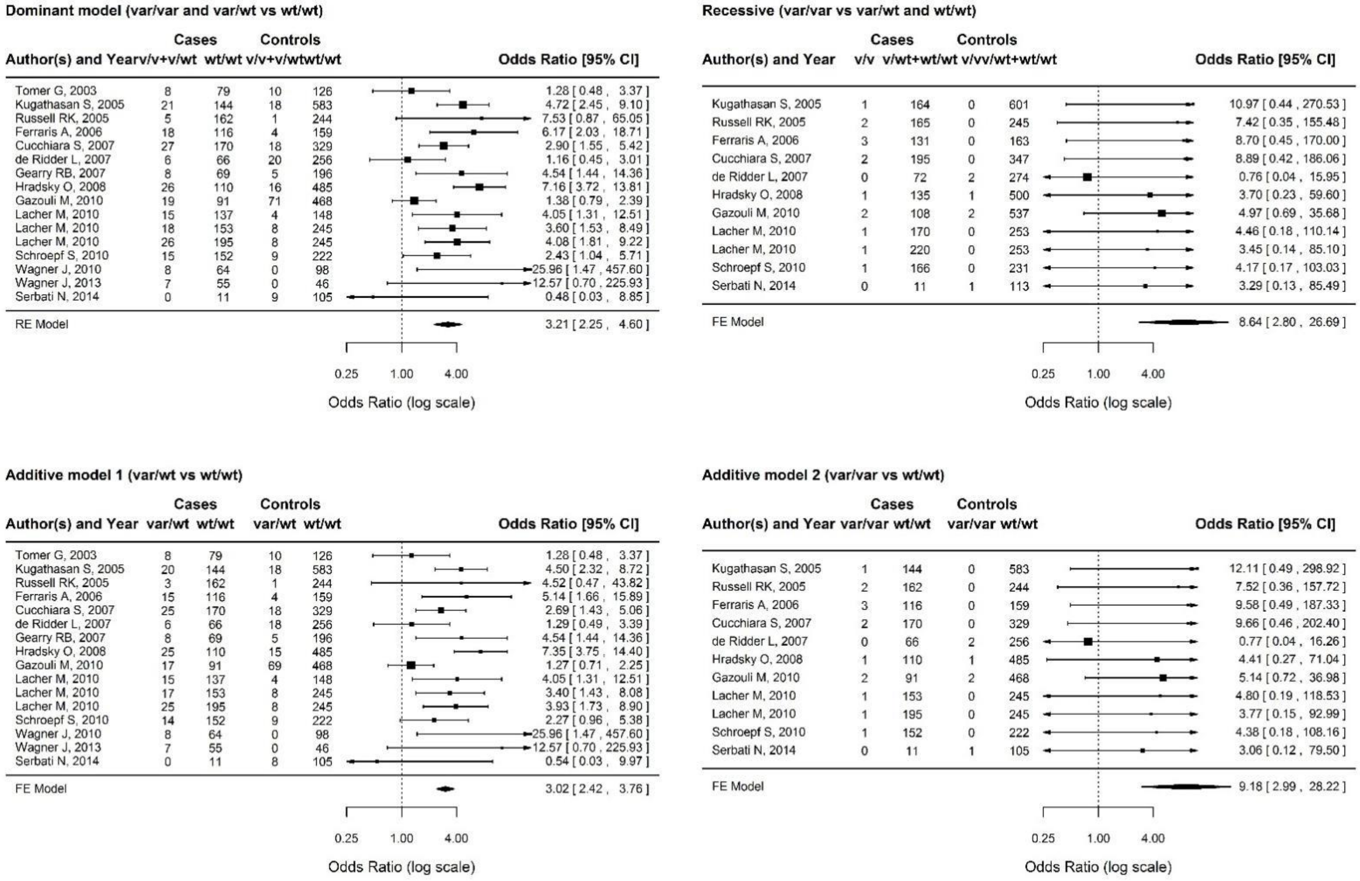

Supplementary Figure S3 Forest plot of rs2066845 in paediatric CD

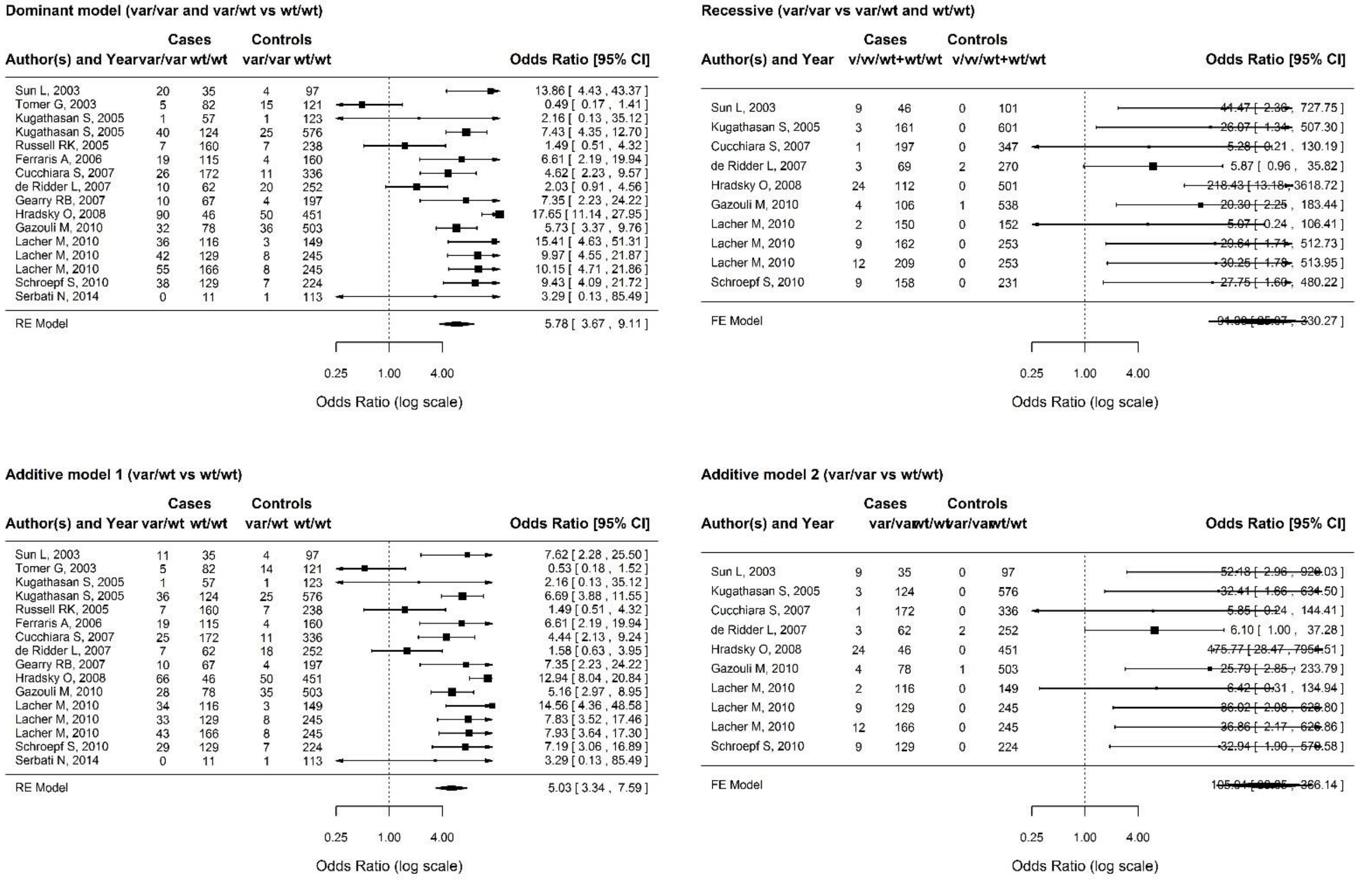

Supplementary Figure S4 Forest plot of rs2066847 in paediatric CD

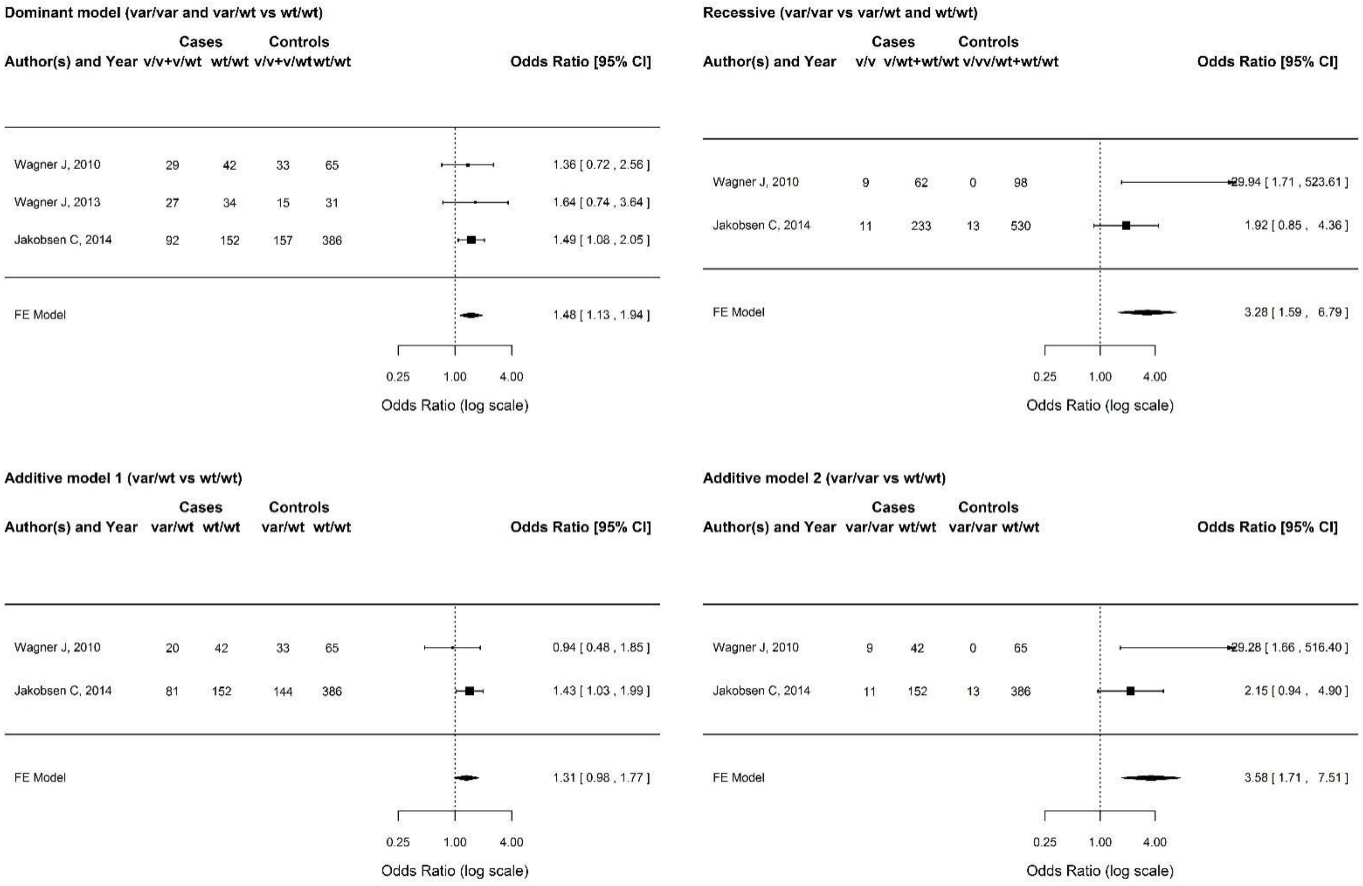

Supplementary Figure S5 Forest plot of rs5743289 in paediatric CD

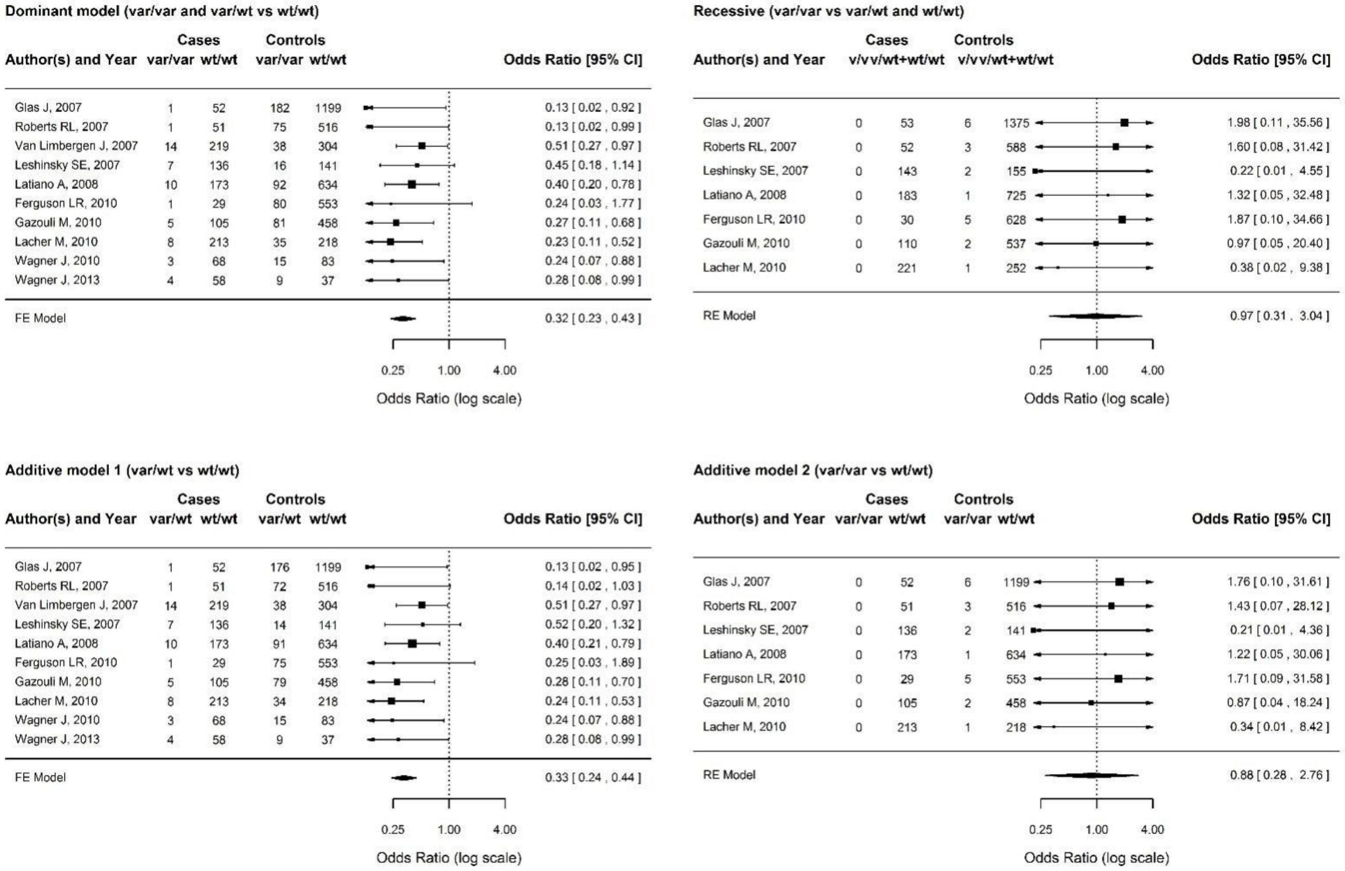

Supplementary Figure S6 Forest plot of rs11209026 in paediatric CD

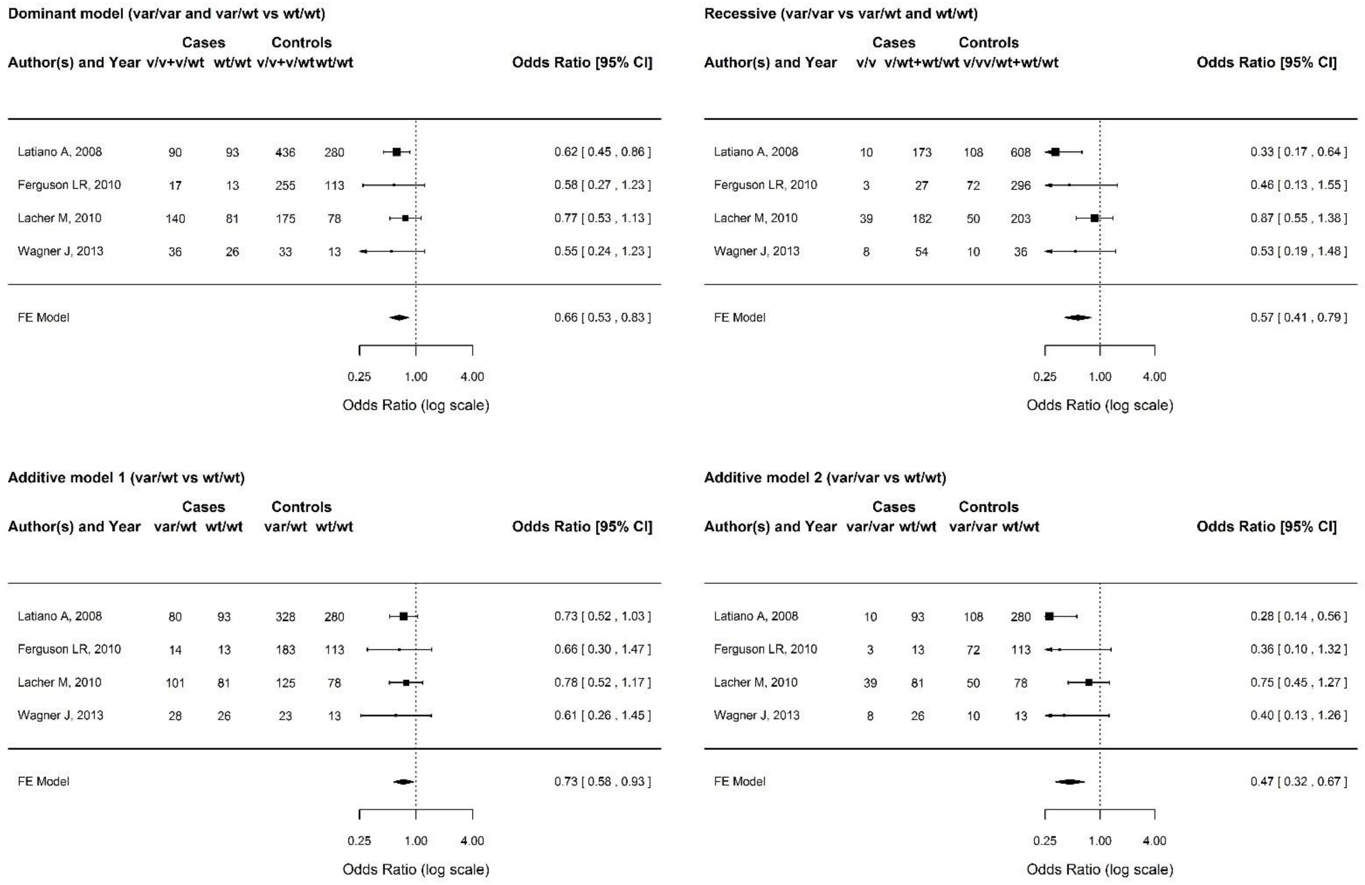

Supplementary Figure S7 Forest plot of rs7517847 in paediatric CD

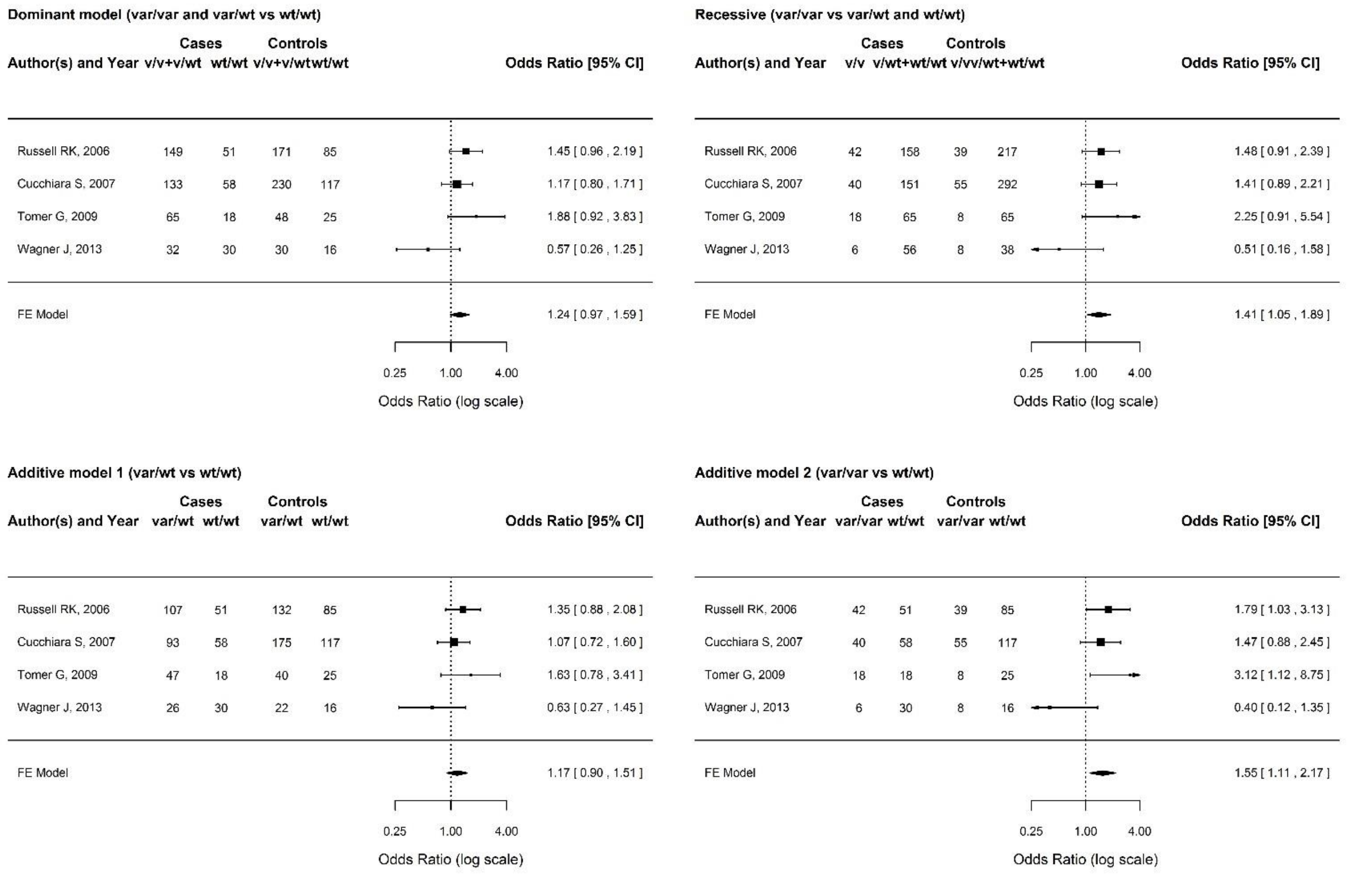

Supplementary Figure S8 Forest plot of rs11739135 in paediatric CD

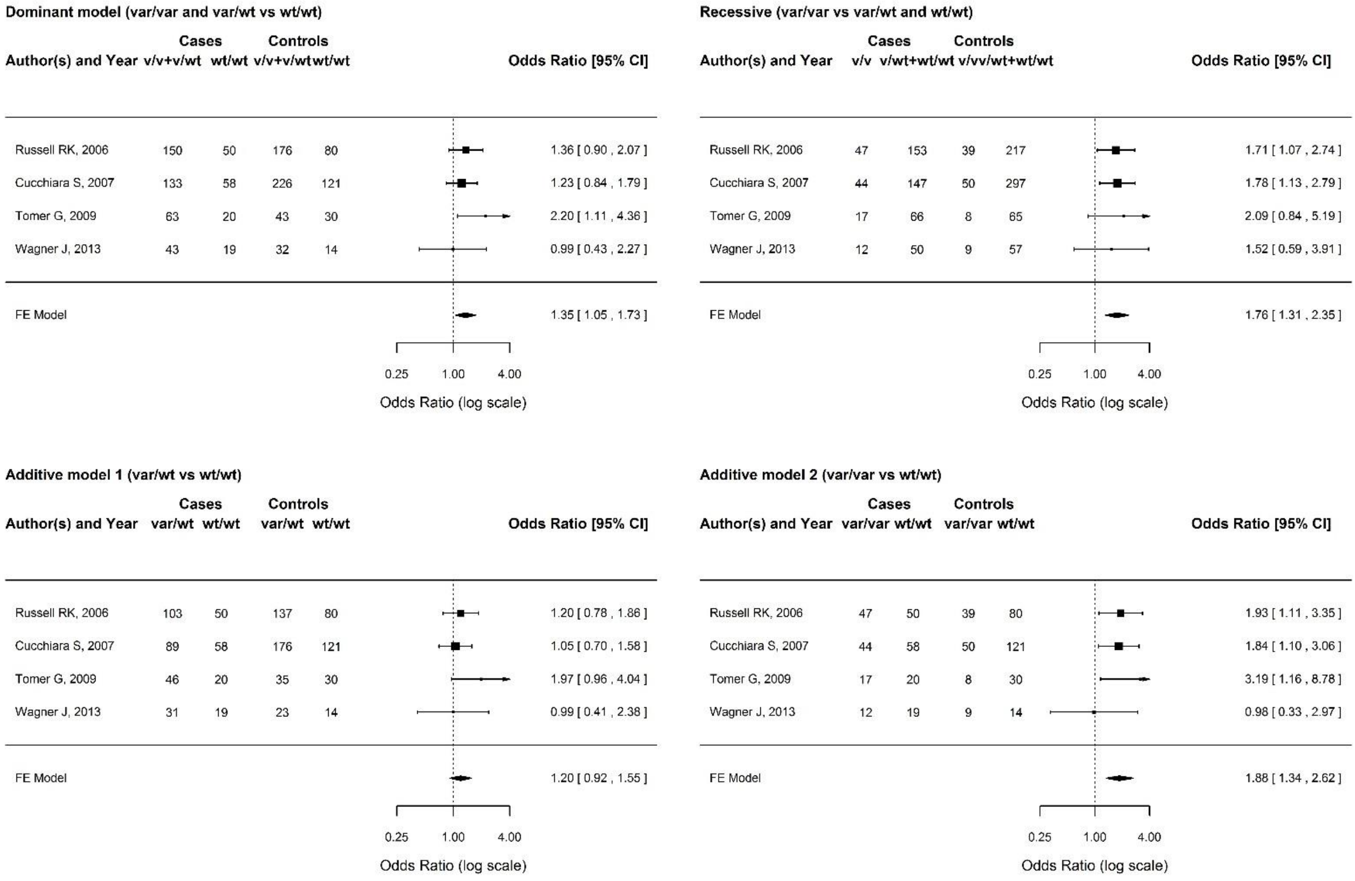

Supplementary Figure S9 Forest plot of rs12521868 in paediatric CD

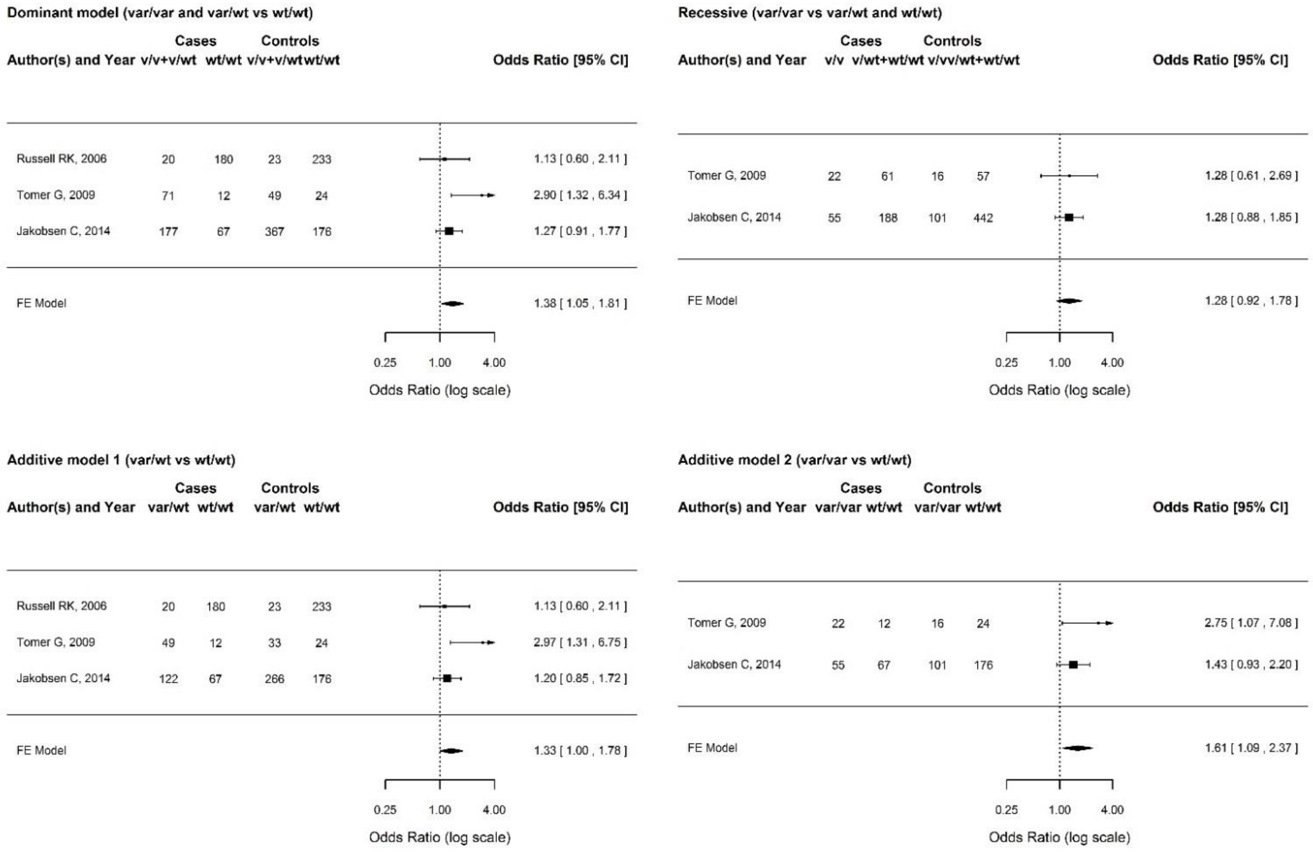

Supplementary Figure S10 Forest plot of rs17622208 in paediatric CD

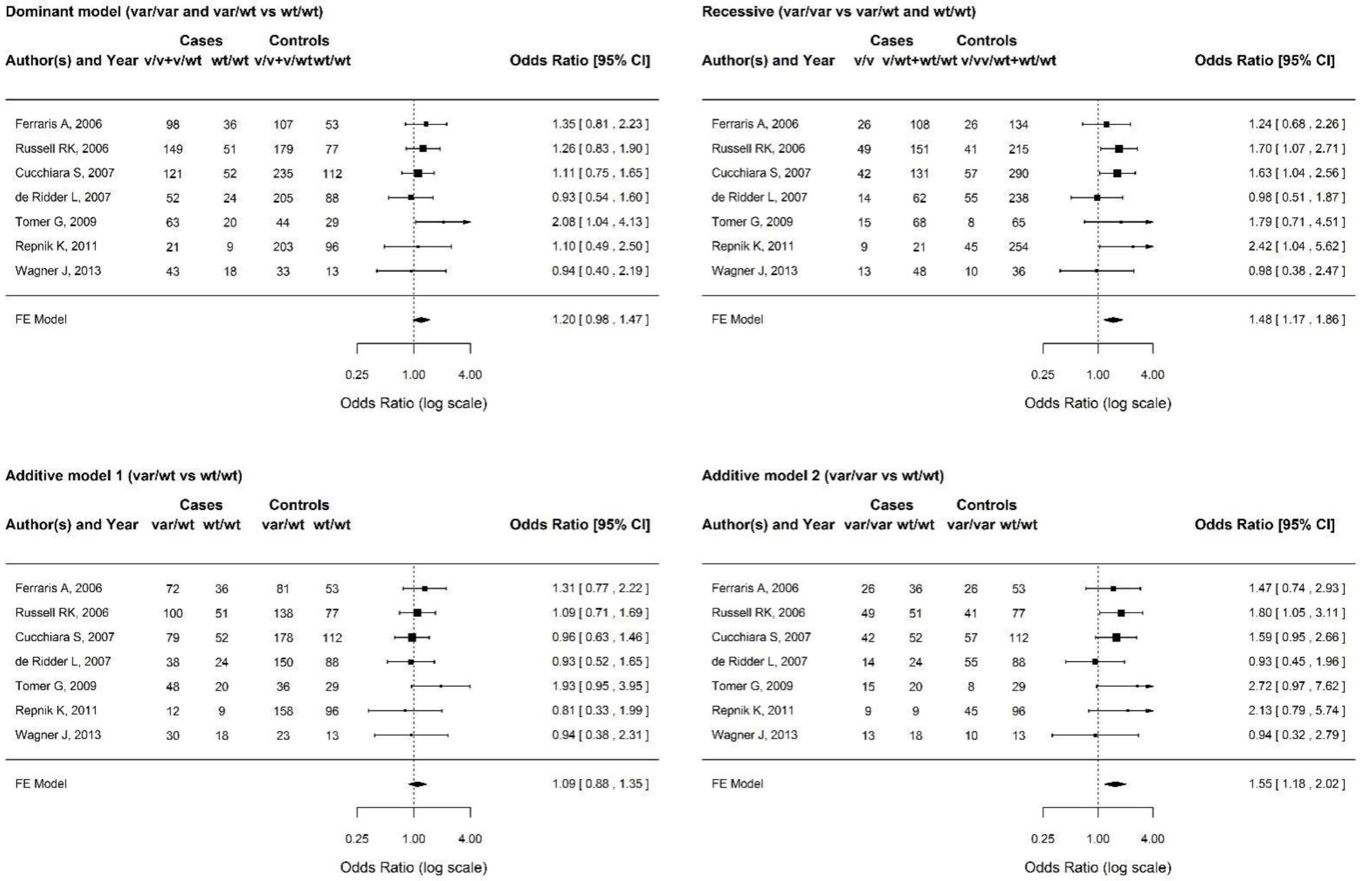

Supplementary Figure S11 Forest plot of rs1050152 in paediatric CD

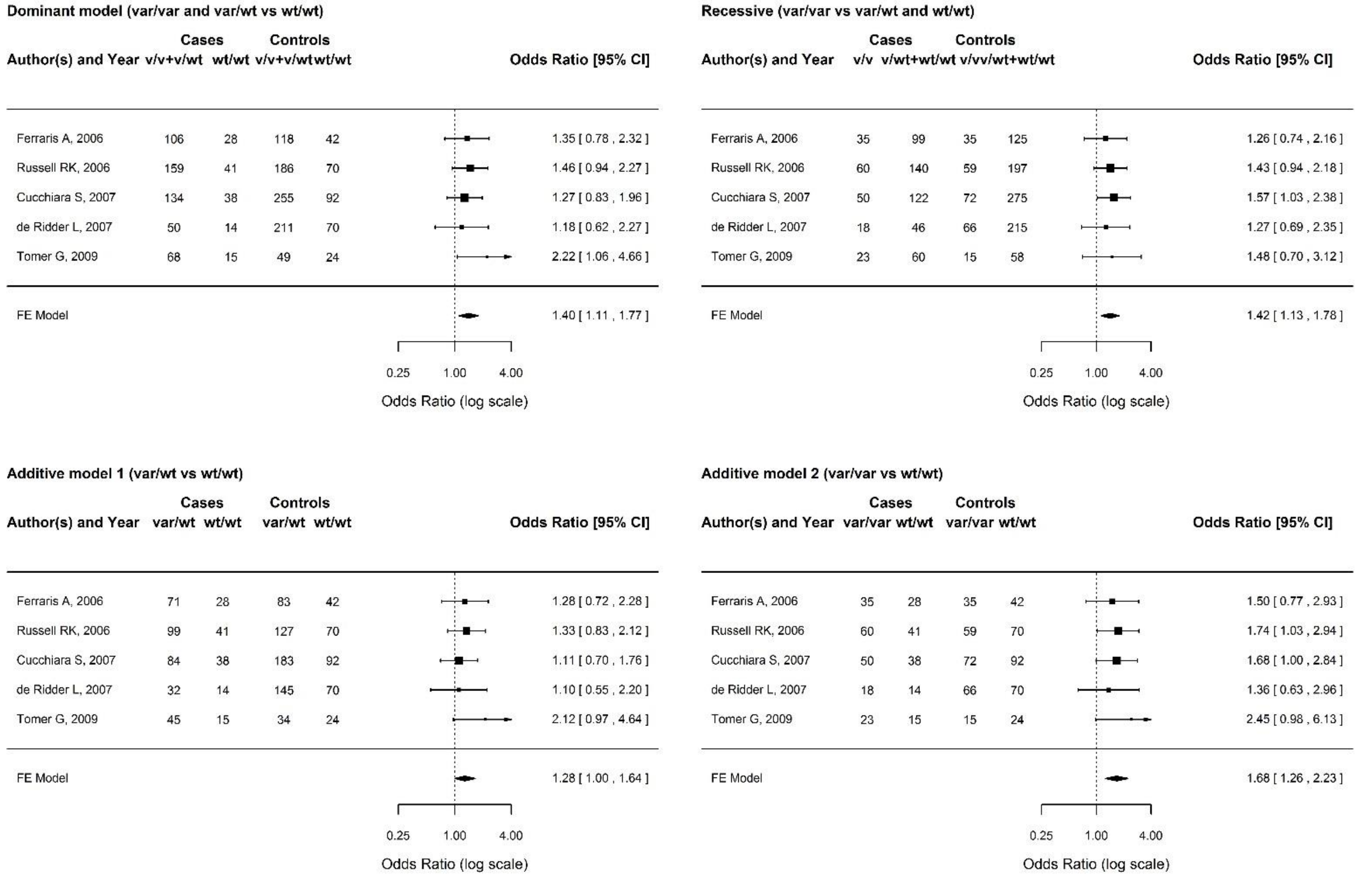

Supplementary Figure S12 Forest plot of rs26313667 in paediatric CD

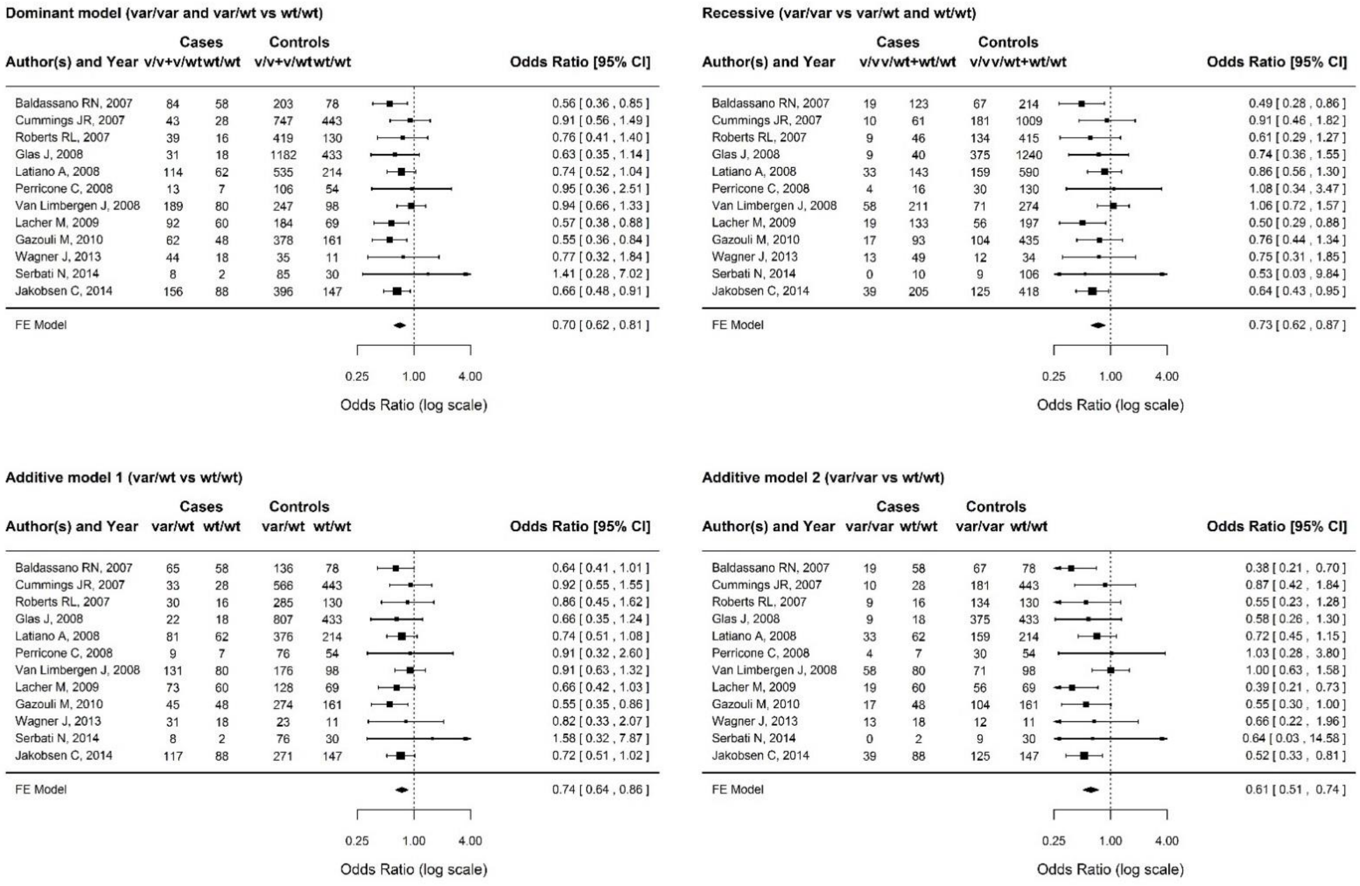

Supplementary Figure S13 Forest plot of rs2241880 in paediatric CD

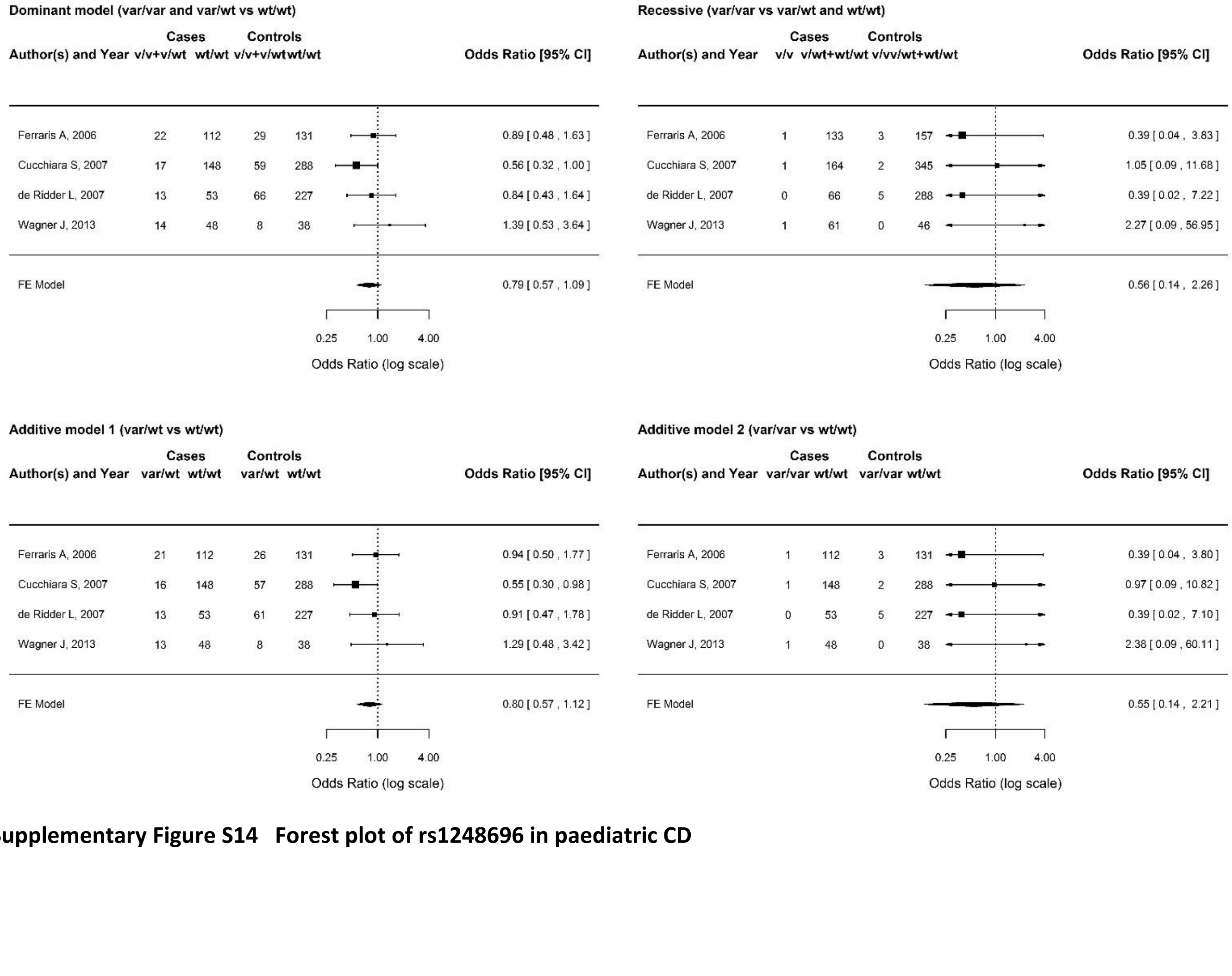

Supplementary Figure S14 Forest plot of rs1248696 in paediatric CD

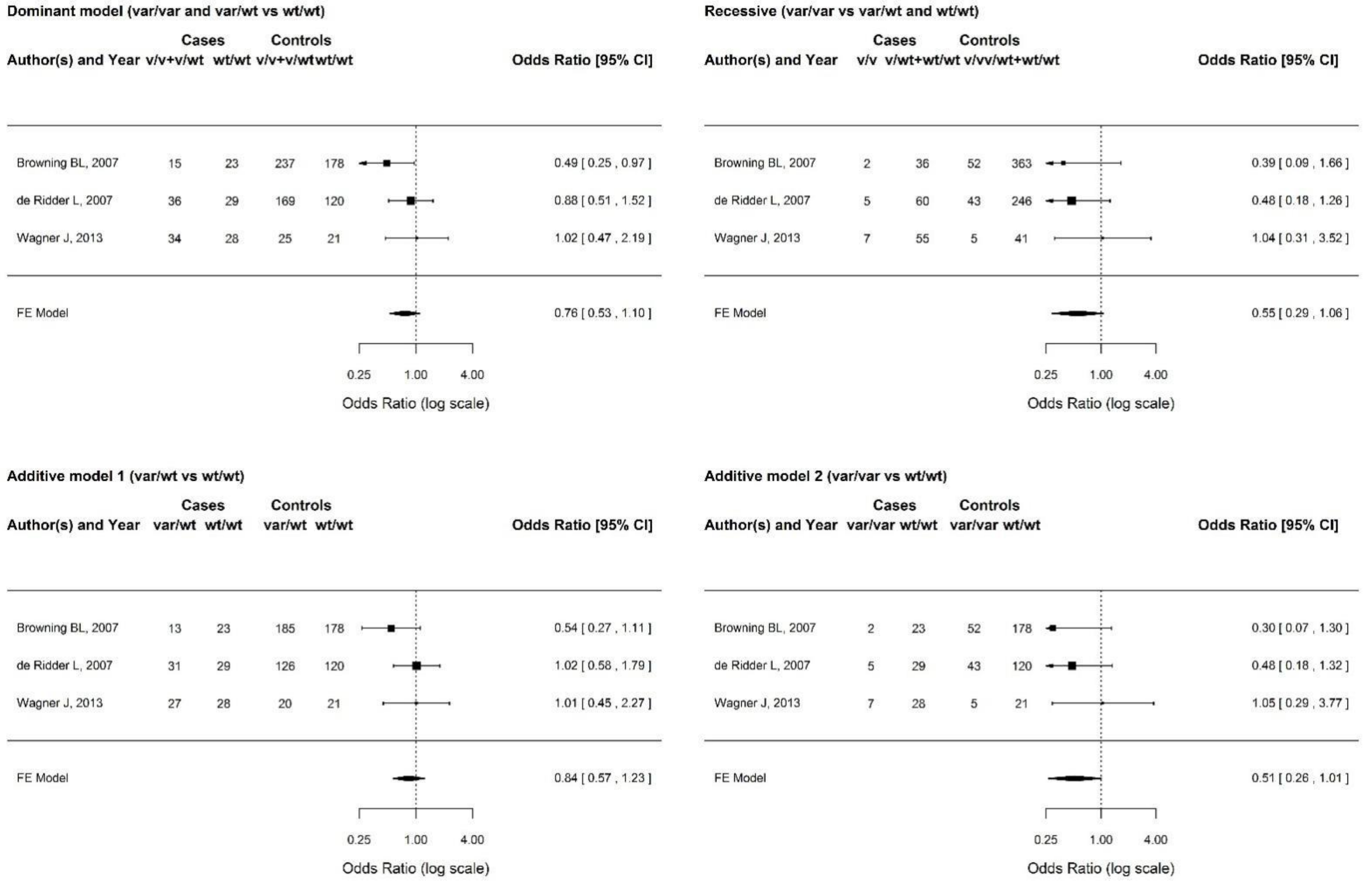

Supplementary Figure S15 Forest plot of rs2289311 in paediatric CD

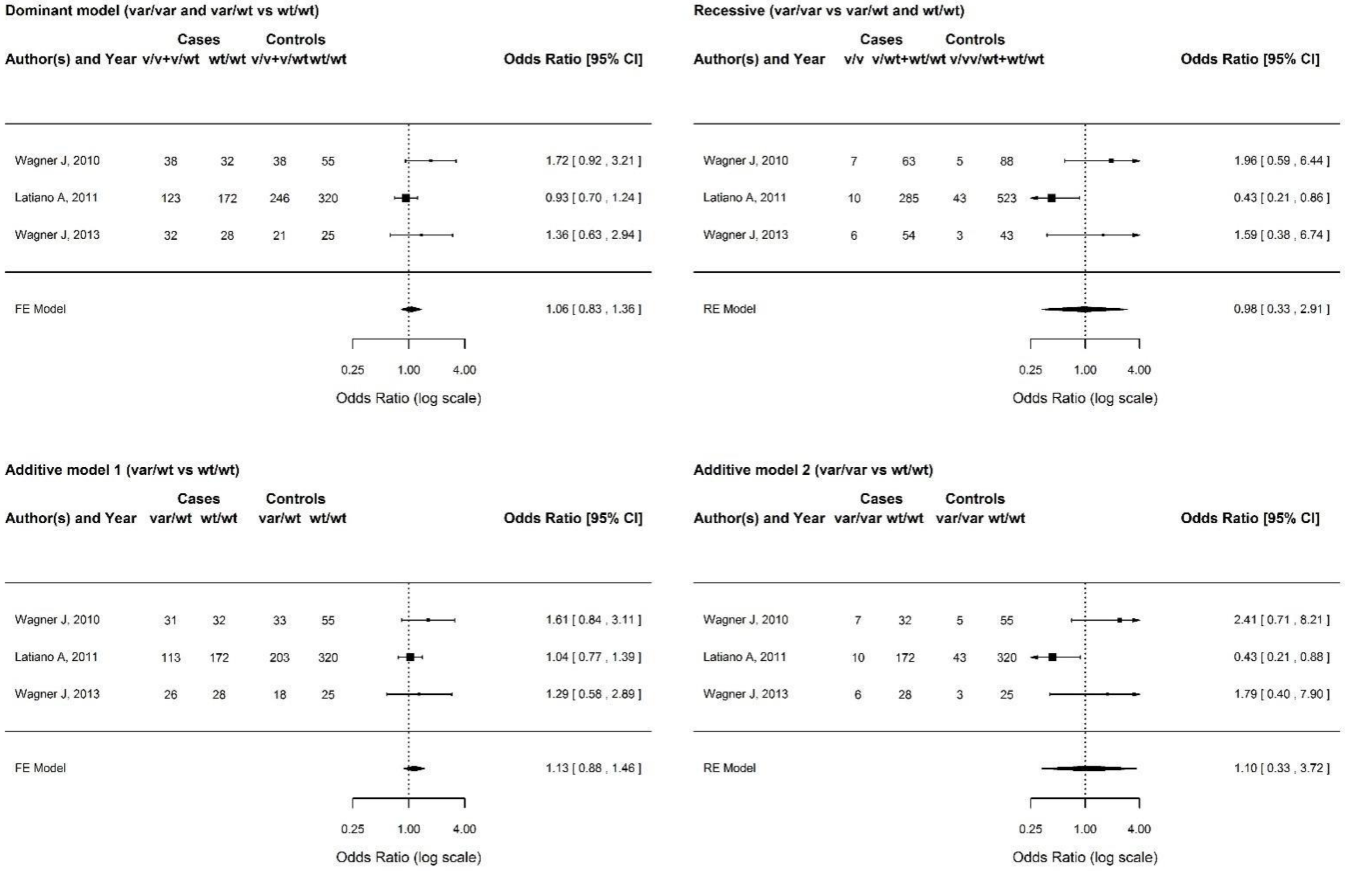

Supplementary Figure S16 Forest plot of rs2836878 in paediatric CD

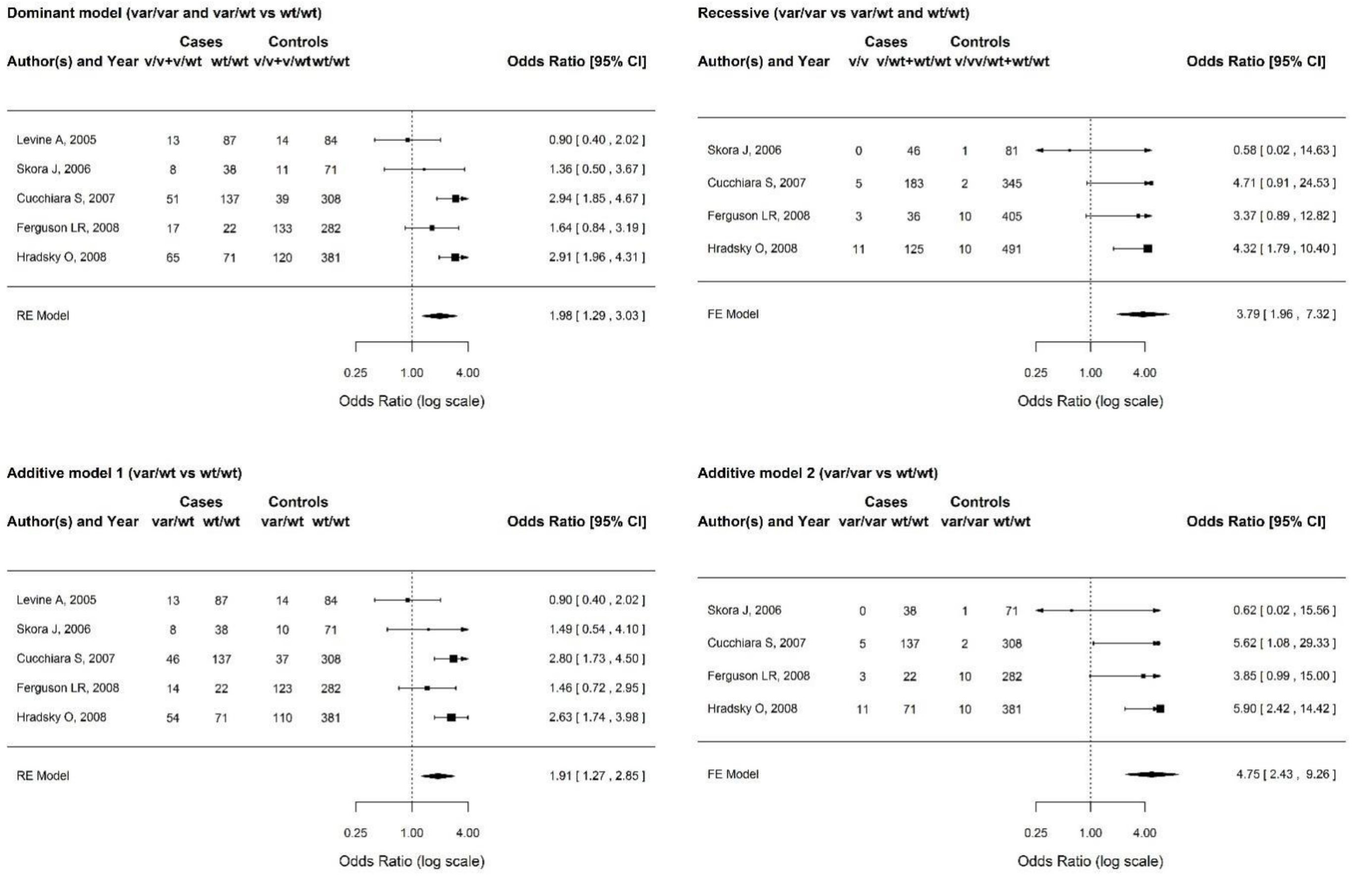

Supplementary Figure S17 Forest plot of rs1800629 in paediatric CD

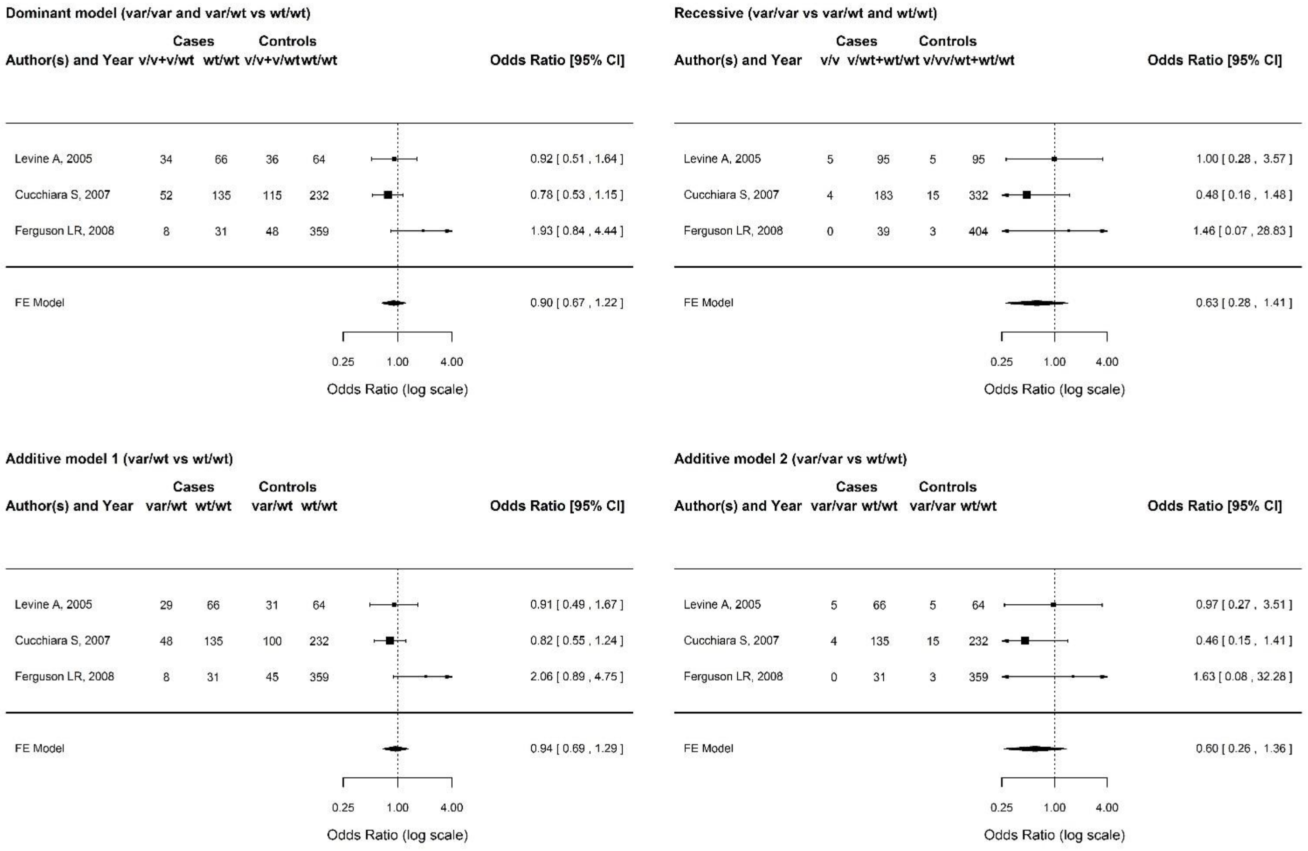

Supplementary Figure S18 Forest plot of rs1799724 in paediatric CD

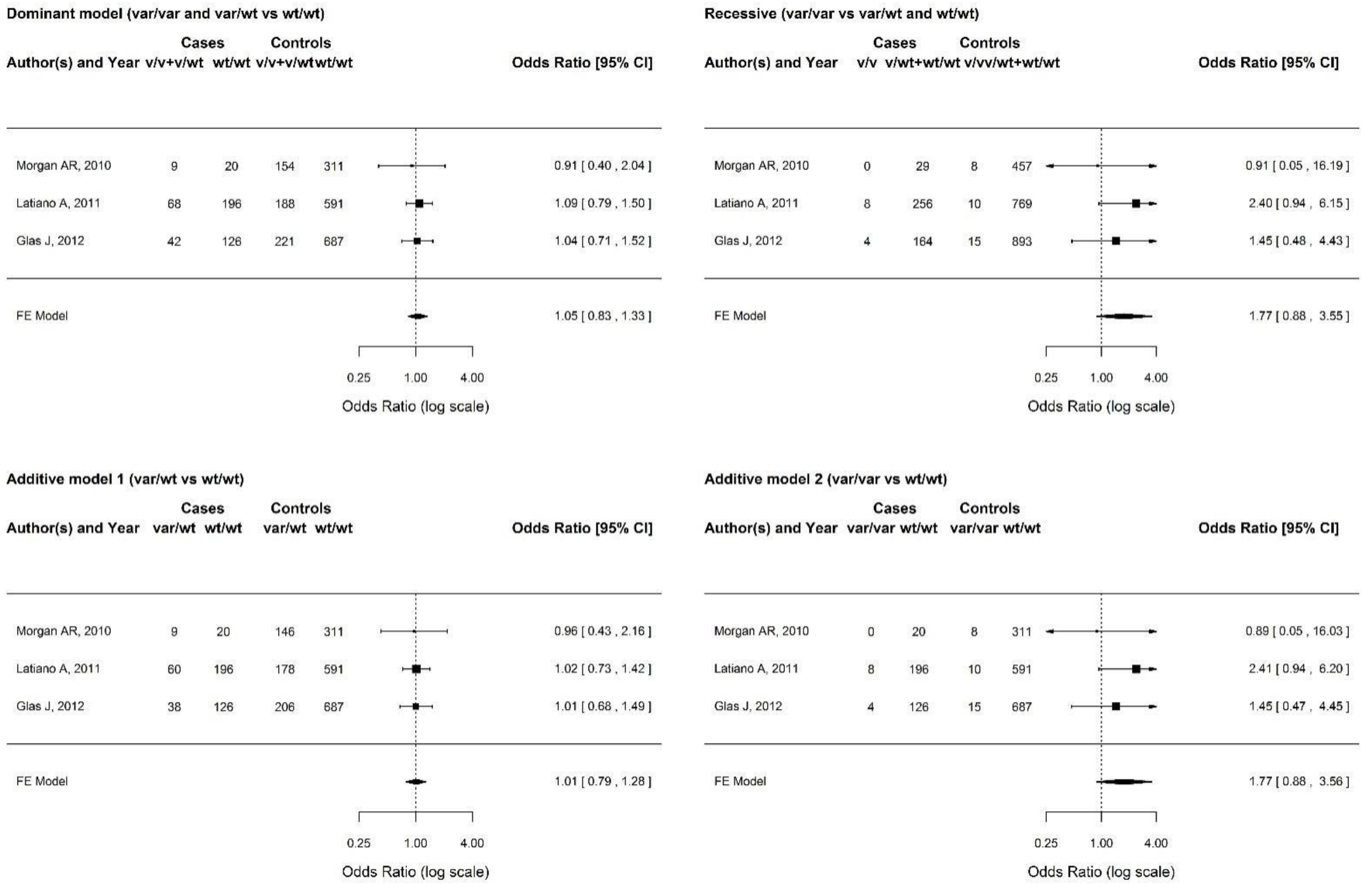

Supplementary Figure S19 Forest plot of rs2542151 in paediatric CD

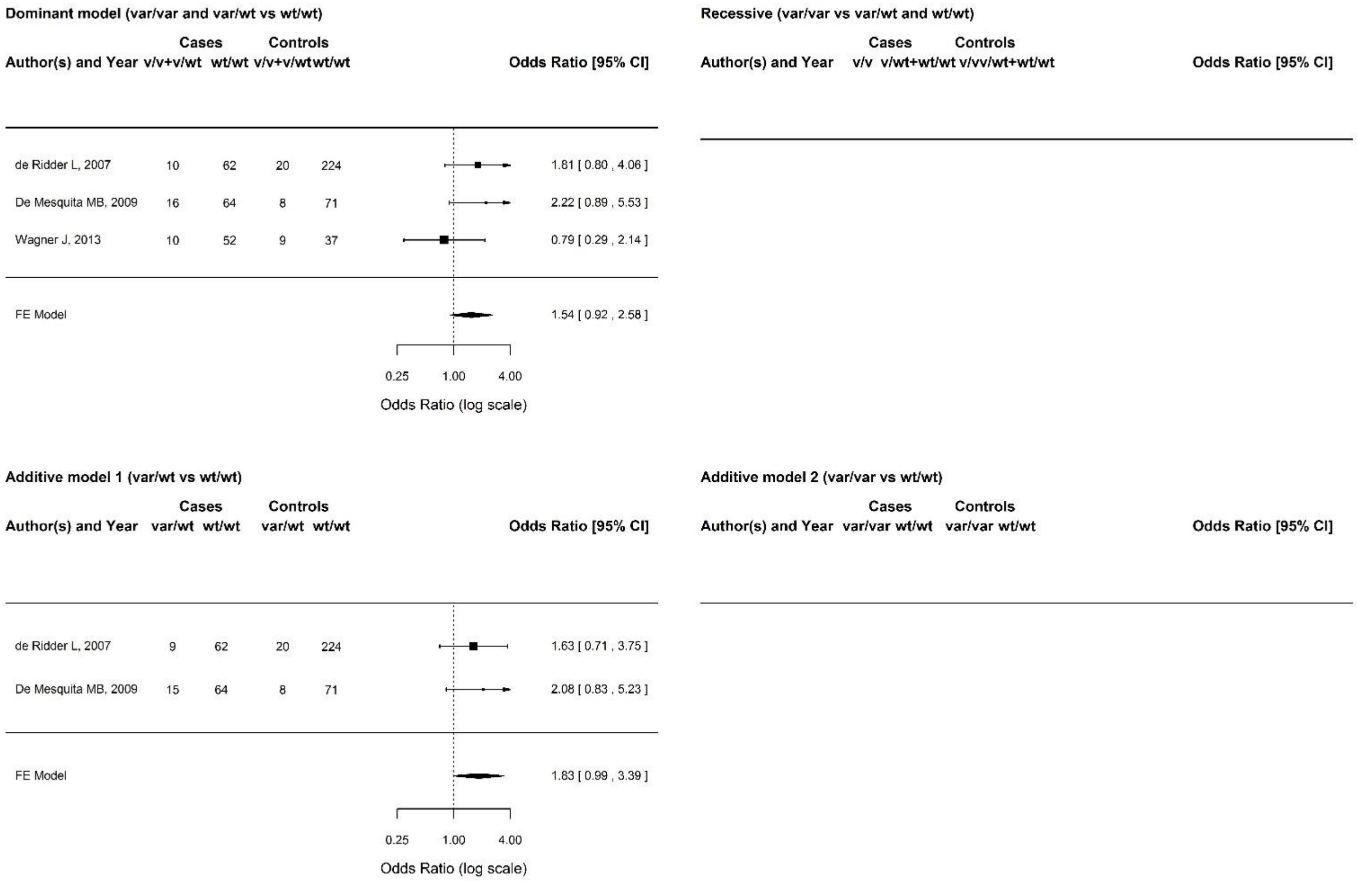

Supplementary Figure S20 Forest plot of rs4986790 in paediatric CD

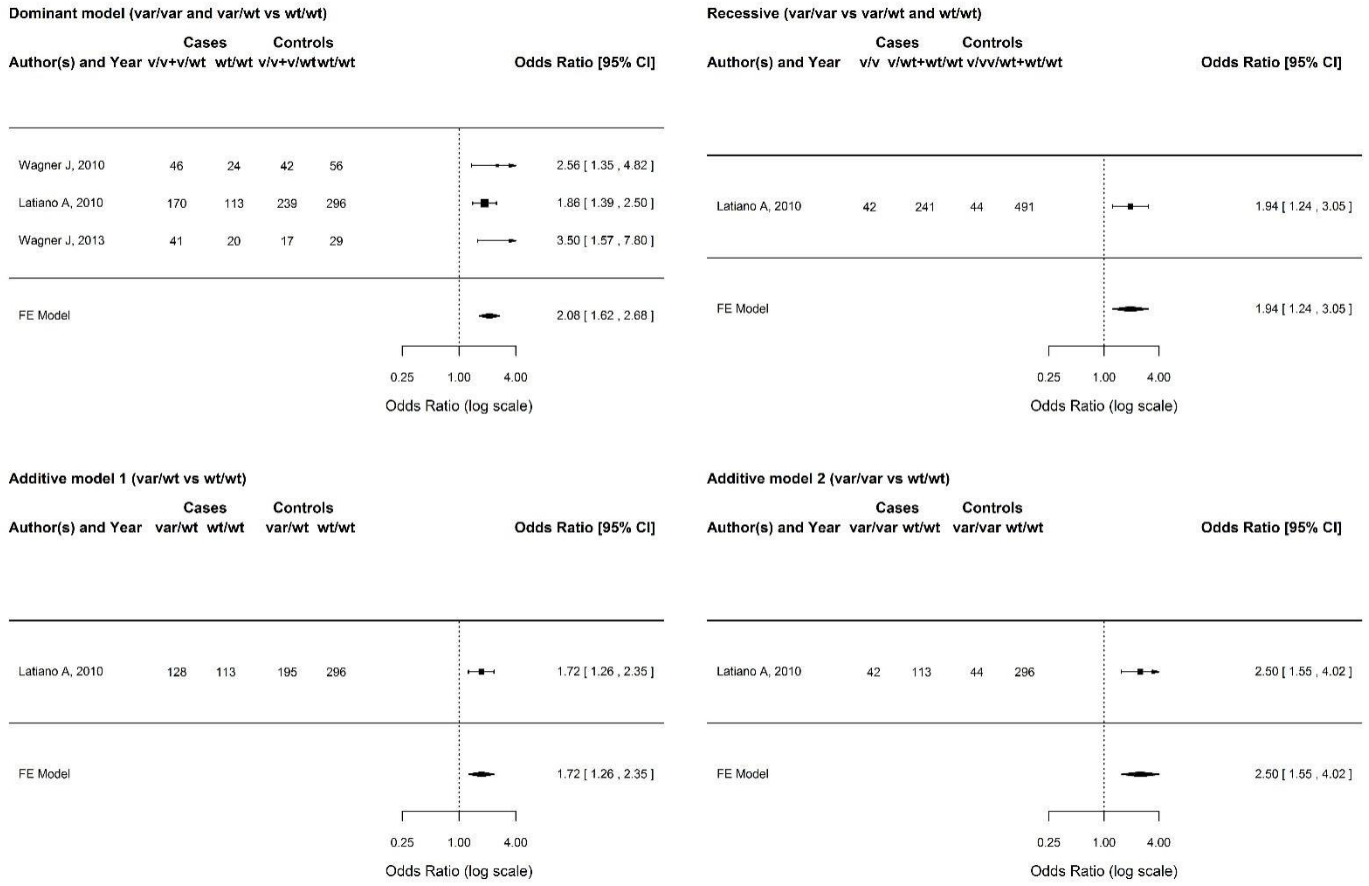

Supplementary Figure S21 Forest plot of rs9858542 in paediatric CD

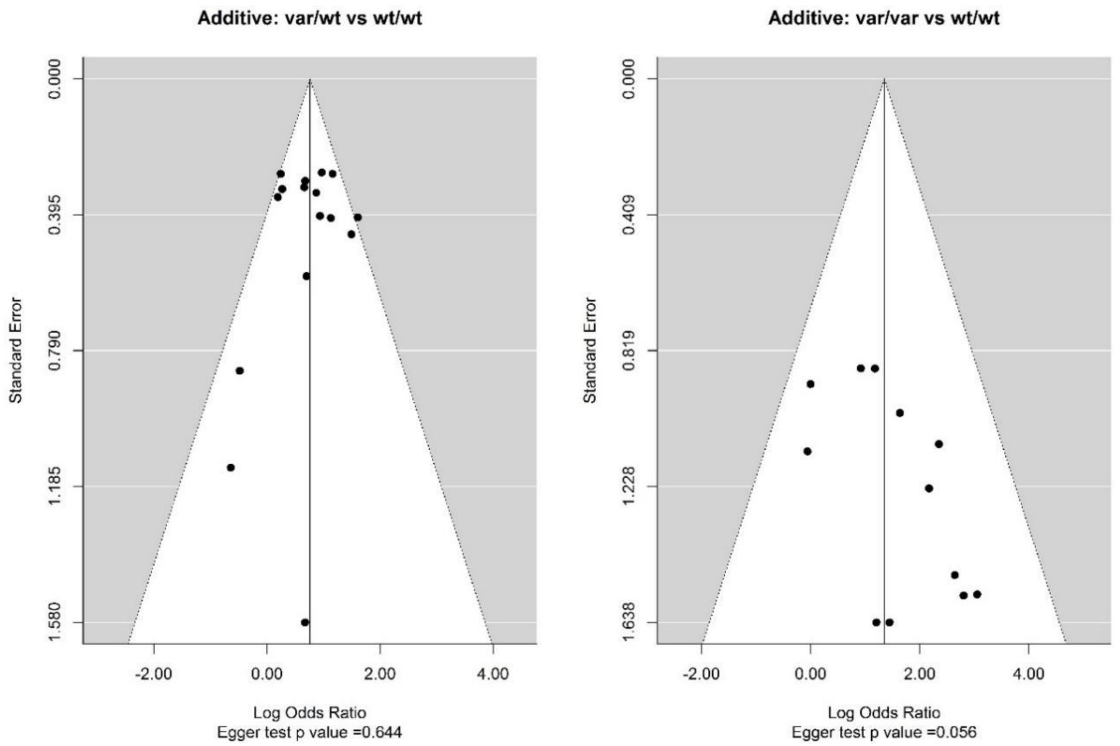

Supplementary Figure S22 Funnel plot with Egger test of rs2066844 in paediatric CD

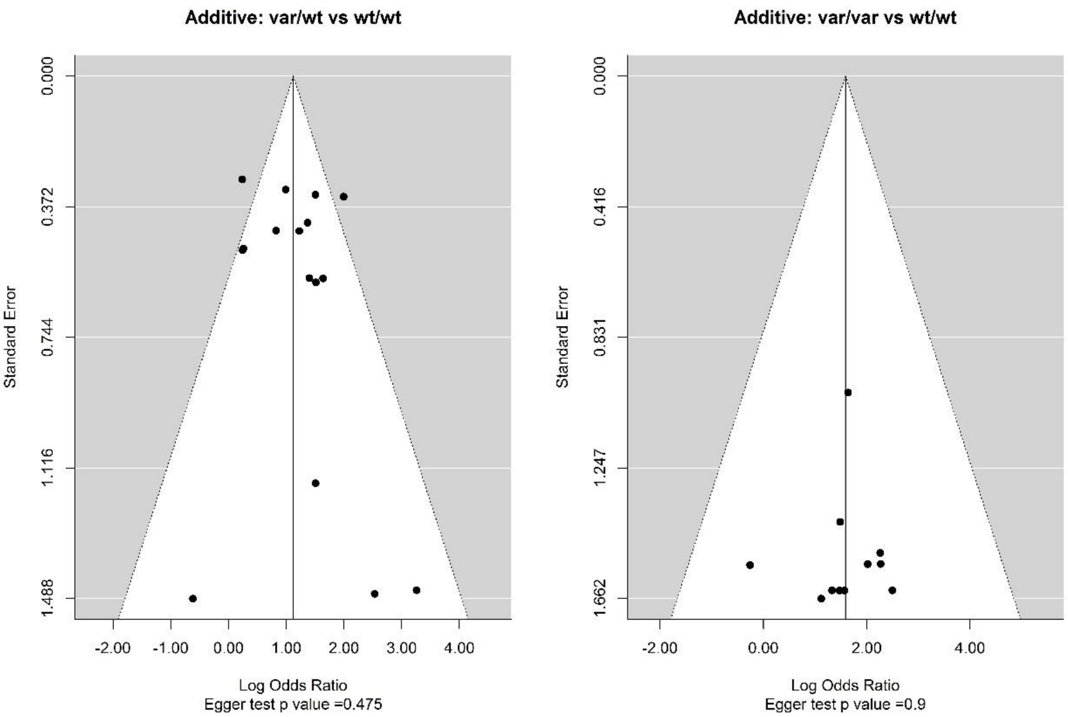

Supplementary Figure S23 Funnel plot with Egger test of rs2066845 in paediatric CD

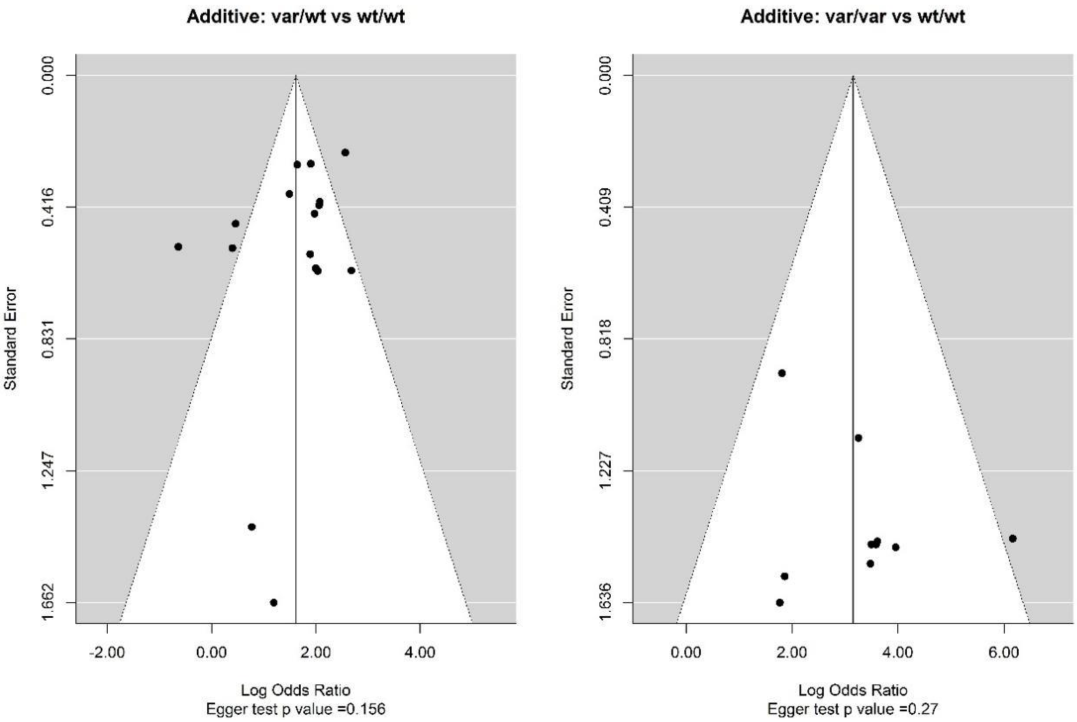

Supplementary Figure S24 Funnel plot with Egger test of rs2066847 in paediatric CD

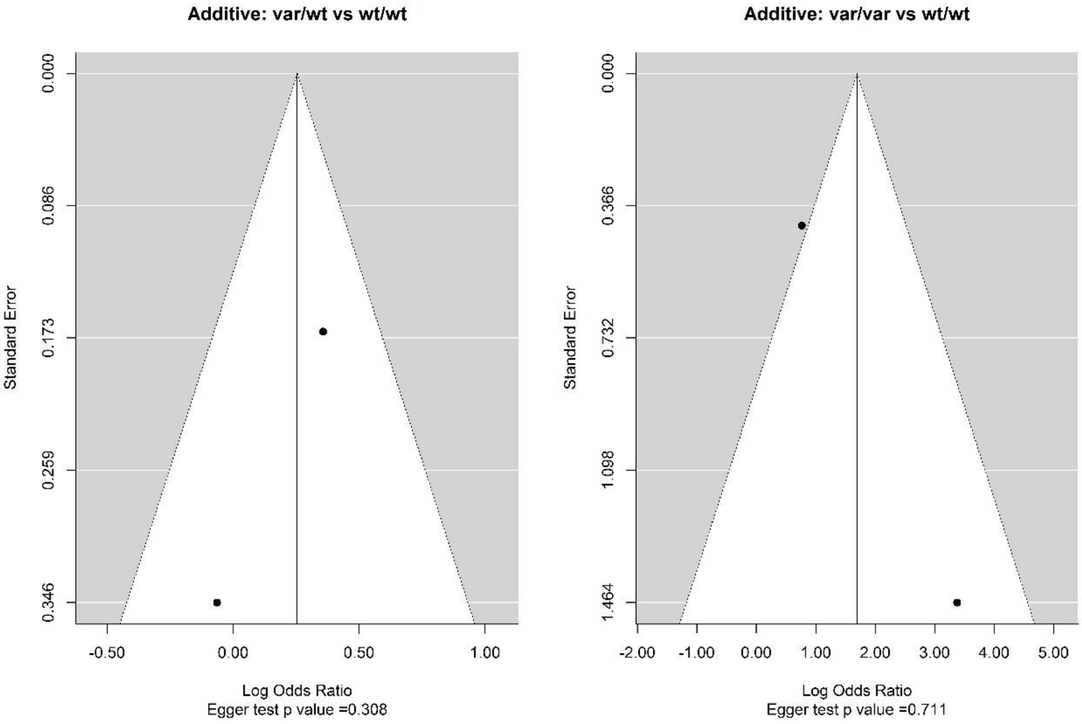

Supplementary Figure S25 Funnel plot with Egger test of rs5743289 in paediatric CD

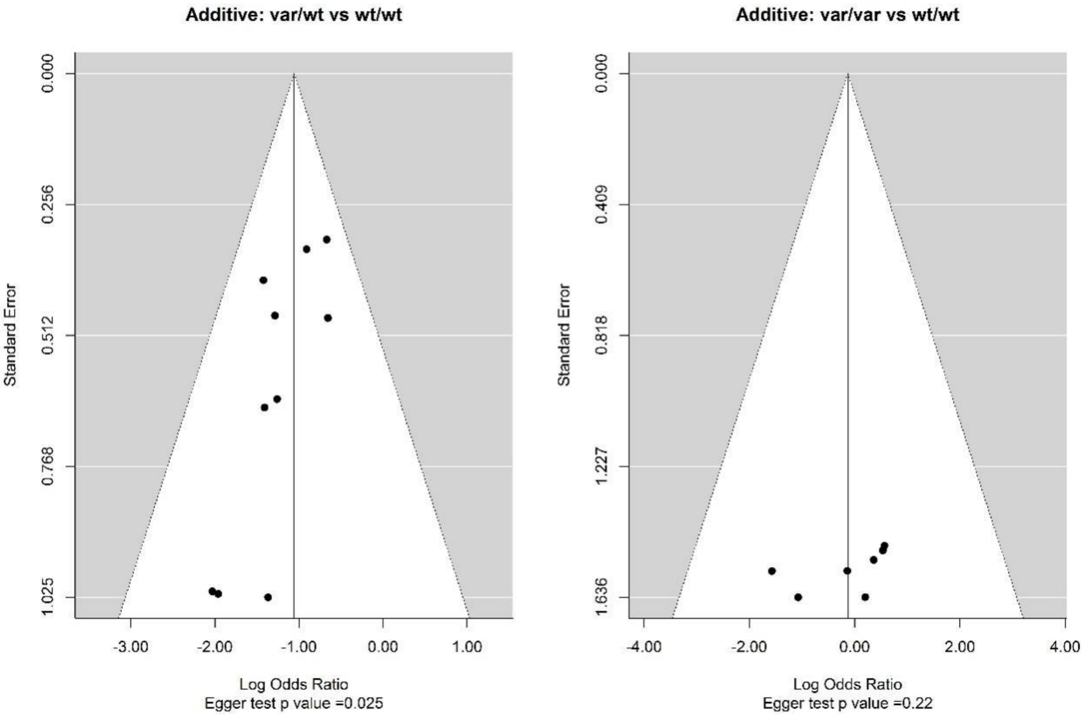

Supplementary Figure S26 Funnel plot with Egger test of rs11209026 in paediatric CD

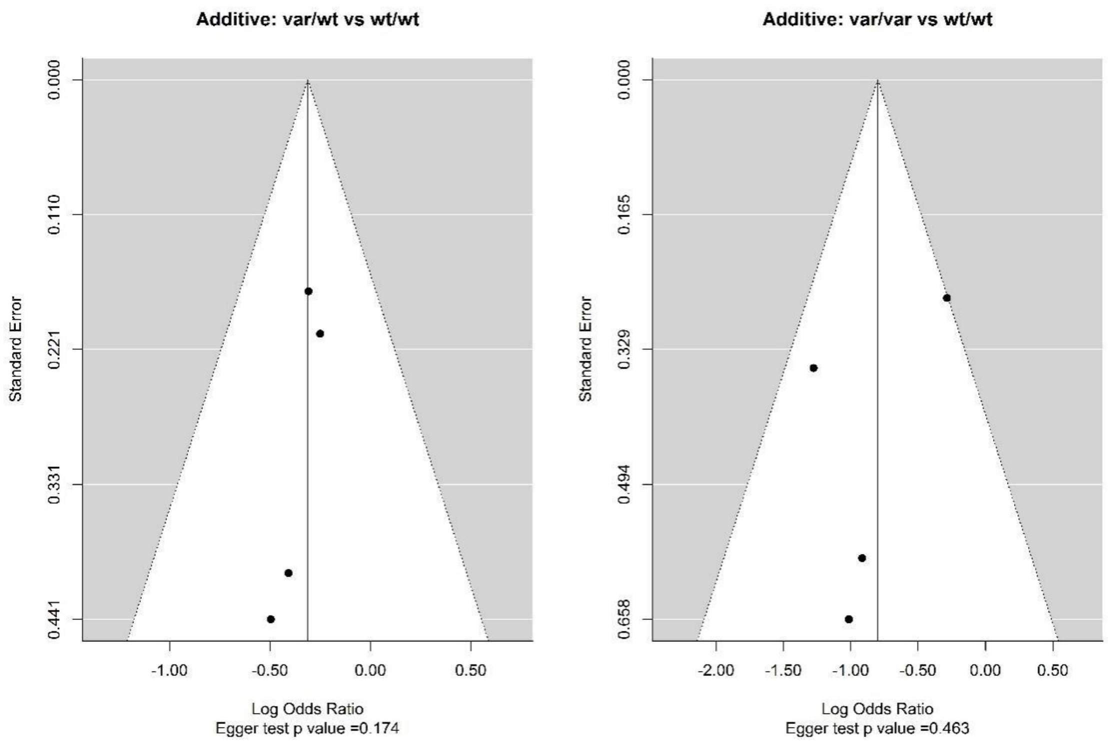

Supplementary Figure S27 Funnel plot with Egger test of rs7517847 in paediatric CD

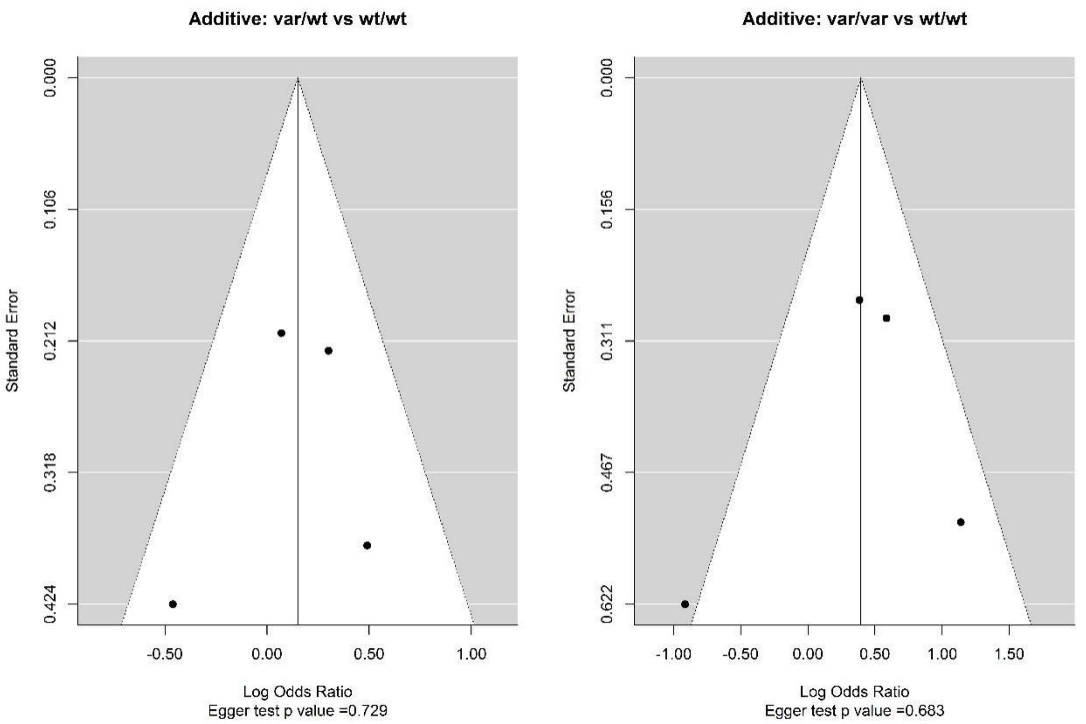

Supplementary Figure S28 Funnel plot with Egger test of rs11739135 in paediatric CD

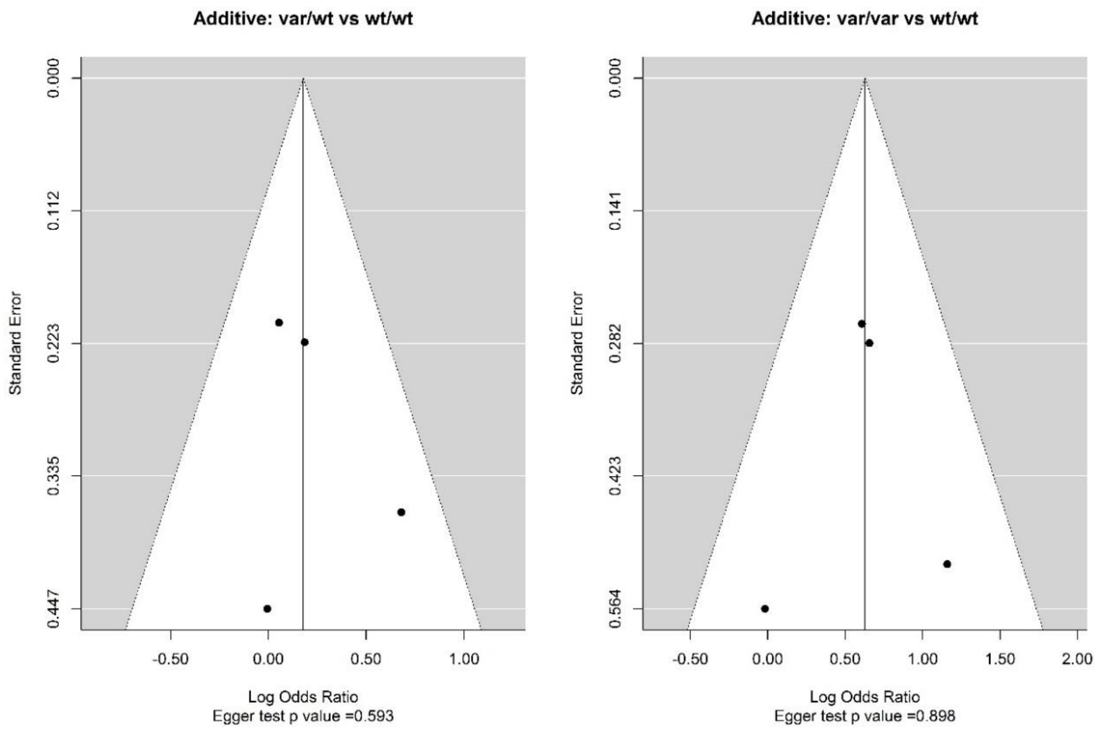

Supplementary Figure S29 Funnel plot with Egger test of rs12521868 in paediatric CD

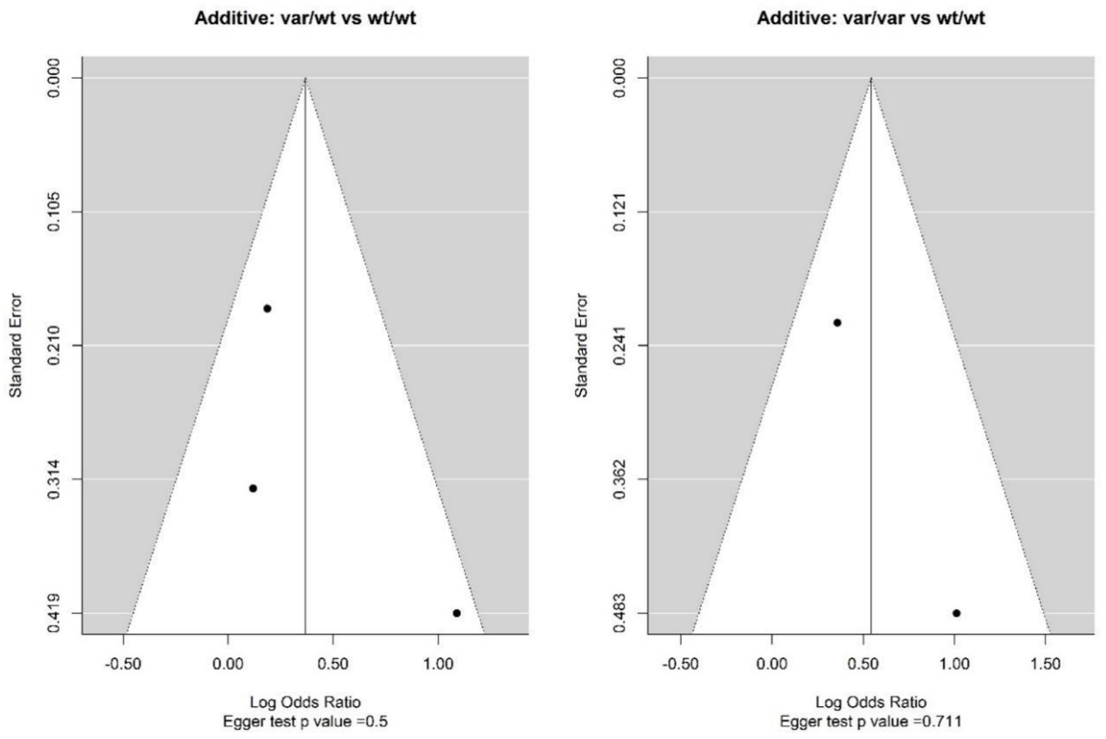

Supplementary Figure S30 Funnel plot with Egger test of rs17622208 in paediatric CD

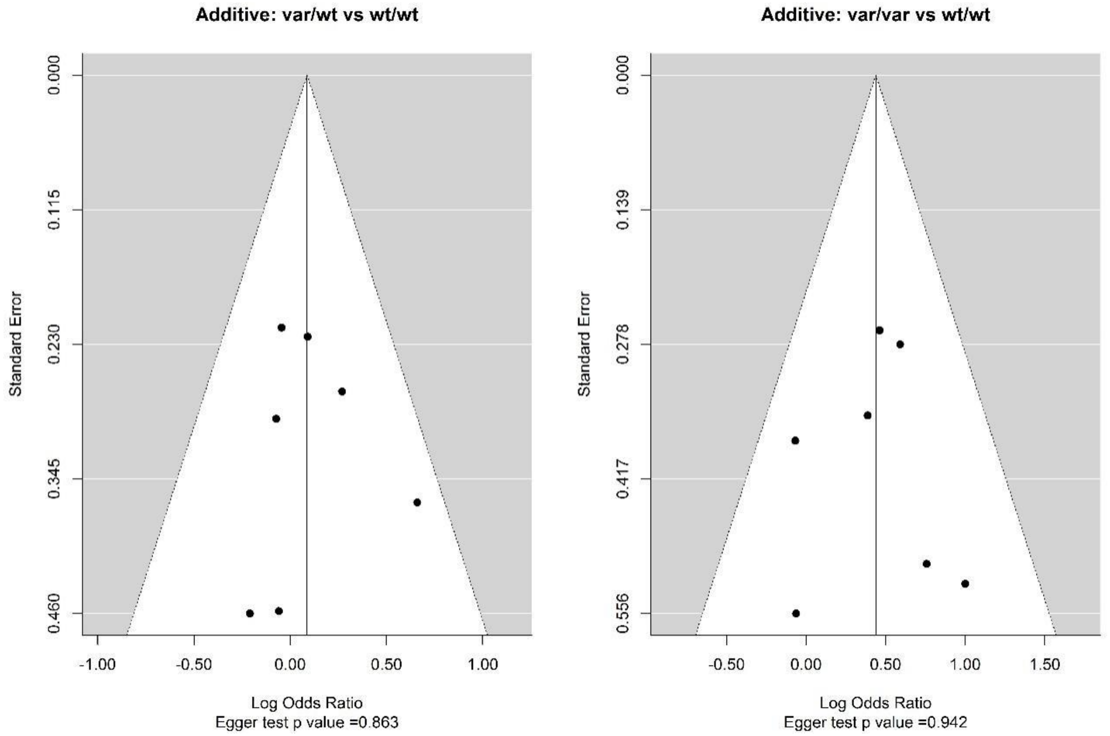

Supplementary Figure S31 Funnel plot with Egger test of rs1050152 in paediatric CD

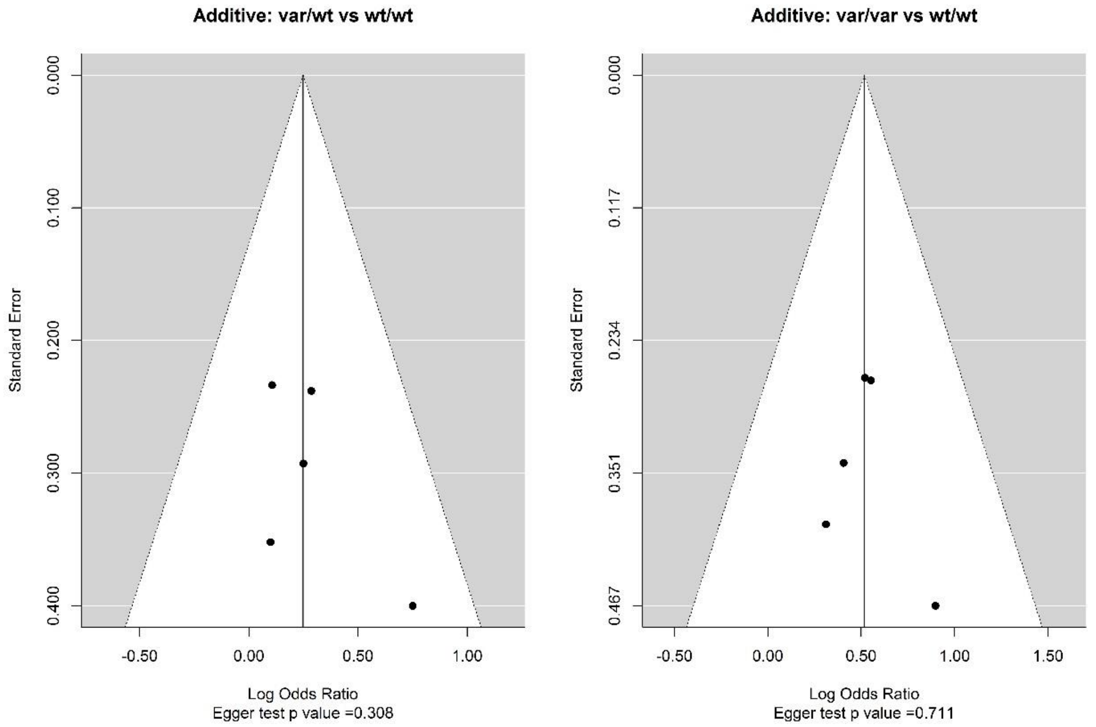

Supplementary Figure S32 Funnel plot with Egger test of rs26313667 in paediatric CD

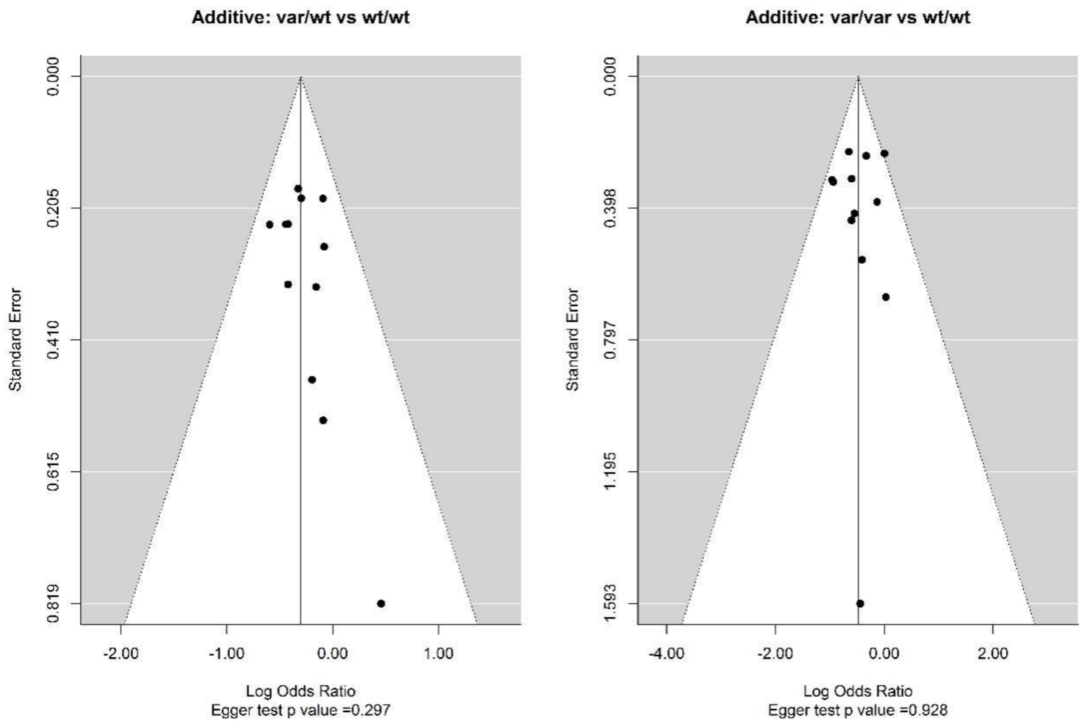

Supplementary Figure S33 Funnel plot with Egger test of rs2241880 in paediatric CD

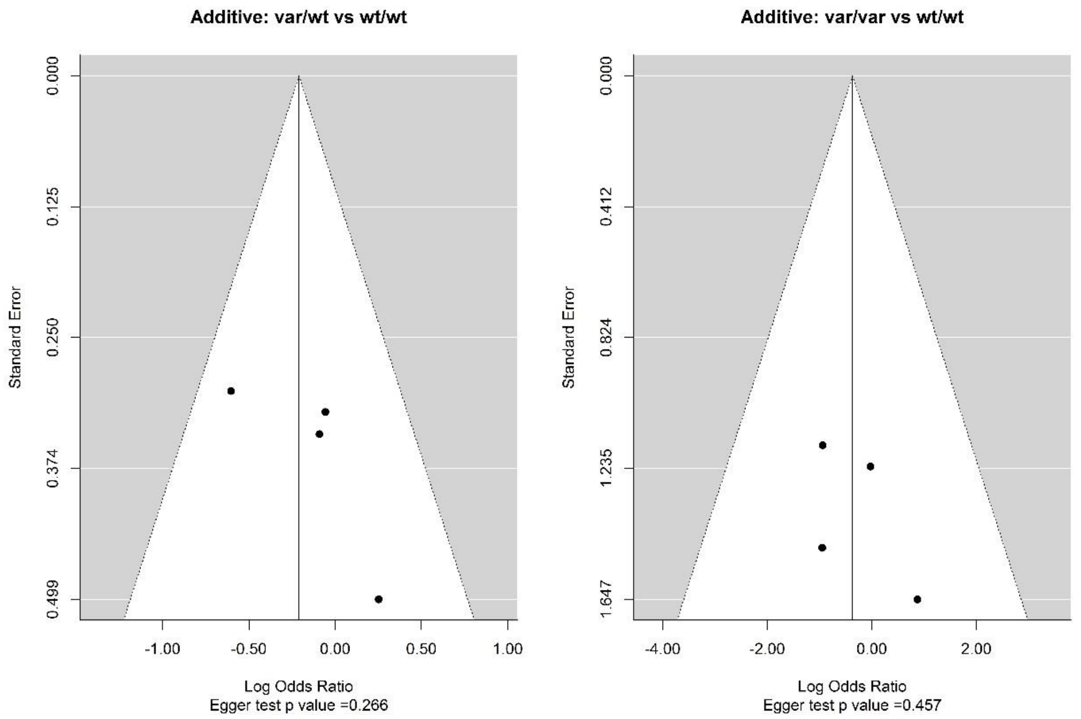

Supplementary Figure S34 Funnel plot with Egger test of rs1248696 in paediatric CD

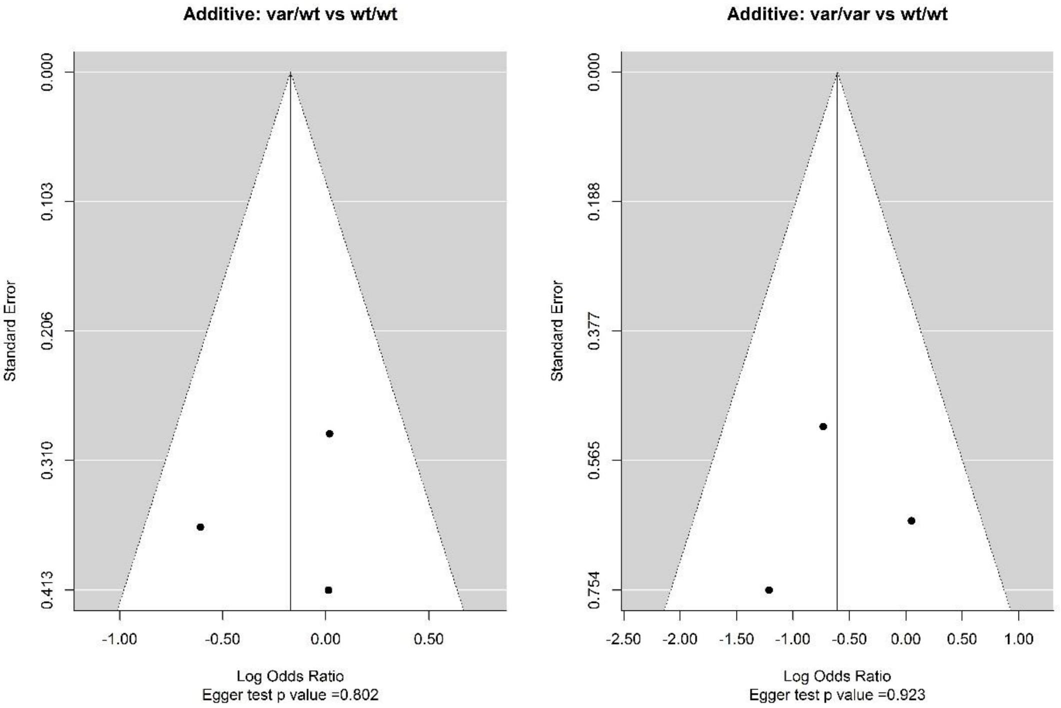

Supplementary Figure S35 Funnel plot with Egger test of rs2289311 in paediatric CD

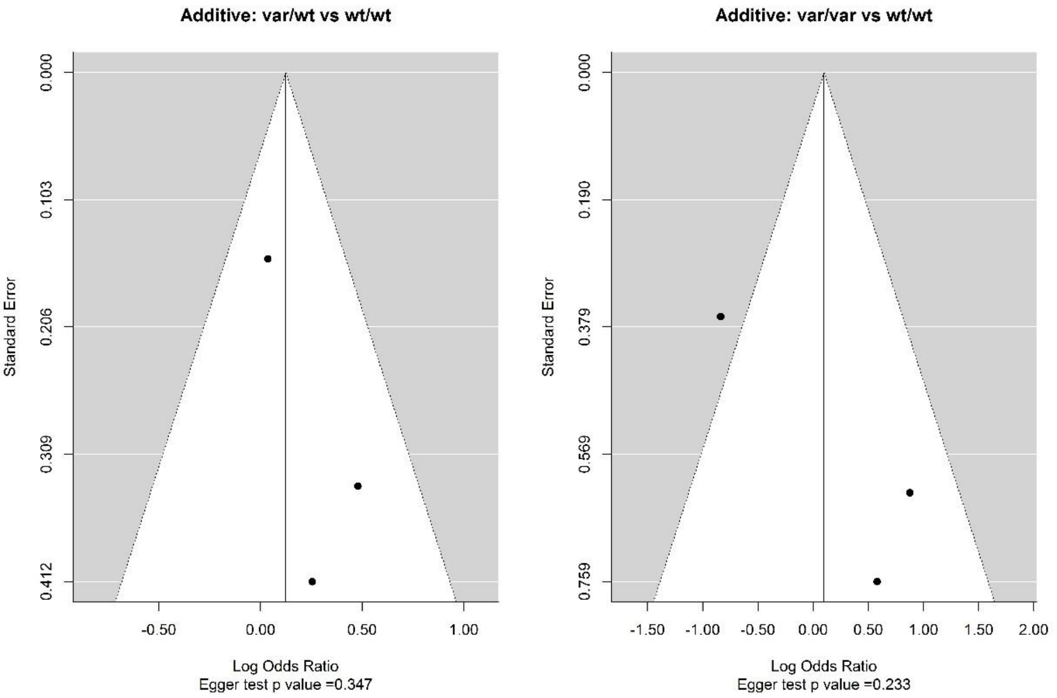

Supplementary Figure S36 Funnel plot with Egger test of rs2836878 in paediatric CD

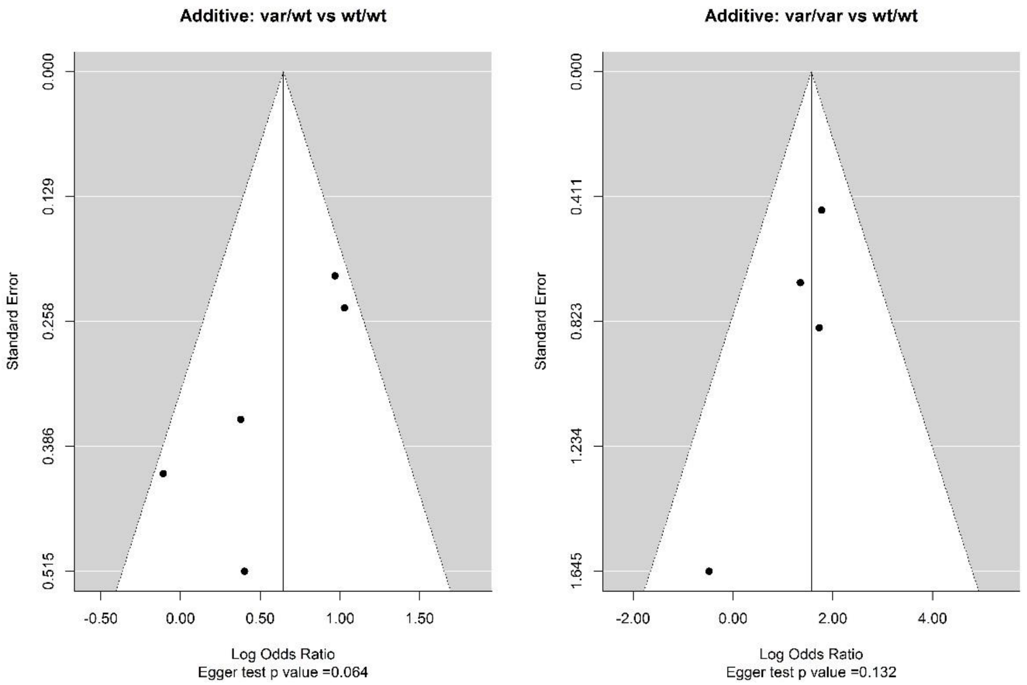

Supplementary Figure S37 Funnel plot with Egger test of rs1800629 in paediatric CD

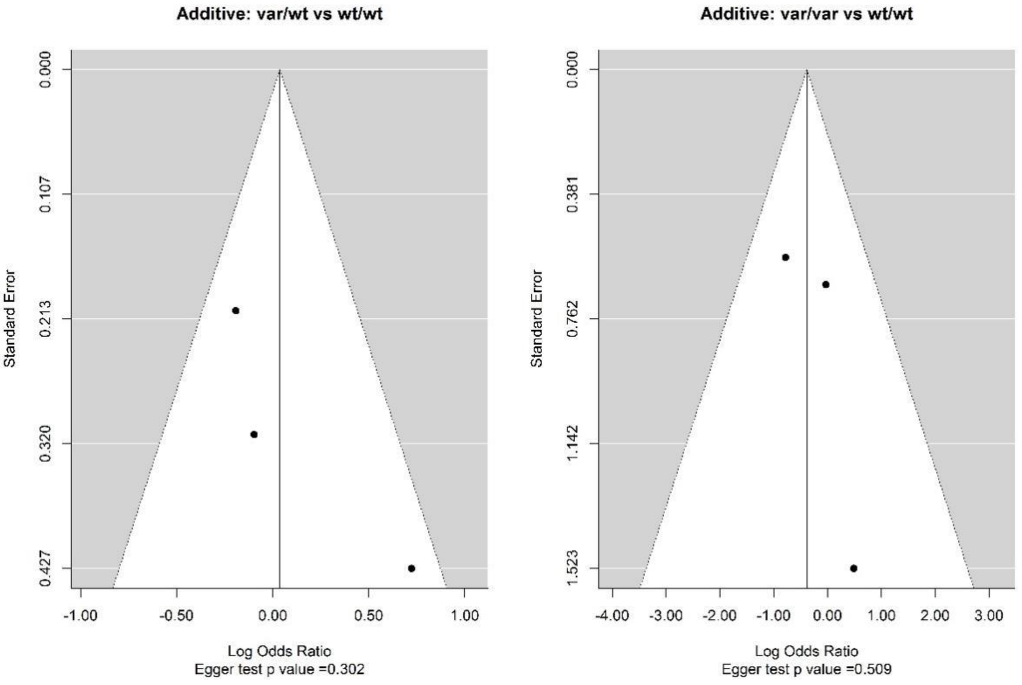

Supplementary Figure S38 Funnel plot with Egger test of rs1799724 in paediatric CD

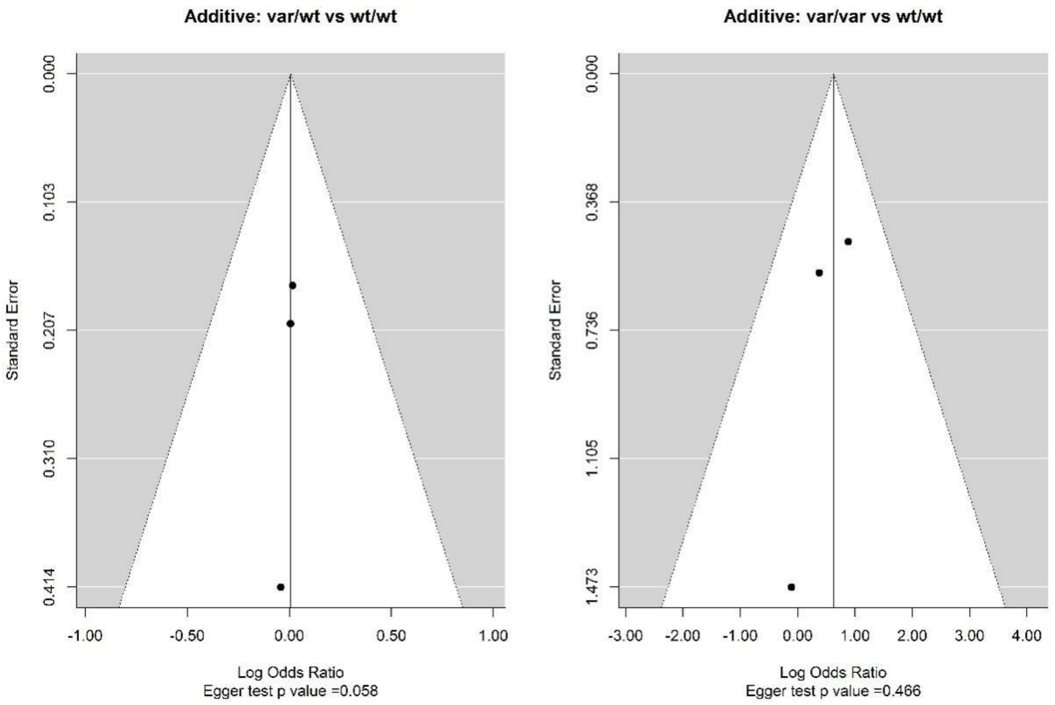

Supplementary Figure S39 Funnel plot with Egger test of rs2542151 in paediatric CD

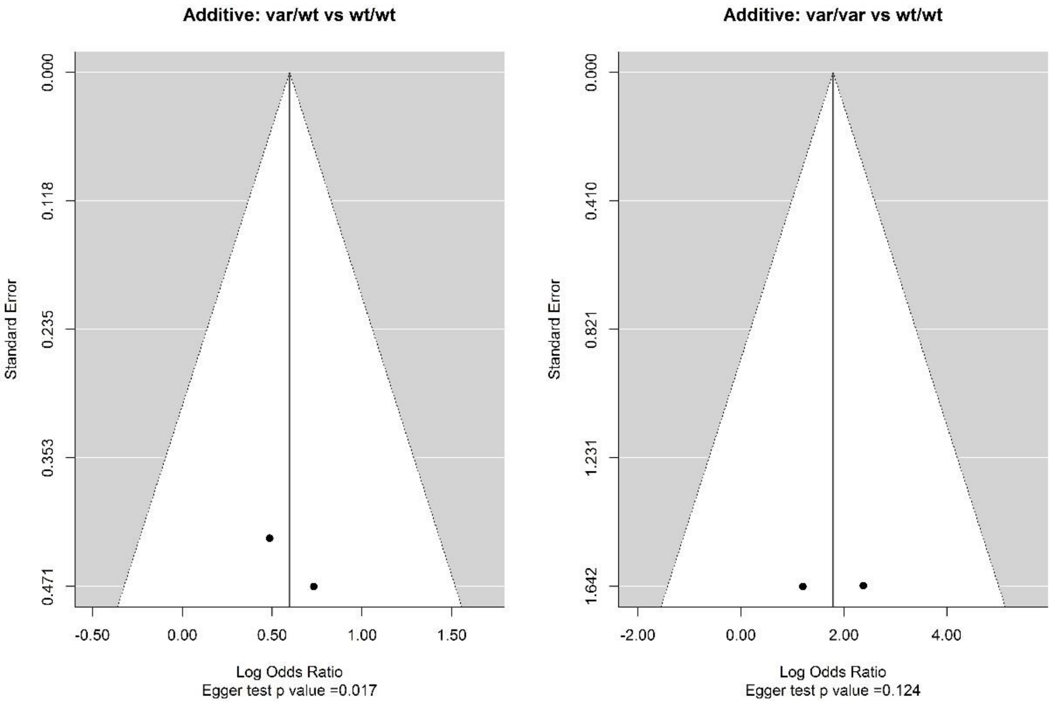

Supplementary Figure S40 Funnel plot with Egger test of rs4986790 in paediatric CD

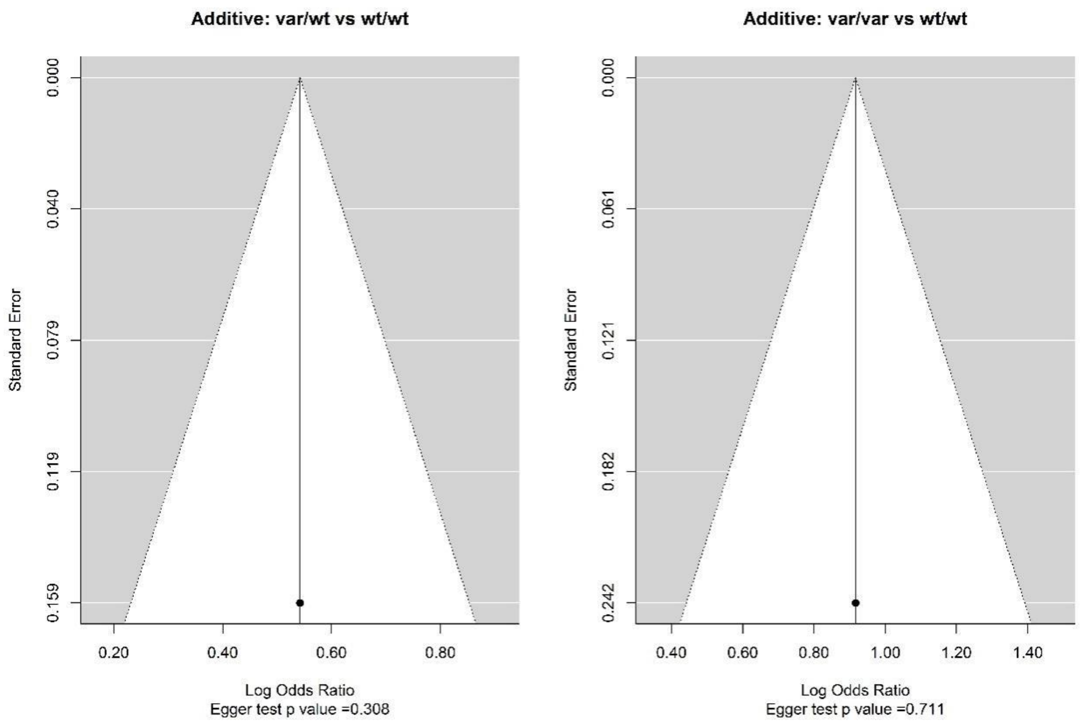

Supplementary Figure S41 Funnel plot with Egger test of rs9858542 in paediatric CD

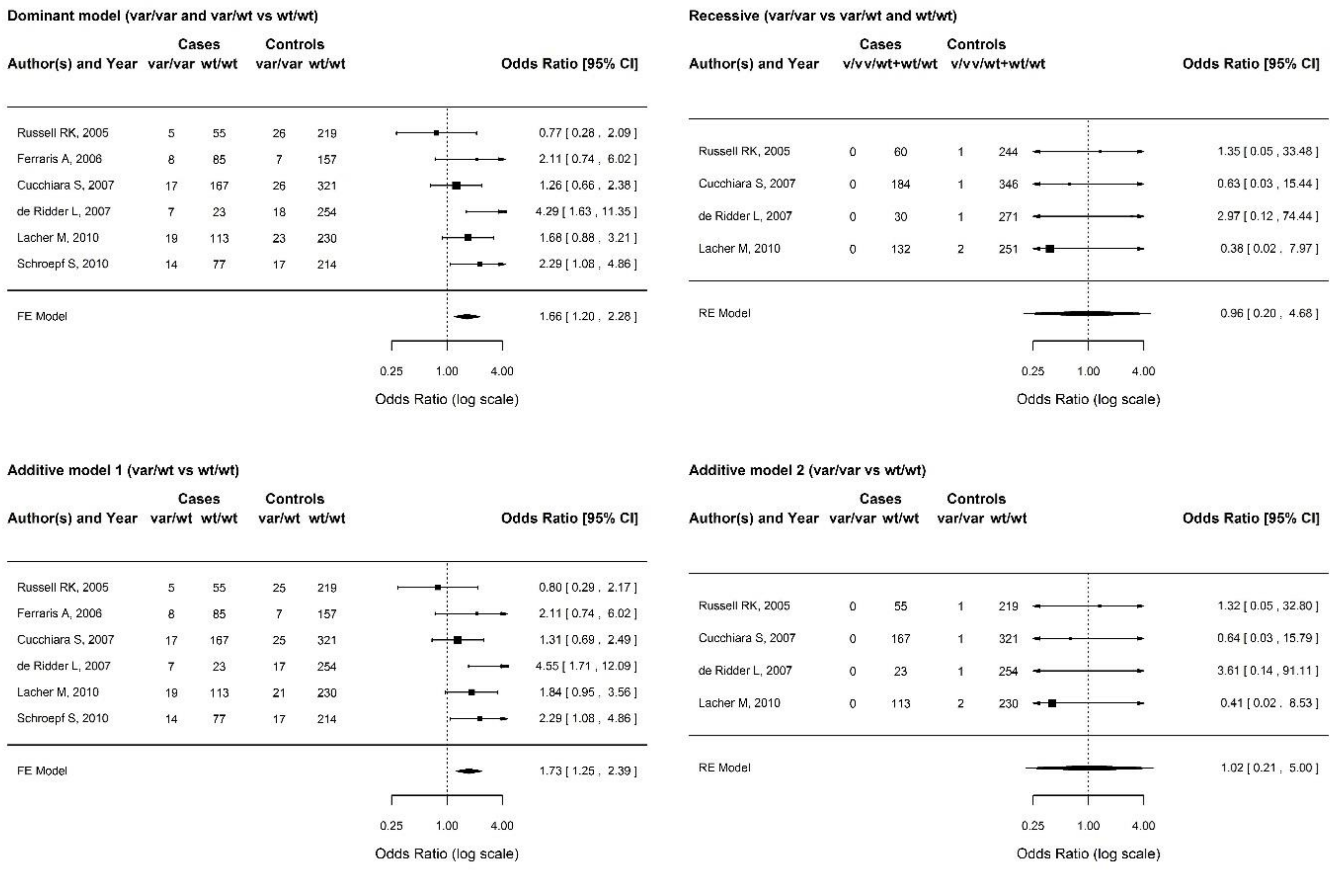

Supplementary Figure S42 Forest plot of rs2066844 in paediatric UC

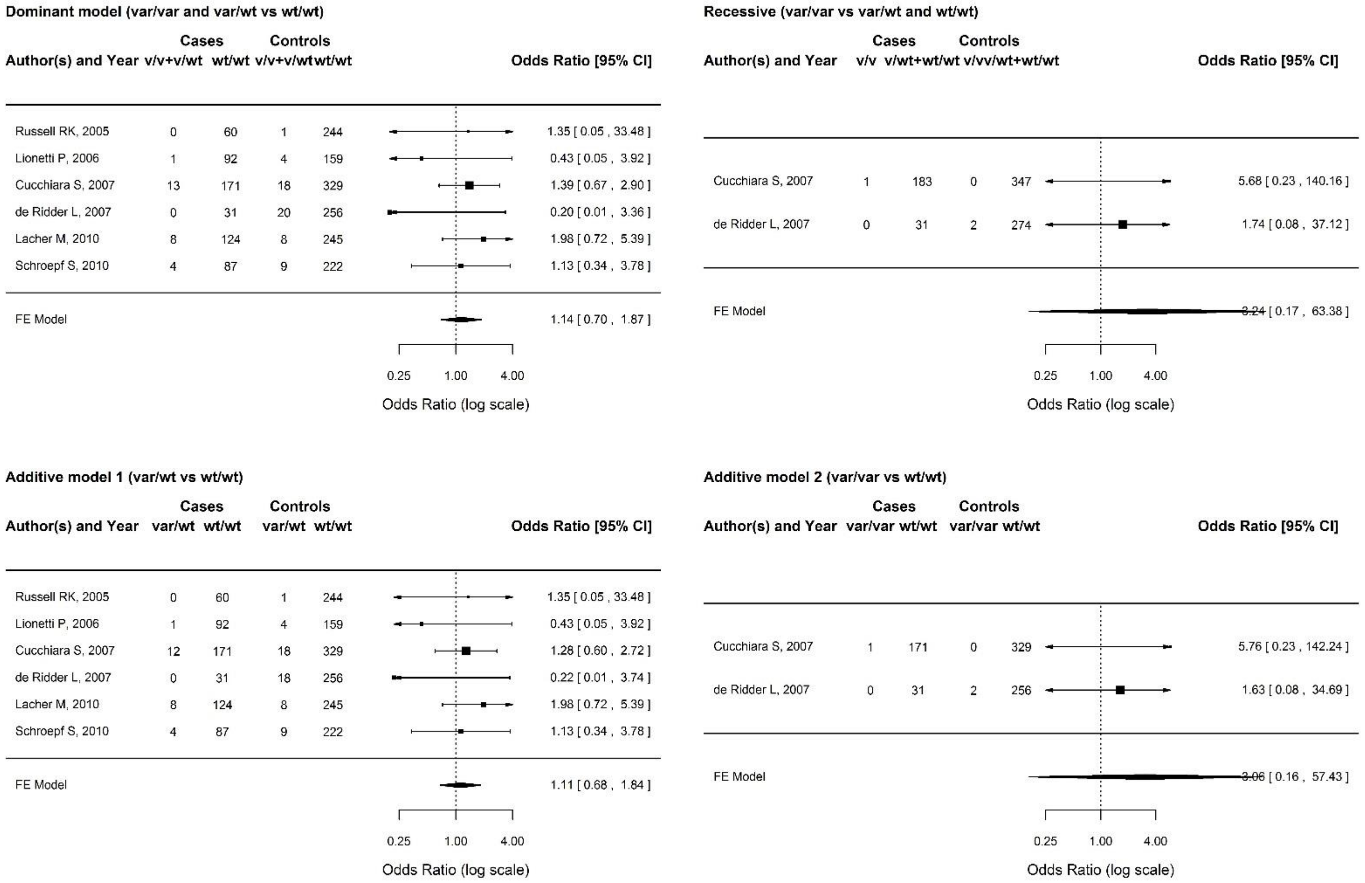

Supplementary Figure S43 Forest plot of rs2066845 in paediatric UC

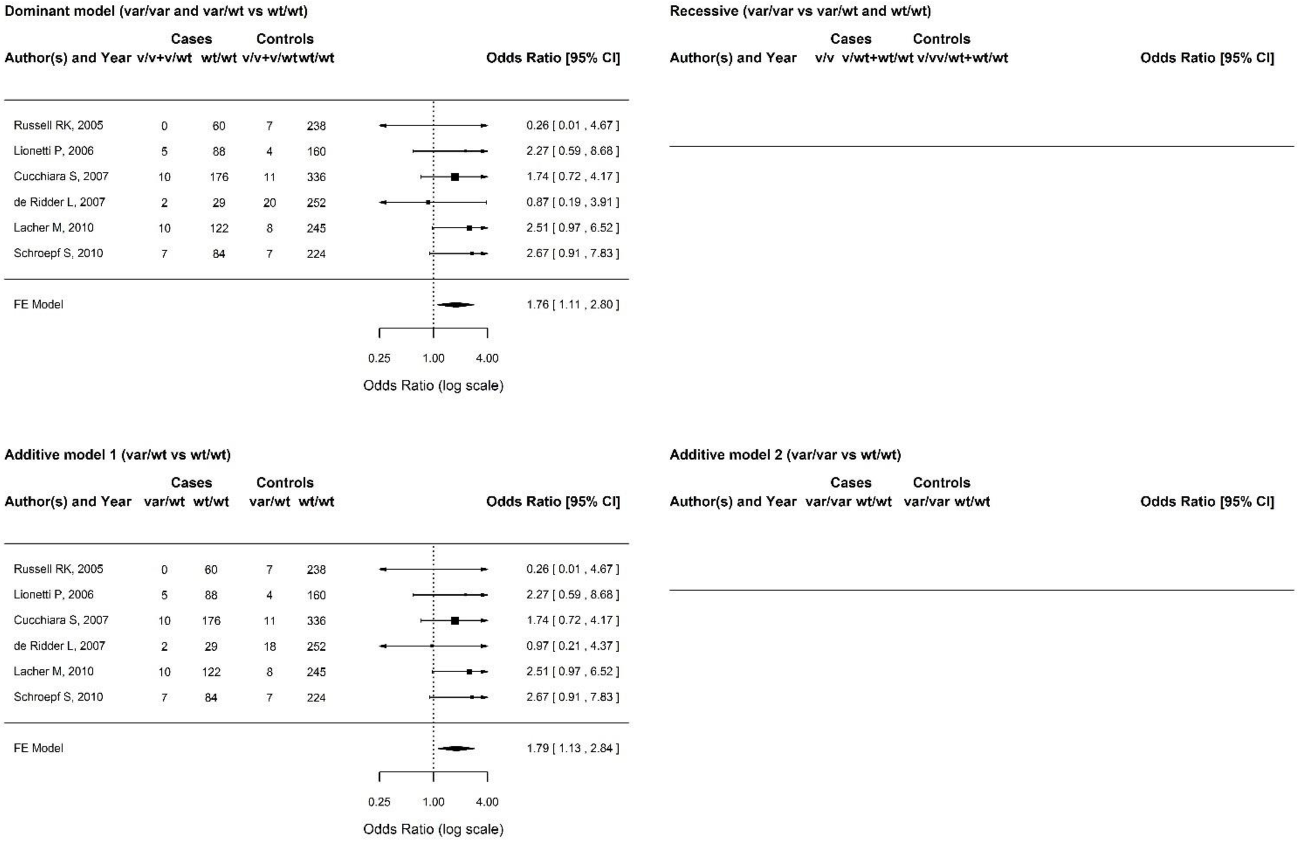

Supplementary Figure S44 Forest plot of rs2066847 in paediatric UC

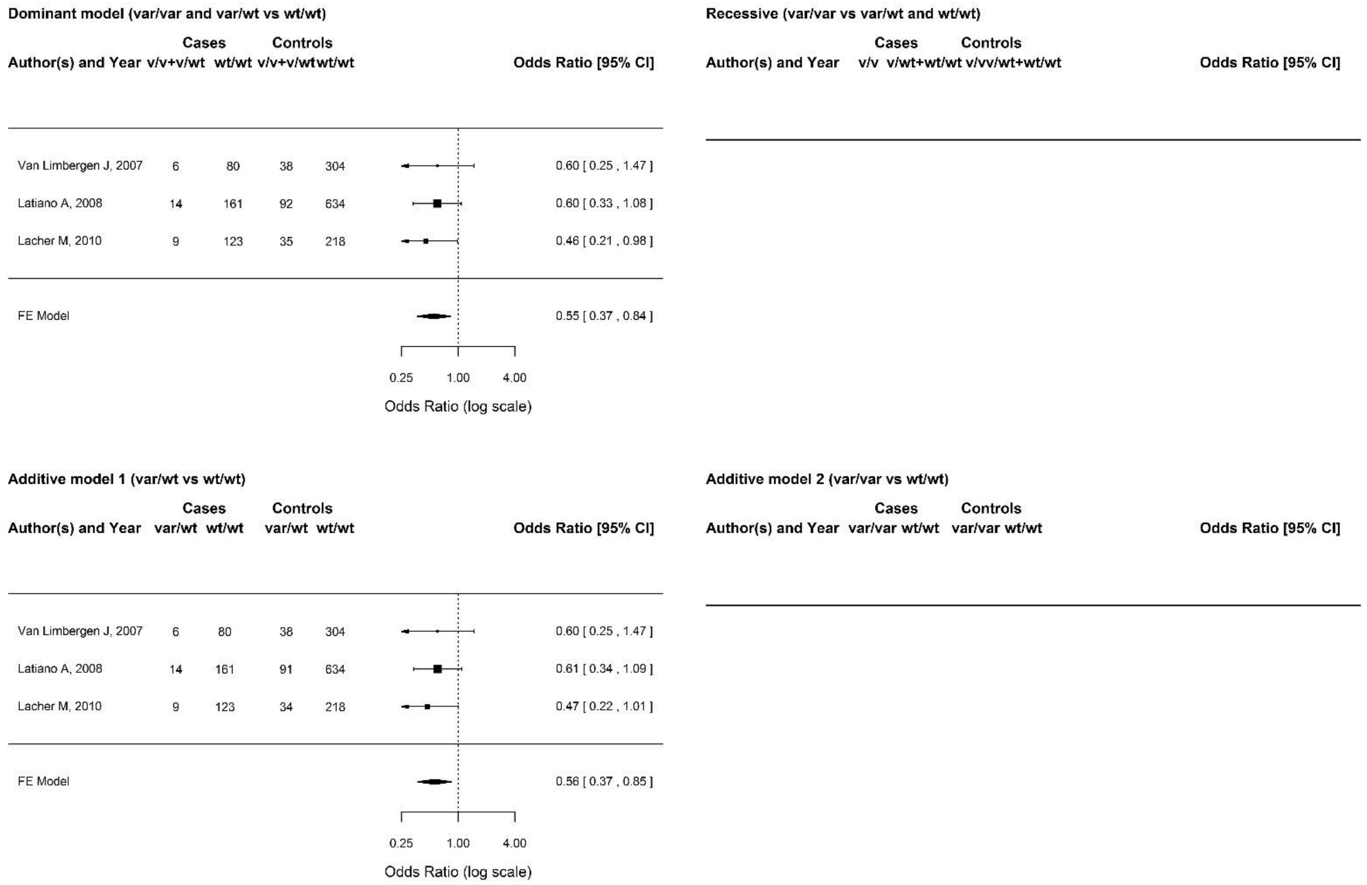

Supplementary Figure S45 Forest plot of rs11209026 in paediatric UC

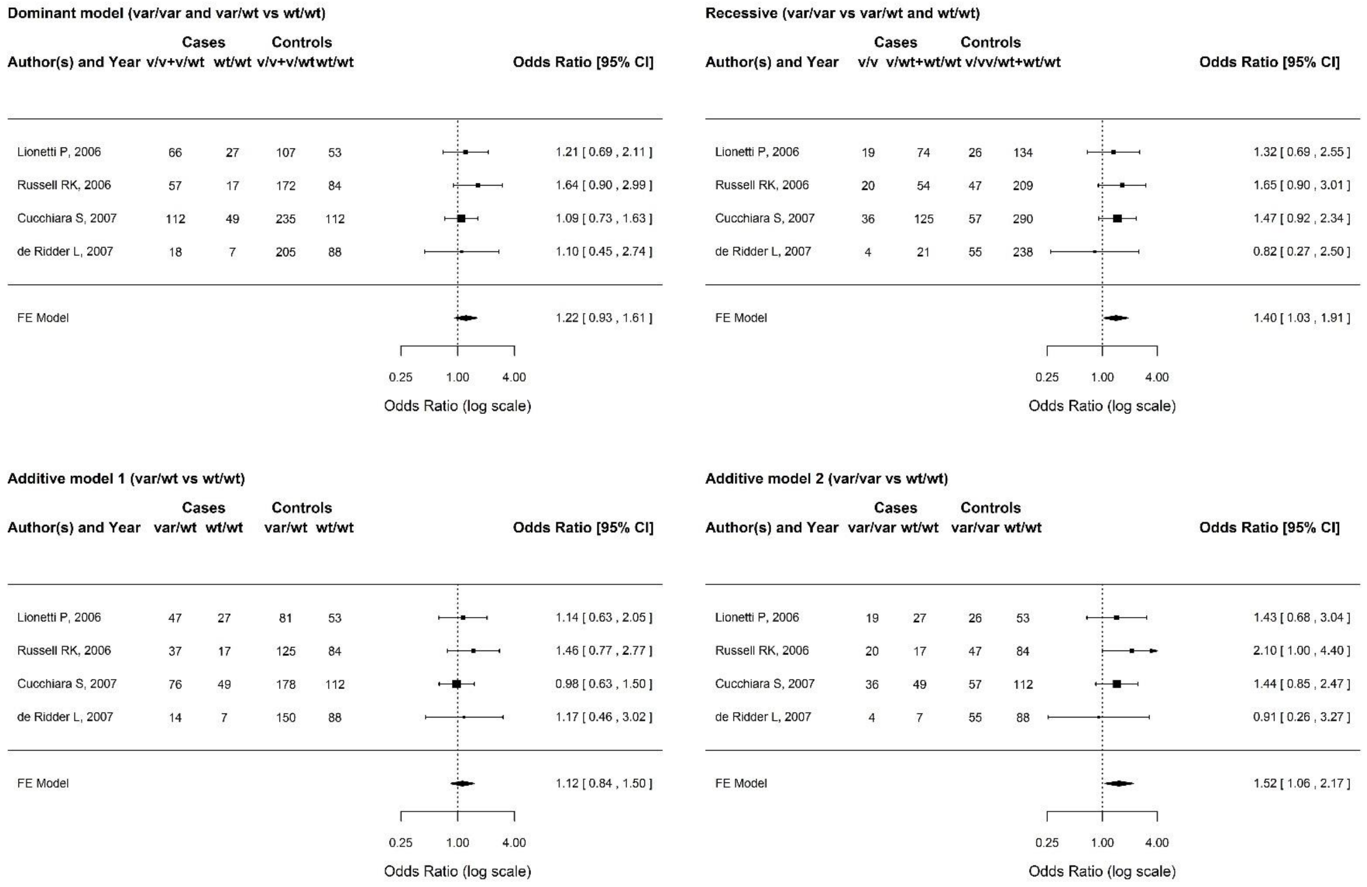

Supplementary Figure S46 Forest plot of rs1050152 in paediatric UC

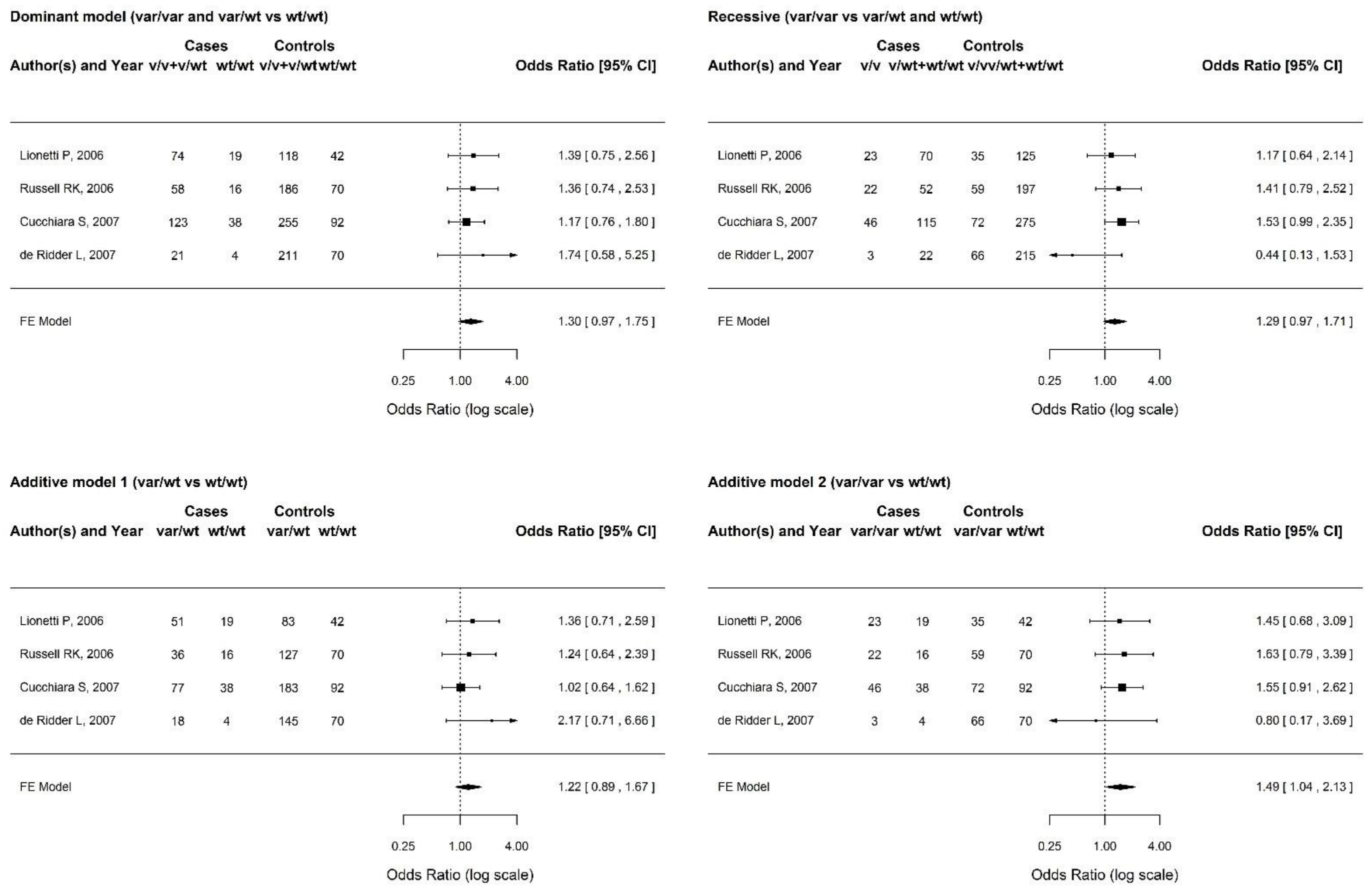

Supplementary Figure S47 Forest plot of rs26313667 in paediatric UC

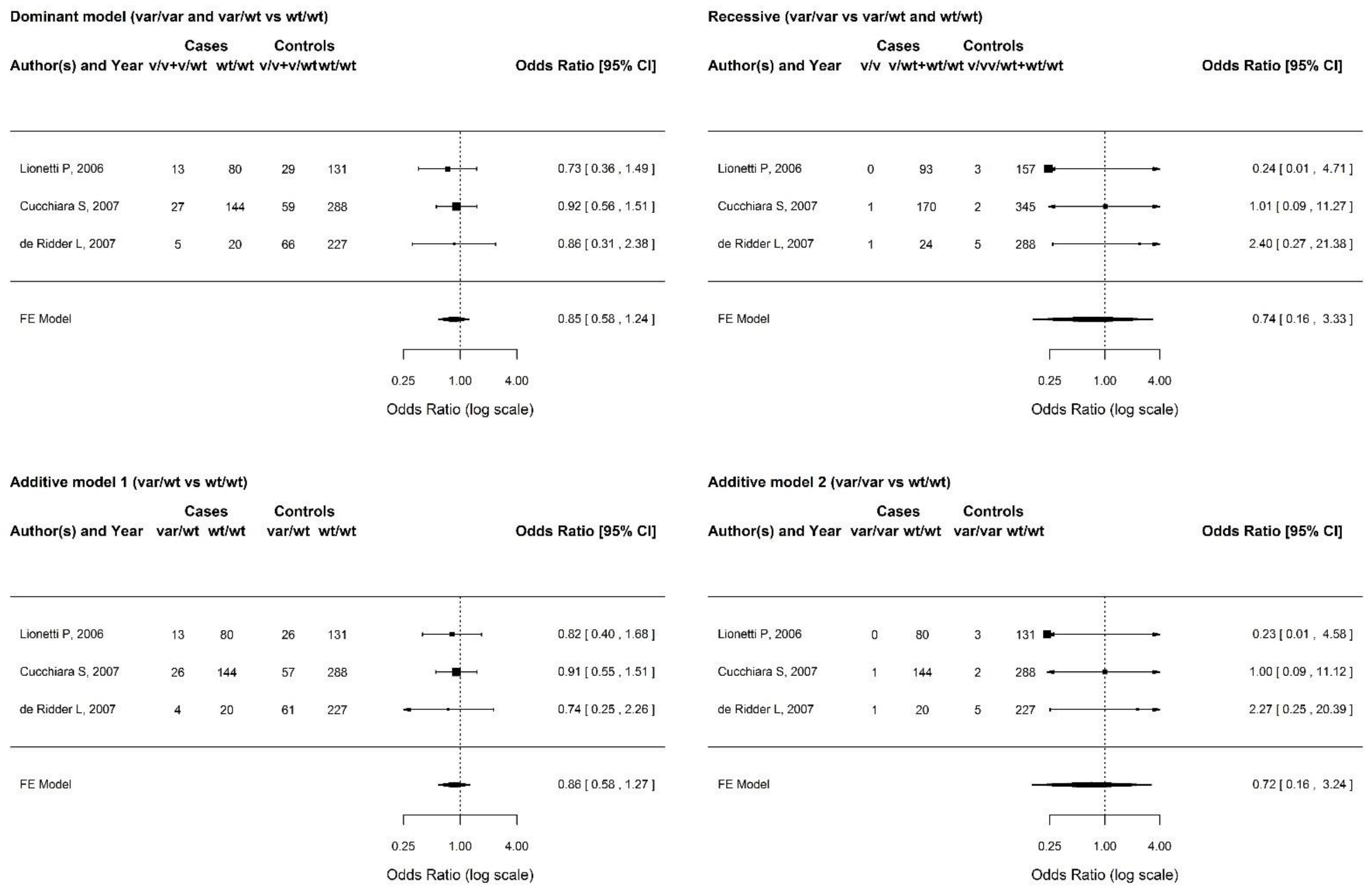

Supplementary Figure S48 Forest plot of rs1248696 in paediatric UC

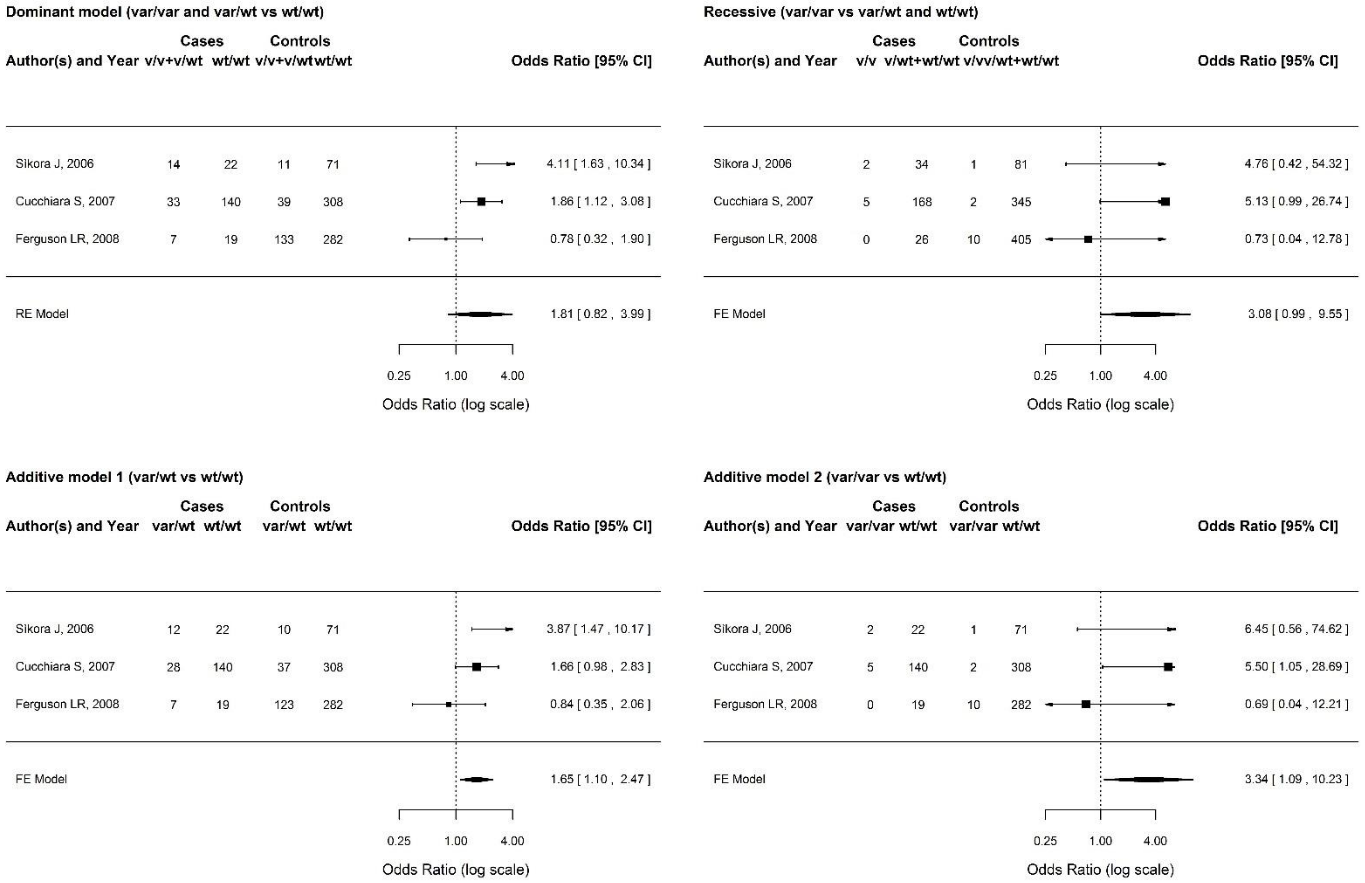

Supplementary Figure S49 Forest plot of rs1800629 in paediatric UC

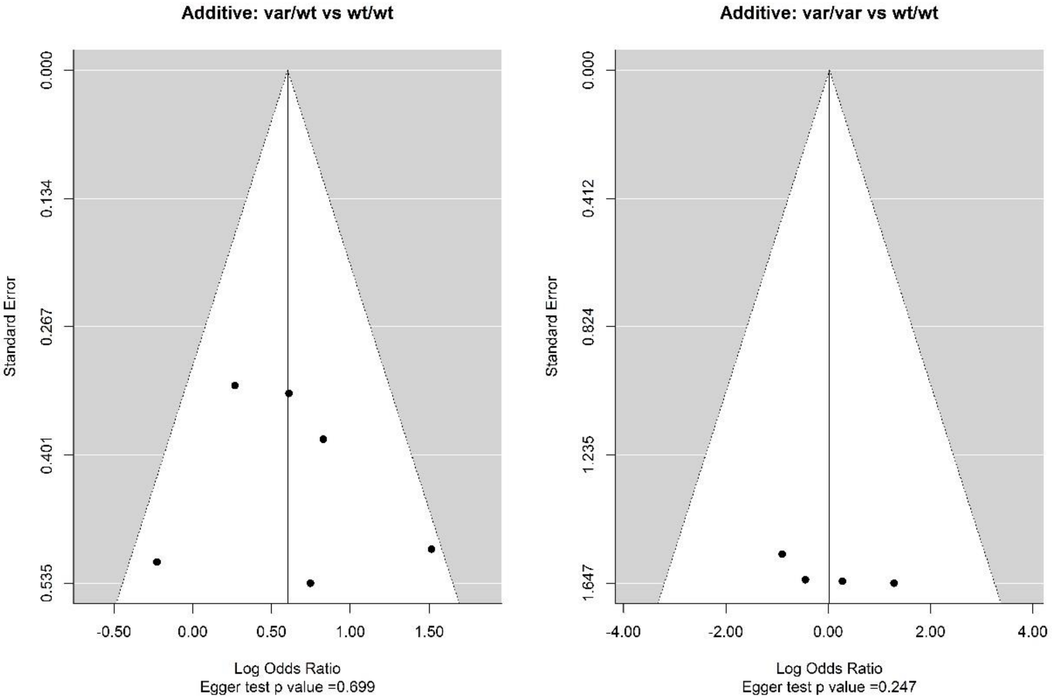

Supplementary Figure S50 Funnel plot with Egger test of rs2066844 in paediatric UC

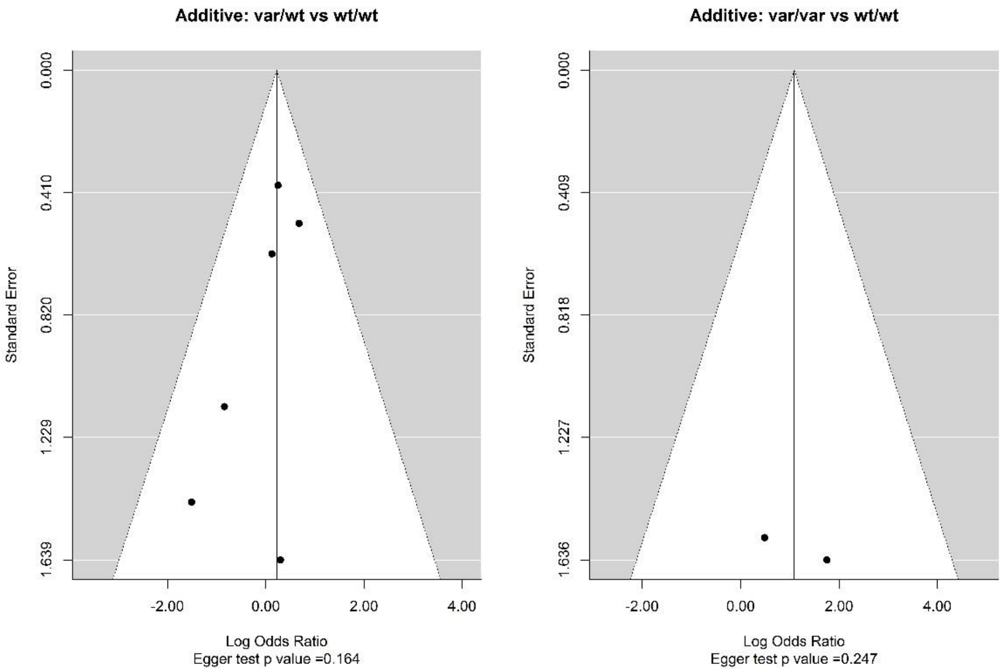

Supplementary Figure S51 Funnel plot with Egger test of rs2066845 in paediatric UC

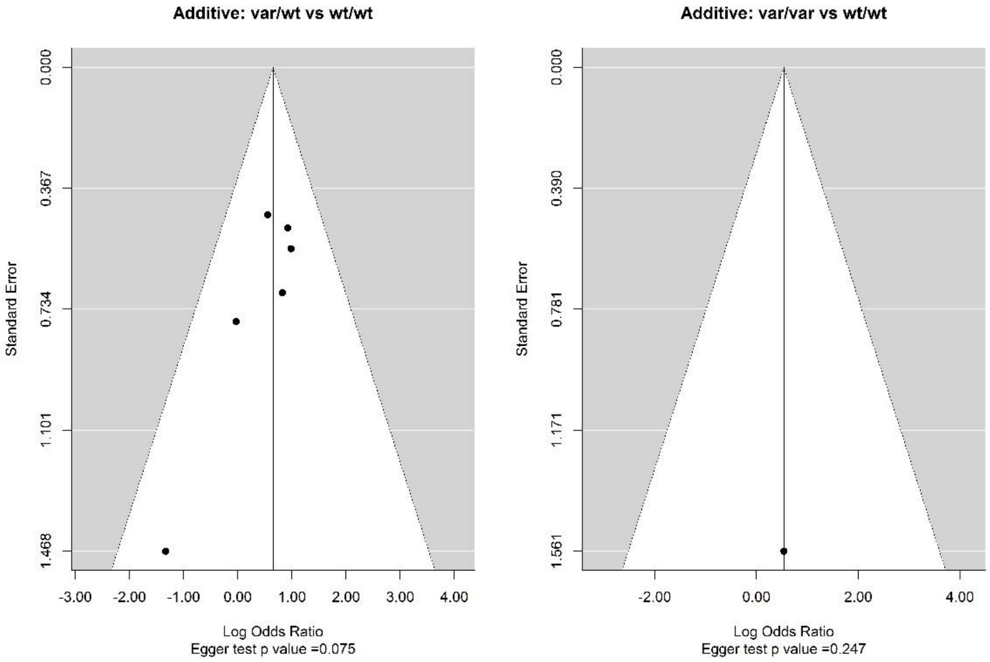

Supplementary Figure S52 Funnel plot with Egger test of rs2066847 in paediatric UC

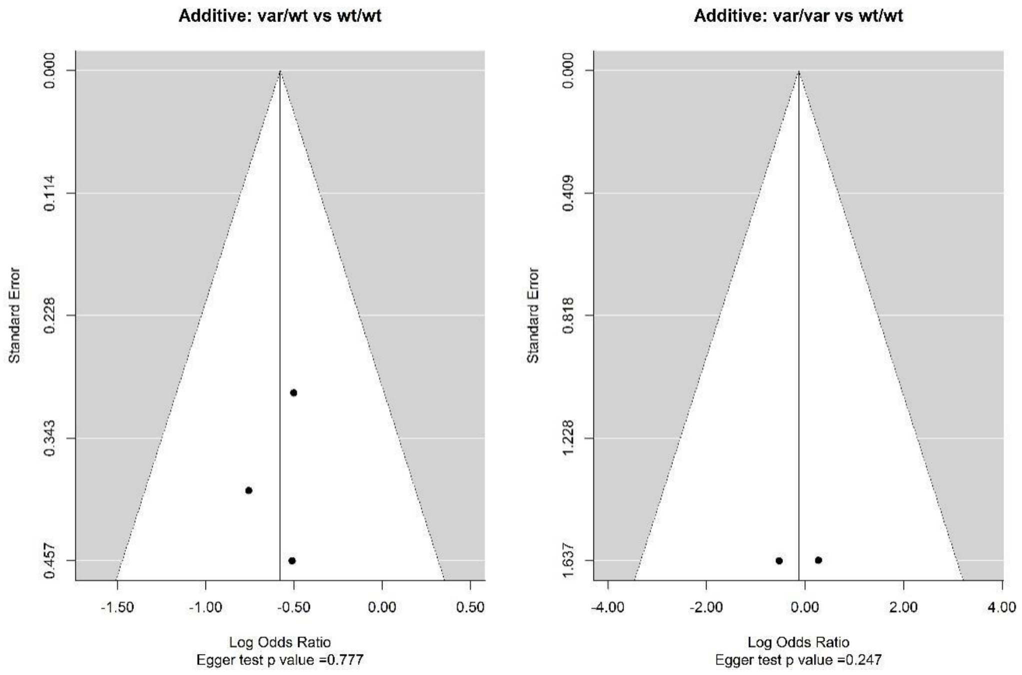

Supplementary Figure S53   Funnel plot with Egger test of rs11209026 in paediatric UC

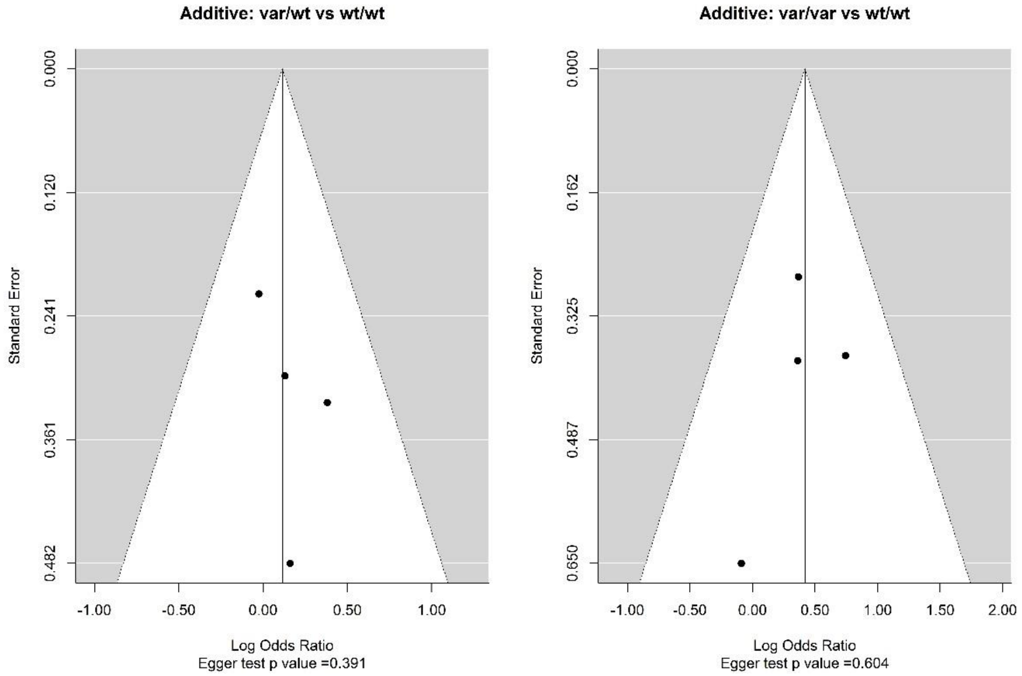

Supplementary Figure S54   Funnel plot with Egger test of rs1050152 in paediatric UC

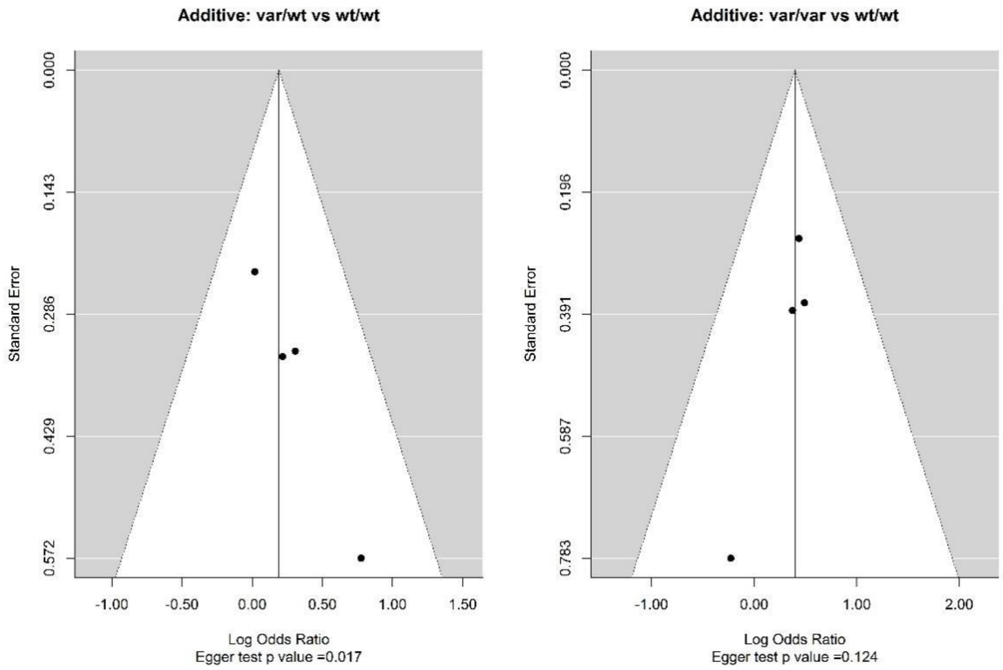

Supplementary Figure S55 Funnel plot with Egger test of rs11209026 in paediatric UC

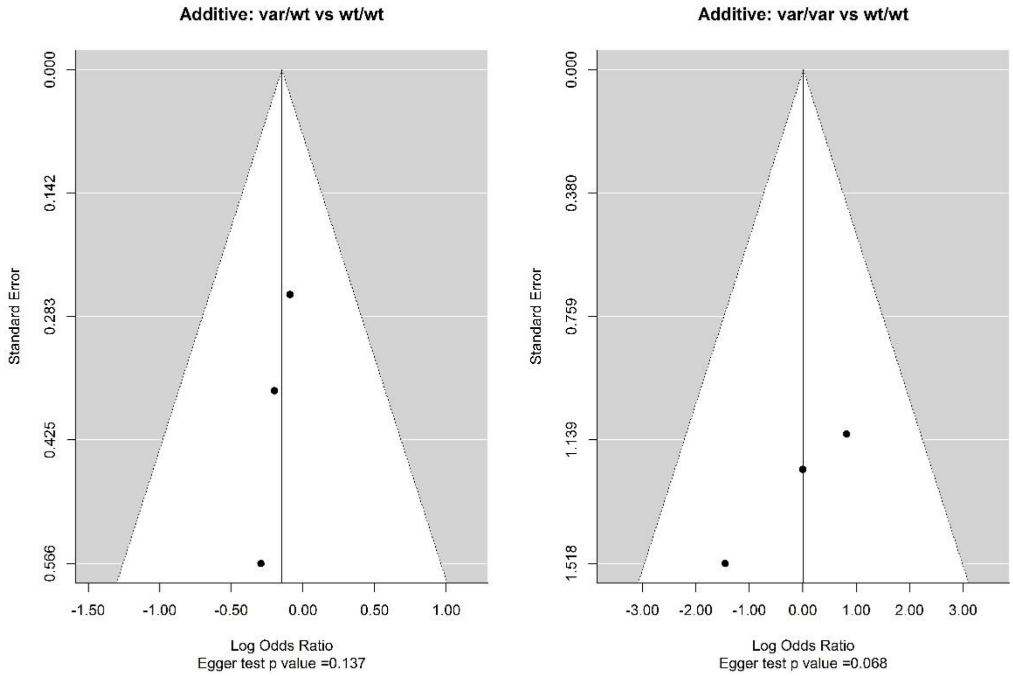

Supplementary Figure S56 Funnel plot with Egger test of rs1248696 in paediatric UC

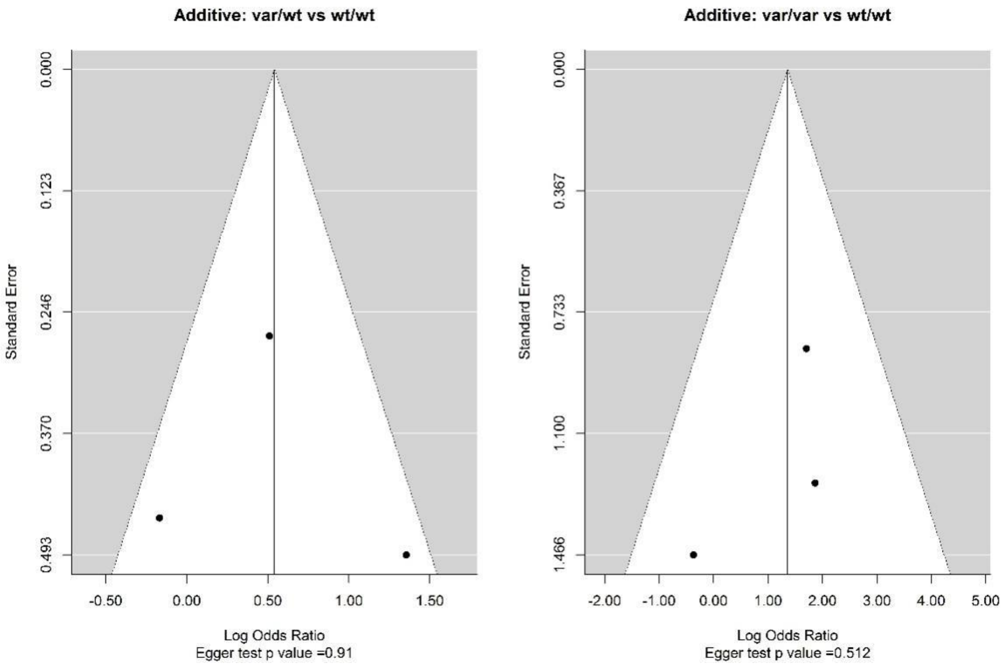

Supplementary Figure S57 Funnel plot with Egger test of rs1800629 in paediatric UC

Supplementary references

1 Sun, L. *et al.* CARD15 genotype and phenotype analysis in 55 pediatric patients with Crohn disease from Saxony, Germany. *Journal of pediatric gastroenterology and nutrition* **37**, 492-497 (2003).

2 Tomer, G., Ceballos, C., Concepcion, E. & Benkov, K. J. NOD2/CARD15 variants are associated with lower weight at diagnosis in children with Crohn's disease. *The American journal of gastroenterology* **98**, 2479-2484, doi:10.1111/j.1572-0241.2003.08673.x (2003).

3 Kugathasan, S. *et al.* Comparative phenotypic and CARD15 mutational analysis among African American, Hispanic, and White children with Crohn's disease. *Inflammatory bowel diseases* **11**, 631-638 (2005).

4 Levine, A. *et al.* A polymorphism in the TNF-alpha promoter gene is associated with pediatric onset and colonic location of Crohn's disease. *The American journal of gastroenterology* **100**, 407-413, doi:10.1111/j.1572-0241.2005.41126.x (2005).

5 Russell, R. K. *et al.* Genotype-phenotype analysis in childhood-onset Crohn's disease: NOD2/CARD15 variants consistently predict phenotypic characteristics of severe disease. *Inflammatory bowel diseases* **11**, 955-964 (2005).

6 Ferraris, A. *et al.* Relationship between CARD15, SLC22A4/5, and DLG5 polymorphisms and early-onset inflammatory bowel diseases: an Italian multicentric study. *Inflammatory bowel diseases* **12**, 355-361, doi:10.1097/01.mib.0000217338.23065.58 (2006).

7 Russell, R. K. *et al.* Analysis of the influence of OCTN1/2 variants within the IBD5 locus on disease susceptibility and growth indices in early onset inflammatory bowel disease. *Gut* **55**, 1114-1123, doi:10.1136/gut.2005.082107 (2006).

8 Sykora, J. *et al.* Cytokine tumor necrosis factor-alpha A promoter gene polymorphism at position -308 G-->A and pediatric inflammatory bowel disease: implications in ulcerative colitis and Crohn's disease. *Journal of pediatric gastroenterology and nutrition* **42**, 479-487, doi:10.1097/01.mpg.0000221917.80887.9e (2006).

9 Baldassano, R. N. *et al.* Association of the T300A non-synonymous variant of the ATG16L1 gene with susceptibility to paediatric Crohn's disease. *Gut* **56**, 1171-1173, doi:10.1136/gut.2007.122747 (2007).

10 Browning, B. L. *et al.* Association of DLG5 variants with inflammatory bowel disease in the New Zealand Caucasian population and meta-analysis of the DLG5 R30Q variant. *Inflammatory bowel diseases* **13**, 1069-1076 (2007).

11 Cucchiara, S. *et al.* Polymorphisms of tumor necrosis factor-alpha but not MDR1 influence response to medical therapy in pediatric-onset inflammatory bowel disease. *Journal of pediatric gastroenterology and nutrition* **44**, 171-179, doi:10.1097/MPG.0b013e31802c41f3 (2007).

12 Cucchiara, S. *et al.* Role of CARD15, DLG5 and OCTN genes polymorphisms in children with inflammatory bowel diseases. *World journal of gastroenterology* **13**, 1221-1229 (2007).

13 Cummings, J. R. *et al.* Confirmation of the role of ATG16L1 as a Crohn's disease susceptibility gene. *Inflammatory bowel diseases* **13**, 941-946, doi:10.1002/ibd.20162 (2007).

14 De Iudicibus, S. *et al.* Association of BclI polymorphism of the glucocorticoid receptor gene locus with response to glucocorticoids in inflammatory bowel disease. *Gut* **56**, 1319-1320, doi:10.1136/gut.2006.116160 (2007).

15 de Ridder, L. *et al.* Genetic susceptibility has a more important role in pediatric-onset Crohn's disease than in adult-onset Crohn's disease. *Inflammatory bowel diseases* **13**, 1083-1092, doi:10.1002/ibd.20171 (2007).

16 Gearry, R. B. *et al.* Effect of inflammatory bowel disease classification changes on NOD2 genotype-phenotype associations in a population-based cohort. *Inflammatory bowel diseases* **13**, 1220-1227, doi:10.1002/ibd.20190 (2007).

17 Glas, J. *et al.* rs1004819 is the main disease-associated IL23R variant in German Crohn's disease patients: combined analysis of IL23R, CARD15, and OCTN1/2 variants. *PloS one* **2**, e819, doi:10.1371/journal.pone.0000819 (2007).

18 Leshinsky-Silver, E. *et al.* Evaluation of the interleukin-23 receptor gene coding variant R381Q in pediatric and adult Crohn disease. *Journal of pediatric gastroenterology and nutrition* **45**, 405-408, doi:10.1097/MPG.0b013e318141a1de (2007).

19 Lacher, M. *et al.* Association of a CXCL9 polymorphism with pediatric Crohn's disease. *Biochemical and biophysical research communications* **363**, 701-707, doi:10.1016/j.bbrc.2007.09.020 (2007).

20 Nam, S. Y. *et al.* Heat shock protein gene 70-2 polymorphism is differentially associated with the clinical phenotypes of ulcerative colitis and Crohn's disease. *Journal of gastroenterology and hepatology* **22**, 1032-1038, doi:10.1111/j.1440-1746.2007.04927.x (2007).

21 Roberts, R. L. *et al.* IL23R R381Q and ATG16L1 T300A are strongly associated with Crohn's disease in a study of New Zealand Caucasians with inflammatory bowel disease. *The American journal of gastroenterology* **102**, 2754-2761, doi:10.1111/j.1572-0241.2007.01525.x (2007).

22 Van Limbergen, J. *et al.* Investigation of NOD1/CARD4 variation in inflammatory bowel disease using a haplotype-tagging strategy. *Human molecular genetics* **16**, 2175-2186, doi:10.1093/hmg/ddm169 (2007).
23 Van Limbergen, J. *et al.* IL23R Arg381Gln is associated with childhood onset inflammatory bowel disease in Scotland. *Gut* **56**, 1173-1174, doi:10.1136/gut.2007.122069 (2007).
24 Van Limbergen, J. *et al.* Contribution of the NOD1/CARD4 insertion/deletion polymorphism +32656 to inflammatory bowel disease in Northern Europe. *Inflammatory bowel diseases* **13**, 882-889, doi:10.1002/ibd.20124 (2007).
25 Ferguson, L. R. *et al.* Single nucleotide polymorphisms in human Paneth cell defensin A5 may confer susceptibility to inflammatory bowel disease in a New Zealand Caucasian population. *Digestive and liver disease : official journal of the Italian Society of Gastroenterology and the Italian Association for the Study of the Liver* **40**, 723-730, doi:10.1016/j.dld.2008.02.011 (2008).
26 Ferguson, L. R. *et al.* Single nucleotide polymorphism in the tumor necrosis factor-alpha gene affects inflammatory bowel diseases risk. *World journal of gastroenterology* **14**, 4652-4661 (2008).
27 Glas, J. *et al.* The ATG16L1 gene variants rs2241879 and rs2241880 (T300A) are strongly associated with susceptibility to Crohn's disease in the German population. *The American journal of gastroenterology* **103**, 682-691, doi:10.1111/j.1572-0241.2007.01694.x (2008).
28 Hradsky, O. *et al.* Variants of CARD15, TNFA and PTPN22 and susceptibility to Crohn's disease in the Czech population: high frequency of the CARD15 1007fs. *Tissue antigens* **71**, 538-547, doi:10.1111/j.1399-0039.2008.01047.x (2008).
29 Latiano, A. *et al.* Replication of interleukin 23 receptor and autophagy-related 16-like 1 association in adult- and pediatric-onset inflammatory bowel disease in Italy. *World journal of gastroenterology* **14**, 4643-4651 (2008).
30 Perricone, C. *et al.* ATG16L1 Ala197Thr is not associated with susceptibility to Crohn's disease or with phenotype in an Italian population. *Gastroenterology* **134**, 368-370, doi:10.1053/j.gastro.2007.11.017 (2008).
31 Seiderer, J. *et al.* Role of the novel Th17 cytokine IL-17F in inflammatory bowel disease (IBD): upregulated colonic IL-17F expression in active Crohn's disease and analysis of the IL17F p.His161Arg polymorphism in IBD. *Inflammatory bowel diseases* **14**, 437-445, doi:10.1002/ibd.20339 (2008).
32 Van Limbergen, J. *et al.* Autophagy gene ATG16L1 influences susceptibility and disease location but not childhood-onset in Crohn's disease in Northern Europe. *Inflammatory bowel diseases* **14**, 338-346, doi:10.1002/ibd.20340 (2008).
33 Chen, B. *et al.* Association of interleukin-17F 7488 single nucleotide polymorphism and inflammatory bowel disease in the Chinese population. *Scandinavian journal of gastroenterology* **44**, 720-726, doi:10.1080/00365520902795430 (2009).
34 Bueno de Mesquita, M. *et al.* Clustering of (auto)immune diseases with early-onset and complicated inflammatory bowel disease. *European journal of pediatrics* **168**, 575-583, doi:10.1007/s00431-008-0798-7 (2009).
35 Ferguson, L. R. *et al.* Tumor necrosis factor receptor superfamily, member 1B haplotypes increase or decrease the risk of inflammatory bowel diseases in a New Zealand caucasian population. *Gastroenterology research and practice* **2009**, 591704, doi:10.1155/2009/591704 (2009).
36 Huebner, C. *et al.* Nucleotide-binding oligomerization domain containing 1 (NOD1) haplotypes and single nucleotide polymorphisms modify susceptibility to inflammatory bowel diseases in a New Zealand caucasian population: a case-control study. *BMC research notes* **2**, 52, doi:10.1186/1756-0500-2-52 (2009).
37 Koslowski, M. J. *et al.* Genetic variants of Wnt transcription factor TCF-4 (TCF7L2) putative promoter region are associated with small intestinal Crohn's disease. *PloS one* **4**, e4496, doi:10.1371/journal.pone.0004496 (2009).
38 Lacher, M. *et al.* Nuclear pregnane X receptor single nucleotide polymorphism (-25385C/T) is not associated with inflammatory bowel disease in pediatric patients. *Journal of pediatric gastroenterology and nutrition* **49**, 147-150, doi:10.1097/MPG.0b013e31818de1a8 (2009).
39 Lacher, M. *et al.* Autophagy 16-like 1 rs2241880 G allele is associated with Crohn's disease in German children. *Acta paediatrica (Oslo, Norway : 1992)* **98**, 1835-1840, doi:10.1111/j.1651-2227.2009.01438.x (2009).
40 Latiano, A. *et al.* Polymorphism of the IRGM gene might predispose to fistulizing behavior in Crohn's disease. *The American journal of gastroenterology* **104**, 110-116, doi:10.1038/ajg.2008.3 (2009).
41 Tomer, G., Wetzler, G., Keddache, M. & Denson, L. A. Polymorphisms in the IBD5 locus are associated with Crohn disease in pediatric Ashkenazi Jewish patients. *Journal of pediatric gastroenterology and nutrition* **48**, 531-537, doi:10.1097/MPG.0b013e318183138a (2009).
42 Torok, H. P. *et al.* Epistasis between Toll-like receptor-9 polymorphisms and variants in NOD2 and IL23R modulates susceptibility to Crohn's disease. *The American journal of gastroenterology* **104**, 1723-1733, doi:10.1038/ajg.2009.184 (2009).

43 Van Limbergen, J. *et al.* Germline variants of IRGM in childhood-onset Crohn's disease. *Gut* **58**, 610-611, doi:10.1136/gut.2008.160721 (2009).
44 Van Limbergen, J. *et al.* Filaggrin loss-of-function variants are associated with atopic comorbidity in pediatric inflammatory bowel disease. *Inflammatory bowel diseases* **15**, 1492-1498, doi:10.1002/ibd.20926 (2009).
45 Amre, D. K. *et al.* Susceptibility loci reported in genome-wide association studies are associated with Crohn's disease in Canadian children. *Alimentary pharmacology & therapeutics* **31**, 1186-1191, doi:10.1111/j.1365-2036.2010.04294.x (2010).
46 Aoyagi, Y. *et al.* Peroxisome proliferator-activated receptor gamma 2 mutation may cause a subset of ulcerative colitis. *Pediatrics international : official journal of the Japan Pediatric Society* **52**, 729-734, doi:10.1111/j.1442-200X.2010.03195.x (2010).
47 Ferguson, L. R. *et al.* Genetic factors in chronic inflammation: single nucleotide polymorphisms in the STAT-JAK pathway, susceptibility to DNA damage and Crohn's disease in a New Zealand population. *Mutation research* **690**, 108-115, doi:10.1016/j.mrfmmm.2010.01.017 (2010).
48 Ferguson, L. R. *et al.* IL23R and IL12B SNPs and Haplotypes Strongly Associate with Crohn's Disease Risk in a New Zealand Population. *Gastroenterology research and practice* **2010**, 539461, doi:10.1155/2010/539461 (2010).
49 Gazouli, M. *et al.* NOD2/CARD15, ATG16L1 and IL23R gene polymorphisms and childhood-onset of Crohn's disease. *World journal of gastroenterology* **16**, 1753-1758 (2010).
50 Glas, J. *et al.* The NOD2 single nucleotide polymorphisms rs2066843 and rs2076756 are novel and common Crohn's disease susceptibility gene variants. *PloS one* **5**, e14466, doi:10.1371/journal.pone.0014466 (2010).
51 Glas, J. *et al.* Evidence for STAT4 as a common autoimmune gene: rs7574865 is associated with colonic Crohn's disease and early disease onset. *PloS one* **5**, e10373, doi:10.1371/journal.pone.0010373 (2010).
52 Lacher, M. *et al.* Hirschsprung-associated enterocolitis develops independently of NOD2 variants. *Journal of pediatric surgery* **45**, 1826-1831, doi:10.1016/j.jpedsurg.2010.02.039 (2010).
53 Lacher, M. *et al.* Association of the interleukin-23 receptor gene variant rs11209026 with Crohn's disease in German children. *Acta paediatrica (Oslo, Norway : 1992)* **99**, 727-733, doi:10.1111/j.1651-2227.2009.01680.x (2010).
54 Lacher, M. *et al.* NOD2 mutations predict the risk for surgery in pediatric-onset Crohn's disease. *Journal of pediatric surgery* **45**, 1591-1597, doi:10.1016/j.jpedsurg.2009.10.046 (2010).
55 Latiano, A. *et al.* Variants at the 3p21 locus influence susceptibility and phenotype both in adults and early-onset patients with inflammatory bowel disease. *Inflammatory bowel diseases* **16**, 1108-1117, doi:10.1002/ibd.21176 (2010).
56 Morgan, A. R. *et al.* PTPN2 but not PTPN22 is associated with Crohn's disease in a New Zealand population. *Tissue antigens* **76**, 119-125, doi:10.1111/j.1399-0039.2010.01493.x (2010).
57 Roberts, R. L. *et al.* Evidence of interaction of CARD8 rs2043211 with NALP3 rs35829419 in Crohn's disease. *Genes and immunity* **11**, 351-356, doi:10.1038/gene.2010.11 (2010).
58 Schroepf, S. *et al.* Strong overexpression of CXCR3 axis components in childhood inflammatory bowel disease. *Inflammatory bowel diseases* **16**, 1882-1890, doi:10.1002/ibd.21312 (2010).
59 Wagner, J. *et al.* Interaction of Crohn's disease susceptibility genes in an Australian paediatric cohort. *PloS one* **5**, e15376, doi:10.1371/journal.pone.0015376 (2010).
60 Bak-Romaniszyn, L. *et al.* Mannan-binding lectin deficiency in pediatric patients with inflammatory bowel disease. *Scandinavian journal of gastroenterology* **46**, 1275-1278, doi:10.3109/00365521.2011.594087 (2011).
61 Diaz-Gallo, L. M. *et al.* Analysis of the influence of two CD24 genetic variants in Crohn's disease and ulcerative colitis. *Human immunology* **72**, 969-972, doi:10.1016/j.humimm.2011.05.028 (2011).
62 Latiano, A. *et al.* Investigation of multiple susceptibility loci for inflammatory bowel disease in an Italian cohort of patients. *PloS one* **6**, e22688, doi:10.1371/journal.pone.0022688 (2011).
63 Repnik, K. & Potocnik, U. Haplotype in the IBD5 region is associated with refractory Crohn's disease in Slovenian patients and modulates expression of the SLC22A5 gene. *Journal of gastroenterology* **46**, 1081-1091, doi:10.1007/s00535-011-0426-6 (2011).
64 Wang, A. H. *et al.* The effect of IL-10 genetic variation and interleukin 10 serum levels on Crohn's disease susceptibility in a New Zealand population. *Human immunology* **72**, 431-435, doi:10.1016/j.humimm.2011.02.014 (2011).
65 Wolters, V. M. *et al.* Replication of genetic variation in the MYO9B gene in Crohn's disease. *Human immunology* **72**, 592-597, doi:10.1016/j.humimm.2011.03.025 (2011).
66 de Vries, H. S., Te Morsche, R. H., Jenniskens, K., Peters, W. H. & de Jong, D. J. A functional polymorphism in UGT1A1 related to hyperbilirubinemia is associated with a decreased risk for Crohn's disease. *Journal of Crohn's & colitis* **6**, 597-602, doi:10.1016/j.crohns.2011.11.010 (2012).

67 Glas, J. *et al.* PTPN2 gene variants are associated with susceptibility to both Crohn's disease and ulcerative colitis supporting a common genetic disease background. *PloS one* **7**, e33682, doi:10.1371/journal.pone.0033682 (2012).
68 Glas, J. *et al.* PTGER4 Expression-Modulating Polymorphisms in the 5p13.1 Region Predispose to Crohn's Disease and Affect NF-B and XBP1 Binding Sites. *PloS one* **7** (2012).
69 Mazzoccoli, G. *et al.* Association study of a polymorphism in clock gene PERIOD3 and risk of inflammatory bowel disease. *Chronobiology international* **29**, 994-1003, doi:10.3109/07420528.2012.705935 (2012).
70 Morgan, A. R., Lam, W. J., Han, D. Y., Fraser, A. G. & Ferguson, L. R. Genetic variation within TLR10 is associated with Crohn's disease in a New Zealand population. *Human immunology* **73**, 416-420, doi:10.1016/j.humimm.2012.01.015 (2012).
71 Morgan, A. R., Lam, W. J., Han, D. Y., Fraser, A. G. & Ferguson, L. R. Association Analysis of ULK1 with Crohn's Disease in a New Zealand Population. *Gastroenterology research and practice* **2012**, 715309, doi:10.1155/2012/715309 (2012).
72 Muise, A. M. *et al.* NADPH oxidase complex and IBD candidate gene studies: identification of a rare variant in NCF2 that results in reduced binding to RAC2. *Gut* **61**, 1028-1035, doi:10.1136/gutjnl-2011-300078 (2012).
73 Chen, J. *et al.* Crohn's disease and polymorphism of heat shock protein gene HSP70-2 in the Chinese population. *Journal of gastroenterology and hepatology* **28**, 814-818, doi:10.1111/jgh.12163 (2013).
74 Falvey, J. D. *et al.* Macrophage migration inhibitory factor gene polymorphisms in inflammatory bowel disease: an association study in New Zealand Caucasians and meta-analysis. *World journal of gastroenterology* **19**, 6656-6664, doi:10.3748/wjg.v19.i39.6656 (2013).
75 Hirano, A. *et al.* Association study of 71 European Crohn's disease susceptibility loci in a Japanese population. *Inflammatory bowel diseases* **19**, 526-533, doi:10.1097/MIB.0b013e31828075e7 (2013).
76 Luo, Y. Y. *et al.* [Association between vitamin D receptor gene polymorphisms and pediatric Crohn's disease in China: a study based on gene sequencing]. *Zhongguo dang dai er ke za zhi = Chinese journal of contemporary pediatrics* **15**, 1006-1008 (2013).
77 Wagner, J. *et al.* TLR4, IL10RA, and NOD2 mutation in paediatric Crohn's disease patients: an association with Mycobacterium avium subspecies paratuberculosis and TLR4 and IL10RA expression. *Medical microbiology and immunology* **202**, 267-276, doi:10.1007/s00430-013-0290-5 (2013).
78 Dhillon, S. S. *et al.* Higher activity of the inducible nitric oxide synthase contributes to very early onset inflammatory bowel disease. *Clin Transl Gastroenterol* **5**, e46, doi:10.1038/ctg.2013.17 (2014).
79 Hu, J. *et al.* Association of MYO9B gene polymorphisms with inflammatory bowel disease in Chinese Han population. *World journal of gastroenterology* **20**, 7466-7472, doi:10.3748/wjg.v20.i23.7466 (2014).
80 Jakobsen, C. *et al.* Genetic susceptibility and genotype-phenotype association in 588 Danish children with inflammatory bowel disease. *Journal of Crohn's & colitis* **8**, 678-685 (2014).
81 Serbati, N., Senhaji, N., Diakite, B., Badre, W. & Nadifi, S. IL23R and ATG16L1 variants in Moroccan patients with inflammatory bowel disease. *BMC research notes* **7**, 570, doi:10.1186/1756-0500-7-570 (2014).
82 Serbati, N., Badre, W., Diakite, B. & Nadifi, S. NOD2/CARD15 gene influences disease behaviour but not IBD susceptibility in a Moroccan population. *Turkish Journal of Gastroenterology* **25 Suppl 1**, 122-128 (2014).
83 Senhaji, N. *et al.* Genetic Polymorphisms of Multidrug Resistance Gene-1 (MDR1/ABCB1) and Glutathione S-Transferase Gene and the Risk of Inflammatory Bowel Disease among Moroccan Patients. *Mediators of inflammation* **2015**, 248060, doi:10.1155/2015/248060 (2015).
84 Schnitzler, F. *et al.* The NOD2 single nucleotide polymorphism rs72796353 (IVS4+10 A>C) is a predictor for perianal fistulas in patients with Crohn's disease in the absence of other NOD2 mutations. *PloS one* **10 (7) (no pagination)** (2015).
85 Adams, A. T. *et al.* Two-stage genome-wide methylation profiling in childhood-onset Crohn's Disease implicates epigenetic alterations at the VMP1/MIR21 and HLA loci. *Inflammatory bowel diseases* **20**, 1784-1793 (2014).
86 Harris, R. A. *et al.* Genome-wide peripheral blood leukocyte DNA methylation microarrays identified a single association with inflammatory bowel diseases. *Inflammatory bowel diseases* **18**, 2334-2341 (2012).
87 Harris, R. A. *et al.* DNA methylation-associated colonic mucosal immune and defense responses in treatmentnaive pediatric ulcerative colitis. *Epigenetics* **9**, 1131-1137 (2014).
88 Koukos, G. *et al.* MicroRNA-124 regulates STAT3 expression and is down-regulated in colon tissues of pediatric patients with ulcerative colitis. *Gastroenterology* **145**, 842-852.e842 (2013).
89 Koukos, G. *et al.* MicroRNA-4284 regulates CXCL5 expression and is down-regulated in colon tissues of pediatric patients with ulcerative colitis. *Gastroenterology* **1)**, S-781 (2015).
